# Supplementary material for: Genomic DNA extraction optimization and validation for genome sequencing using the marine gastropod Kellet’s whelk
Source: PeerJ. 2023 Dec 6;11:e16510. doi: 10.7717/peerj.16510 (PMC10710129; doi:10.7717/peerj.16510)
Supplement: Supplemental Information 7 [file peerj-11-16510-s007.zip › PCI Protocol.pdf]

Phenol/Chloroform gDNA Extraction Procedure

Materials

Proteinase K (20 mg/ml)
1 % SDS cell lysis buffer (100mM Tris-Cl; 50 mM EDTA; 1 % SDS)
7.5 M ammonium acetate
0.5 M EDTA
Nuclease-free water
Phenol:Chloroform:Isoamyl Alcohol (25:24:1)
Glycogen (20ug/µl)
70% Ethanol
100% Ethanol
Elution Buffer

Protocol

Sample preparation

1. Remove sample from preservative and dap on blue roll to remove excess ethanol (if required).
2. Add samples (up to 30 mg) to mortar and pestle, crush quickly and place in a 1.5mL test tube containing digestion reagents.

Digestion

4. Add the following to each tube before sample prep:
   a. 160 µl 1 % SDS cell lysis buffer.
   b. 20 µl 0.5 M EDTA.
   c. 20 µl proteinase K.
5. Mix by 1000 µl pipet. Spin down. Incubate at 65ºC for 1 hours.

***Add 25 µl RNase A. Mix by 1000 µl pipet. Incubate at 37ºC for 30 minutes. (optional) (I did not do this recently and found large tRNA bands in extractions)

DNA/RNA Separation

6. Add one volume of PCI to sample and shake by hand or vortex thoroughly for 20 seconds.
7. Centrifuge for 5 minutes at 16,000 x g.
8. Carefully remove the upper aqueous phase and transfer to a new tube, should be around ~180 µl (do not carry over any phenol!).

Precipitation of DNA

9. Add 1 µl of Glycogen (20ug/µl). (used for co-precipitation pellet - increased visual pellet)
10. Add .5x volume (if 180µl, then add 90µl) of 7.5M Ammonium Acetate.
11. Add 2.5x volume (if 270µl, then add 675µl) of 100% Ethanol, mix by pipet.
12. Place the tube at -20°C overnight.
13. Centrifuge the sample 4°C for 30 minutes at 16,000 x g to pellet DNA.
14. Remove supernatant without disturbing the pellet.

Washing of DNA

15. Add 150 µl of 70% Ethanol.
16. Centrifuge the sample at 4°C for 2 minutes at 16,000 x g, and remove supernatant.
17. Repeat stamps 15 and 16, Make sure to remove all Ethanol from the tube.
18. Dry DNA in speedvac for 2 minutes (if there is still ethanol or any liquid dry for another minute in speedvac).
19. Resuspend DNA in 200 µl of EB and let it sit in the fridge overnight.
20. Spin down sample to collect and store at -20°C.
