# Supplementary material for: Genomic DNA extraction optimization and validation for genome sequencing using the marine gastropod Kellet’s whelk
Source: PeerJ. 2023 Dec 6;11:e16510. doi: 10.7717/peerj.16510 (PMC10710129; doi:10.7717/peerj.16510)
Supplement: Supplemental Information 7 [file peerj-11-16510-s007.zip › Illumina NEBNext Ultra II DNA Library Prep Kit.pdf]

INSTRUCTION MANUAL

NEBNext® Ultra™ II DNA Library Prep Kit for Illumina®

NEB #E7645S/L, #E7103S/L

24/96 reactions

Version 7.0_9/22

Table of Contents

Overview....3

Protocol....4

NEBNext End Prep....4

Adaptor Ligation....4

Size Selection or Cleanup of Adaptor-ligated DNA....5

Size Selection of Adaptor-ligated DNA....5

Cleanup of Adaptor-ligated DNA without Size Selection....7

PCR Enrichment of Adaptor-ligated DNA....8

Cleanup of PCR Reaction....10

Kit Components....12

Revision History....13

The Library Kit Includes

The volumes provided are sufficient for preparation of up to 24 reactions (NEB #E7645S/#E7103S) and 96 reactions (NEB #E7645L/#E7103L). All reagents should be stored at –20°C. Colored bullets represent the color of the cap of the tube containing the reagent.

Package 1: Store at –20°C.

- (green) NEBNext Ultra II End Prep Enzyme Mix
- (green) NEBNext Ultra II End Prep Reaction Buffer
- (red) NEBNext Ultra II Ligation Master Mix
- (red) NEBNext Ligation Enhancer
- (blue) NEBNext Ultra II Q5 Master Mix

Package 2: Store at room temperature. Do not freeze.

Supplied only with NEBNext Ultra II DNA Library Prep with Sample Purification Beads, NEB #E7103.

NEBNext Sample Purification Beads

Required Materials Not Included

- NEBNext Oligo Kit options can be found at www.neb.com/oligos

  Alternatively, customer supplied adaptor and primers can be used, please see information in link below:
  https://www.neb.com/faqs/2019/03/08/can-i-use-this-nebnext-kit-with-adaptors-and-primers-from-other-vendors-than-neb

Please note: Separate instructions exist for UNIQUE DUAL INDEX UMI ADAPTORS. Please contact Technical Support at info@neb.com

- Magnetic rack (NEB #S1515S), magnetic plate (Alpaqua® cat. #A001322) or equivalent
- 80% Ethanol (freshly prepared)
- Nuclease-free Water
- 0.1X TE (1 mM Tris-HCl, pH 8.0, 0.1 mM EDTA)
- Thin wall 200 µl PCR tubes (For example Tempassure PCR flex-free 8-tube strips USA Scientific #1402-4708)
- PCR machine

- Bioanalyzer®, TapeStation® (Agilent Technologies, Inc.) or similar fragment analyzer and consumables

For NEB #E7645 only:

- SPRIselect® Reagent Kit (Beckman Coulter, Inc. #B23317) or AMPure® XP Beads (Beckman Coulter, Inc. #A63881)

Optional:

- 10 mM Tris-HCl, pH 7.5-8.0 with 10 mM NaCl (for adaptor dilution of DNA input < 100 ng) or NEB #B1430S

Overview

The NEBNext Ultra II DNA Library Prep Kit for Illumina contains the enzymes and buffers required to convert a broad range of input amounts of DNA into high quality libraries for next-generation sequencing on the Illumina platform. The fast, user-friendly workflow also has minimal hands-on time.

Each kit component must pass rigorous quality control standards, and for each new lot the entire set of reagents is functionally validated together by construction and sequencing of an indexed library on the Illumina sequencing platform.

For larger volume requirements, customized and bulk packaging is available by purchasing through the OEM/Bulks department at NEB. Please contact OEM@neb.com for further information.

Figure 1. Workflow demonstrating the use of NEBNext Ultra II DNA Library Prep Kit for Illumina

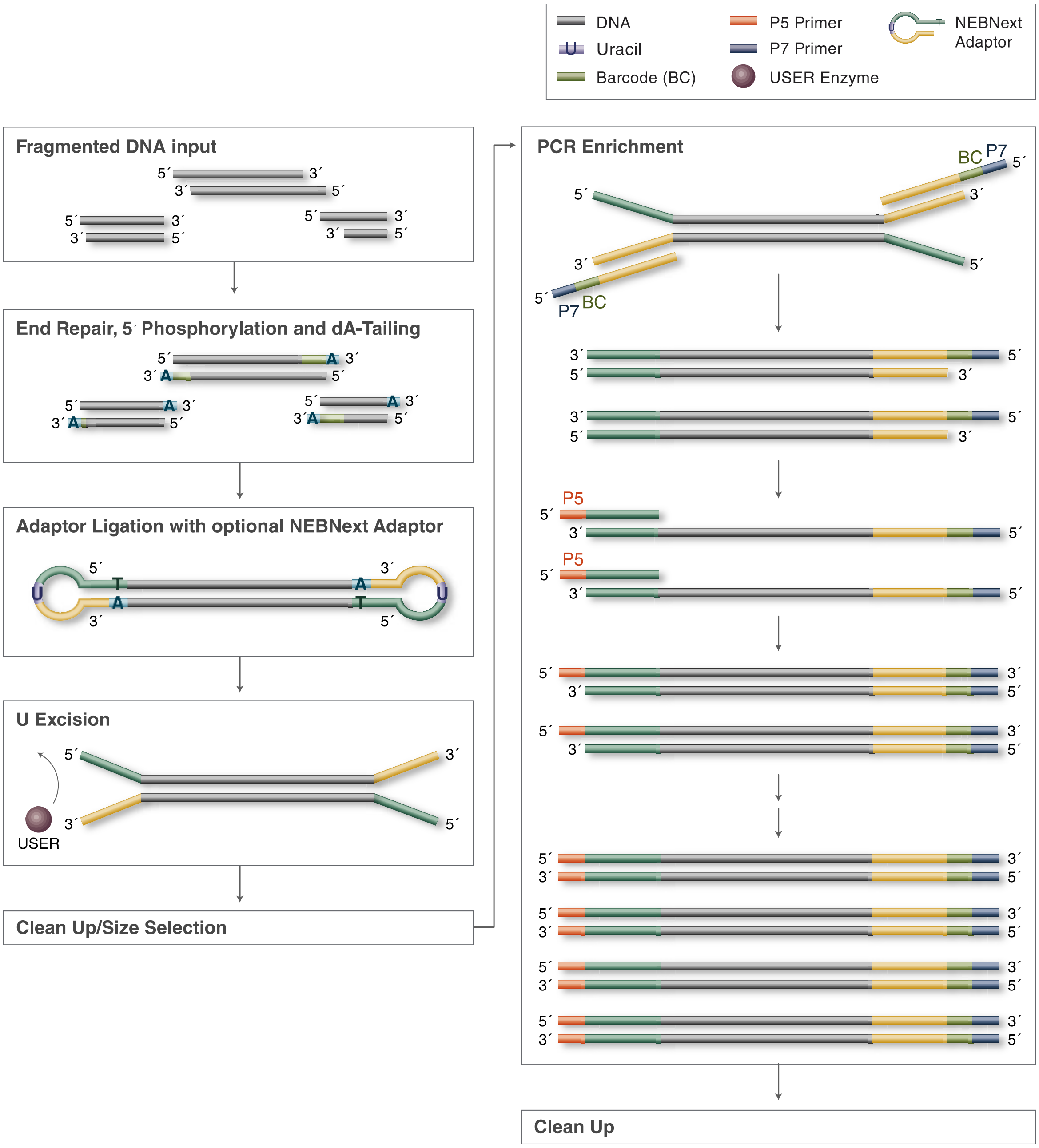

Adaptor trimming sequences:

The NEBNext libraries for Illumina resemble TruSeq libraries and can be trimmed similar to TruSeq:

Adaptor Read 1 AGATCGGAAGAGCACACGTCTGAACTCCAGTCA

Adaptor Read 2 AGATCGGAAGAGCGTCGTGTAGGGAAAGAGTGT

Protocol

Symbols

This is a point where you can safely stop the protocol.

This caution sign signifies a step in the protocol that has two paths leading to the same end point but is dependent on a user variable, like the amount of input DNA.

●

Colored bullets indicate the cap color of the reagent to be added to a reaction.

Starting Material: 500 pg–1 µg fragmented DNA. NEB recommends that DNA be sheared in 1X TE. If the DNA volume post shearing is less than 50 µl, add 1X TE to a final volume of 50 µl. Alternatively, samples can be diluted with 10 mM Tris-HCl, pH 8.0 or 0.1X TE.

1. NEBNext End Prep

1.1. Add the following components to a sterile nuclease-free tube:

COMPONENT	VOLUME
● (green) NEBNext Ultra II End Prep Enzyme Mix	3 µl
● (green) NEBNext Ultra II End Prep Reaction Buffer	7 µl
Fragmented DNA	50 µl
Total Volume	60 µl

1.2. Set a 100 µl or 200 µl pipette to 50 µl and then pipette the entire volume up and down at least 10 times to mix thoroughly. Perform a quick spin to collect all liquid from the sides of the tube.

Note: It is important to mix well. The presence of a small amount of bubbles will not interfere with performance.

1.3. Place in a thermal cycler, with the heated lid set to ≥ 75°C, and run the following program:

30 minutes @ 20°C

30 minutes @ 65°C

Hold at 4°C

If necessary, samples can be stored at –20°C; however, a slight loss in yield (~20%) may be observed. NEB recommends continuing with adaptor ligation before stopping.

2. Adaptor Ligation

2.1 Determine whether adaptor dilution is necessary.

If DNA input is ≤ 100 ng, dilute the NEBNext Adaptor for Illumina in 10 mM Tris-HCl, pH 7.5-8.0 with 10 mM NaCl as indicated in Table 2.1.

Table 2.1: Adaptor Dilution

INPUT	ADAPTOR DILUTION (VOLUME OF ADAPTOR: TOTAL VOLUME)	WORKING ADAPTOR CONCENTRATION
1 µg–101 ng	No Dilution	15 µM
100 ng–5 ng	10-Fold (1:10)	1.5 µM
less than 5 ng	25-Fold (1:25)	0.6 µM

Note: The appropriate adaptor dilution for your sample input and type may need to be optimized experimentally. The dilutions provided here are a general starting point. Excess adaptor should be removed prior to PCR enrichment.

2.2. Add the following components directly to the End Prep Reaction Mixture:

COMPONENT	VOLUME
End Prep Reaction Mixture (Step 1.3 in Section 1)	60 µl
• (red) NEBNext Adaptor for Illumina**	2.5 µl
• (red) NEBNext Ultra II Ligation Master Mix*	30 µl
• (red) NEBNext Ligation Enhancer	1 µl
Total Volume	93.5 µl

* Mix the Ultra II Ligation Master Mix by pipetting up and down several times prior to adding to the reaction.

** The NEBNext adaptor is provided in NEBNext Oligo kits. NEB has several oligo options which are supplied separately from the library prep kit. Please see www.neb.com/oligos for additional information.

Note: The Ligation Master Mix and Ligation Enhancer can be mixed ahead of time and is stable for at least 8 hours @ 4°C. Do not premix the Ligation Master Mix, Ligation Enhancer and adaptor prior to use in the Adaptor Ligation Step.

2.3. Set a 100 µl or 200 µl pipette to 80 µl and then pipette the entire volume up and down at least 10 times to mix thoroughly. Perform a quick spin to collect all liquid from the sides of the tube. **(Caution: The NEBNext Ultra II Ligation Master Mix is very viscous. Care should be taken to ensure adequate mixing of the ligation reaction, as incomplete mixing will result in reduced ligation efficiency. The presence of a small amount of bubbles will not interfere with performance).**

2.4. Incubate at 20°C for 15 minutes in a thermal cycler with the heated lid off.

2.5. Add 3 µl of • (red or blue) USER® Enzyme to the ligation mixture from Step 2.3.

Note: Steps 2.5. and 2.6. are only required for use with non indexed NEBNext Adaptor. USER enzyme can be found in most NEBNext oligo kits. If you are using the indexed UMI adaptor, USER is not needed. Please see corresponding manual for use with UMI on the NEB #E7395 product page under the protocols, manuals, and usage tab.

2.6. Mix well and incubate at 37°C for 15 minutes with the heated lid set to ≥ 47°C.

Samples can be stored overnight at –20°C.

3. Size Selection or Cleanup of Adaptor-ligated DNA

If the starting material is > 50 ng, follow the protocol for size selection in Section 3A. For input ≤ 50 ng, size selection is not recommended to maintain library complexity. Follow the protocol for cleanup without size selection in Section 3B.

3A. Size Selection of Adaptor-ligated DNA

Note: The following section is for cleanup of the ligation reaction. The volumes of SPRIselect or NEBNext Sample Purification Beads provided here are for use with the sample contained in the exact buffer at this step. AMPure XP Beads can be used as well. If using AMPure XP Beads, allow the beads to warm to room temperature for at least 30 minutes before use. These bead volumes may not work properly for a cleanup at a different step in the workflow, or if this is a second cleanup at this step. For cleanups of samples contained in different buffer conditions, the volumes may need to be experimentally determined.

The following size selection protocol is for libraries with 200 bp inserts only. For libraries with different size fragment inserts, refer to the table below for the appropriate volumes of beads to be added. The size selection protocol is based on starting volume of 96.5 µl.

To select a different insert size than 200 bp, please use the volumes in this table:

Table 3.1: Recommended Conditions for Bead Based Size Selection

LIBRARY PARAMETERS	APPROXIMATE INSERT SIZE DISTRIBUTION	**150 bp**	**200 bp**	250 bp	300-400 bp	400-500 bp	500-700 bp
	Approx. Final Library Size Distribution (insert + adaptor + primers)	270 bp	320 bp	370 bp	480 bp	600 bp	750-800 bp
BEAD VOLUME TO BE ADDED (µl)	1st Bead Addition	50	40	30	25	20	15
	2nd Bead Addition	25	20	15	10	10	10

3A.1. Vortex SPRIselect or NEBNext Sample Purification Beads to resuspend.

3A.2. Add 40 µl (~ 0.4X) of resuspended beads to the 96.5 µl ligation reaction. Mix well by pipetting up and down at least 10 times. Be careful to expel all of the liquid out of the tip during the last mix. Vortexing for 3-5 seconds on high can also be used. If centrifuging samples after mixing, be sure to stop the centrifugation before the beads start to settle out.

3A.3. Incubate samples on bench top for at least 5 minutes at room temperature.

3A.4. Place the tube/plate on an appropriate magnetic stand to separate the beads from the supernatant. If necessary, quickly spin the sample to collect the liquid from the sides of the tube or plate wells before placing on the magnetic stand.

3A.5. After 5 minutes (or when the solution is clear), carefully transfer the supernatant containing your DNA to a new tube **(Caution: do not discard the supernatant)**. Discard the beads that contain the unwanted large fragments.

3A.6. Add 20 µl (0.2X) resuspended SPRIselect or NEBNext Sample Purification Beads to the supernatant and mix at least 10 times. Be careful to expel all of the liquid from the tip during the last mix. Then incubate samples on the bench top for at least 5 minutes at room temperature.

3A.7. Place the tube/plate on an appropriate magnetic stand to separate the beads from the supernatant. If necessary, quickly spin the sample to collect the liquid from the sides of the tube or plate wells before placing on the magnetic stand.

3A.8. After 5 minutes (or when the solution is clear), carefully remove and discard the supernatant that contains unwanted DNA. Be careful not to disturb the beads that contain the desired DNA targets **(Caution: do not discard beads)**.

3A.9. Add 200 µl of 80% freshly prepared ethanol to the tube/plate while in the magnetic stand. Incubate at room temperature for 30 seconds, and then carefully remove and discard the supernatant. Be careful not to disturb the beads that contain DNA targets.

3A.10. Repeat Step 3A.9 once for a total of two washes. Be sure to remove all visible liquid after the second wash. If necessary, briefly spin the tube/plate, place back on the magnet and remove traces of ethanol with a p10 pipette tip.

3A.11. Air dry the beads for up to 5 minutes while the tube/plate is on the magnetic stand with the lid open.

Caution: Do not overdry the beads. This may result in lower recovery of DNA target. Elute the samples when the beads are still dark brown and glossy looking, but when all visible liquid has evaporated. When the beads turn lighter brown and start to crack, they are too dry.

3A.12. Remove the tube/plate from the magnetic stand. Elute the DNA target from the beads into 17 µl of 10 mM Tris-HCl or 0.1X TE.

3A.13. Mix well on a vortex mixer or by pipetting up and down 10 times. Incubate for at least 2 minutes at room temperature. If necessary, quickly spin the sample to collect the liquid from the sides of the tube or plate wells before placing back on the magnetic stand.

3A.14. Place the tube/plate on a magnetic stand. After 5 minutes (or when the solution is clear), transfer 15 µl to a new PCR tube for (amplification).

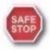

Samples can be stored at –20°C.

3B. Cleanup of Adaptor-ligated DNA without Size Selection (for input ≤ 50 ng)

The following section is for cleanup of the ligation reaction. If your input DNA is > 50 ng, follow the size selection protocol in Section 3A.

Note: The volumes of SPRIselect or NEBNext Sample Purification Beads provided here are for use with the sample contained in the exact buffer at this step. AMPure XP Beads can be used as well. If using AMPure XP Beads, allow the beads to warm to room temperature for at least 30 minutes before use. These bead volumes may not work properly for a cleanup at a different step in the workflow, or if this is a second cleanup at this step. For cleanups of samples contained in different buffer conditions, the volumes may need to be experimentally determined.

3B.1. Vortex SPRIselect or NEBNext Sample Purification Beads to resuspend.

3B.2. Add 87 µl (0.9X) resuspended beads to the Adaptor Ligation reaction. Mix well by pipetting up and down at least 10 times. Be careful to expel all of the liquid out of the tip during the last mix. Vortexing for 3-5 seconds on high can also be used. If centrifuging samples after mixing, be sure to stop the centrifugation before the beads start to settle out.

3B.3. Incubate samples on bench top for at least 5 minutes at room temperature.

3B.4. Place the tube/plate on an appropriate magnetic stand to separate the beads from the supernatant. If necessary, quickly spin the sample to collect the liquid from the sides of the tube or plate wells before placing on the magnetic stand.

3B.5. After 5 minutes (or when the solution is clear), carefully remove and discard the supernatant. Be careful not to disturb the beads that contain DNA targets **(Caution: do not discard beads).**

3B.6. Add 200 µl of 80% freshly prepared ethanol to the tube/ plate while in the magnetic stand. Incubate at room temperature for 30 seconds, and then carefully remove and discard the supernatant. Be careful not to disturb the beads that contain DNA targets.

3B.7. Repeat Step 3B.6 once for a total of two washes. Be sure to remove all visible liquid after the second wash. If necessary, briefly spin the tube/plate, place back on the magnet and remove traces of ethanol with a p10 pipette tip.

3B.8. Air dry the beads for up to 5 minutes while the tube/plate is on the magnetic stand with the lid open.

Caution: Do not over-dry the beads. This may result in lower recovery of DNA target. Elute the samples when the beads are still dark brown and glossy looking, but when all visible liquid has evaporated. When the beads turn lighter brown and start to crack, they are too dry.

3B.9. Remove the tube/plate from the magnetic stand. Elute the DNA target from the beads by adding 17 µl of 10 mM Tris-HCl or 0.1X TE.

3B.10. Mix well by pipetting up and down 10 times, or on a vortex mixer. Incubate for at least 2 minutes at room temperature. If necessary, quickly spin the sample to collect the liquid from the sides of the tube or plate wells before placing back on the magnetic stand.

3B.11. Place the tube/plate on the magnetic stand. After 5 minutes (or when the solution is clear), transfer 15 µl to a new PCR tube.

SAFE STOP

Samples can be stored at –20°C.

4. PCR Enrichment of Adaptor-ligated DNA

⚠

Follow Step 4.1.1A. if you are using the following oligos:
Use Option A for any NEBNext Oligo Kit where index primers are supplied in tubes. These kits have the forward and reverse primers supplied in separate tubes. Primers are supplied at 10 µM each.

Follow Step 4.1.1B. if you are using the following oligos:
Use Option B for any NEBNext Oligo Kit where index primers are supplied in a 96-well plate format. These kits have the forward and reverse (i7 and i5) primers combined. Primers are supplied at 10 µM combined (5 µM each).

4.1. PCR Amplification

4.1.1. Add the following components to a sterile strip tube:

4.1.1A. Forward and Reverse Primers not already combined

COMPONENT	VOLUME
Adaptor Ligated DNA Fragments (Step 3A.14. or 3B.11)	15 µl
• (blue) NEBNext Ultra II Q5 Master Mix	25 µl
• (blue) Index Primer/i7 Primer*,**	5 µl
• (blue) Universal PCR Primer/i5 Primer*,**	5 µl
Total Volume	50 µl

* NEBNext Oligos must be purchased separately from the library prep kit. For oligo purchasing options refer to "Required Materials Not Included" section (page 1). Refer to the corresponding NEBNext Oligo kit manual for determining valid barcode combinations.

** Use only one i7 primer/ index primer per sample. Use only one i5 primer (or the universal primer for single index kits) per sample

4.1.1B. Forward and Reverse Primers already combined

COMPONENT	VOLUME
Adaptor Ligated DNA Fragments (Step 3A.14. or 3B.11)	15 µl
• (blue) NEBNext Ultra II Q5 Master Mix	25 µl
• (blue) Index Primer Mix*	10 µl
Total Volume	50 µl

* NEBNext Oligos must be purchased separately from the library prep kit. For oligo purchasing options refer to "Required Materials Not Included" section (page 1).

4.1.2. Set a 100 µl or 200 µl pipette to 40 µl and then pipette the entire volume up and down at least 10 times to mix thoroughly. Perform a quick spin to collect all liquid from the sides of the tube.

4.1.3. Place the tube on a thermal cycler and perform PCR amplification using the following PCR cycling conditions:

CYCLE STEP	TEMP	TIME	CYCLES
Initial Denaturation	98°C	30 seconds	1
Denaturation	98°C	10 seconds	3-15*
Annealing/Extension	65°C	75 seconds	
Final Extension	65°C	5 minutes	1
Hold	4°C	∞	

* The number of PCR cycles should be chosen based on input amount and sample type. Thus, samples prepared with a different method prior to library prep may require re-optimization of the number of PCR cycles. The number of cycles should be high enough to provide sufficient library fragments for a successful sequencing run, but low enough to avoid PCR artifacts and over-cycling (high molecular weight fragments on Bioanalyzer). The number of PCR cycles recommended in Table 4.1 are to be seen as a starting point to determine the number of PCR cycles best for standard library prep samples. Use Table 4.2 for applications requiring high library yields (~1 µg) such as target enrichment.

Table 4.1.

INPUT DNA IN THE END PREP REACTION	# OF CYCLES REQUIRED FOR STANDARD LIBRARY PREP: YIELD ~100 ng (30–100 nM)*
1 µg*	3**
500 ng*	3**
100 ng*	3
50 ng	3–4
10 ng	6–7
5 ng	7–8
1 ng	9–10
0.5 ng	10–11

* These input ranges will work best when size selection is done

** NEBNext adaptors contain a unique truncated design. Libraries constructed with NEBNext adaptors require a minimum of 3 amplification cycles to add the complete adaptor sequences for downstream processes.

Table 4.2.

INPUT DNA IN THE END PREP REACTION	# OF CYCLES REQUIRED FOR TARGET ENRICHMENT LIBRARY PREP (~1 µg)
1 µg*	3–4*,**
500 ng*	4–5*
100 ng*	6–7*
50 ng	7–8
10 ng	9–10
5 ng	10–11
1 ng	12–13
0.5 ng	14–15

* Cycle number was determined for size selected libraries.

** NEBNext adaptors contain a unique truncated design. Libraries constructed with NEBNext adaptors require a minimum of 3 amplification cycles to add the complete adaptor sequences for downstream processes.

4.1.4. Proceed to Cleanup of PCR Amplification in Section 5.

5. Cleanup of PCR Reaction

Note: The volumes of SPRIselect or NEBNext Sample Purification Beads provided here are for use with the sample contained in the exact buffer at this step. AMPure XP beads can be used as well. If using AMPure XP beads, allow the beads to warm to room temperature for at least 30 minutes before use. These volumes may not work properly for a cleanup at a different step in the workflow. For cleanups of samples contained in different buffer conditions, the volumes may need to be experimentally determined.

5.1. Vortex SPRIselect or NEBNext Sample Purification Beads to resuspend.

5.2. Add 45 µl (0.9X) resuspended beads to the PCR reaction. Mix well by pipetting up and down at least 10 times. Be careful to expel all of the liquid out of the tip during the last mix. Vortexing for 3-5 seconds on high can also be used. If centrifuging samples after mixing, be sure to stop the centrifugation before the beads start to settle out.

5.3. Incubate samples on bench top for at least 5 minutes at room temperature.

5.4. Place the tube/plate on an appropriate magnetic stand to separate the beads from the supernatant. If necessary, quickly spin the sample to collect the liquid from the sides of the tube or plate wells before placing on the magnetic stand.

5.5. After 5 minutes (or when the solution is clear), carefully remove and discard the supernatant. Be careful not to disturb the beads that contain DNA targets **(Caution: do not discard the beads)**.

5.6. Add 200 µl of 80% freshly prepared ethanol to the tube/plate while in the magnetic stand. Incubate at room temperature for 30 seconds, and then carefully remove and discard the supernatant. Be careful not to disturb the beads that contain DNA targets.

5.7. Repeat Step 5.6. once for a total of two washes. Be sure to remove all visible liquid after the second wash. If necessary, briefly spin the tube/plate, place back on the magnet and remove traces of ethanol with a p10 pipette tip.

5.8. Air dry the beads for up to 5 minutes while the tube/plate is on the magnetic stand with the lid open.

Caution: Do not over-dry the beads. This may result in lower recovery of DNA. Elute the samples when the beads are still dark brown and glossy looking, but when all visible liquid has evaporated. When the beads turn lighter brown and start to crack, they are too dry.

5.9. Remove the tube/plate from the magnetic stand. Elute the DNA target from the beads by adding 33 µl of 0.1X TE.

5.10. Mix well by pipetting up and down 10 times, or on a vortex mixer. Incubate for at least 2 minutes at room temperature. If necessary, quickly spin the sample to collect the liquid from the sides of the tube or plate wells before placing back on the magnetic stand.

5.11. Place the tube/plate on the magnetic stand. After 5 minutes (or when the solution is clear), transfer 30 µl to a new PCR tube and store at –20°C.

5.12 Check the size distribution on an Agilent Bioanalyzer High Sensitivity DNA chip. The sample may need to be diluted before loading.

Samples can be stored at –20°C.

Figure 5.1: Examples of libraries prepared with human DNA (NA19240).

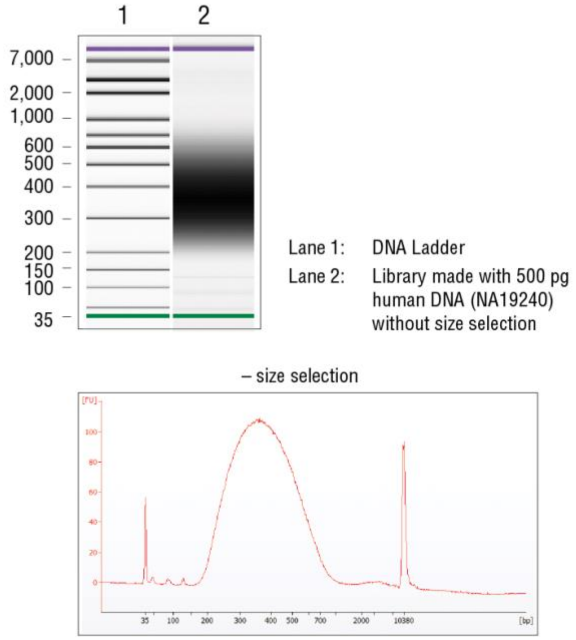

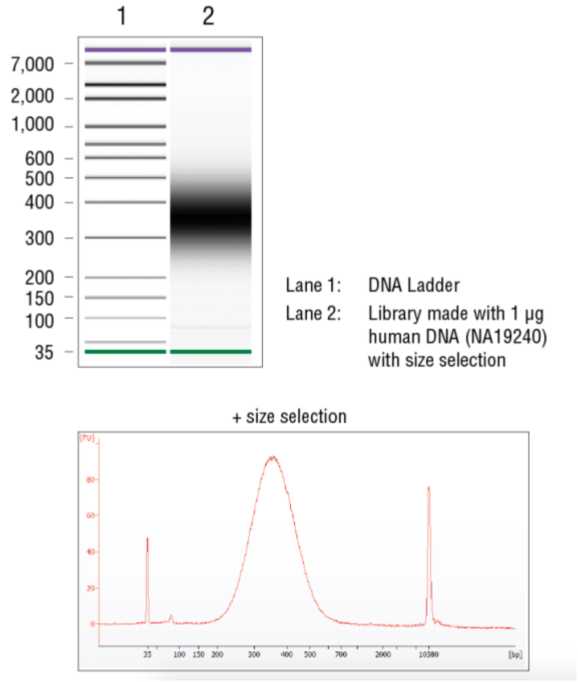

Kit Components

NEB #E7645S Table of Components

NEB #	PRODUCT	VOLUME
E7646A	NEBNext Ultra II End Prep Enzyme Mix	0.072 ml
E7647A	NEBNext Ultra II End Prep Reaction Buffer	0.168 ml
E7648A	NEBNext Ultra II Ligation Master Mix	0.720 ml
E7374A	NEBNext Ligation Enhancer	0.024 ml
E7649A	NEBNext Ultra II Q5 Master Mix	0.6 ml

NEB #E7645L Table of Components

NEB #	PRODUCT	VOLUME
E7646AA	NEBNext Ultra II End Prep Enzyme Mix	0.288 ml
E7647AA	NEBNext Ultra II End Prep Reaction Buffer	0.672 ml
E7648AA	NEBNext Ultra II Ligation Master Mix	2.88 ml
E7374AA	NEBNext Ligation Enhancer	0.096 ml
E7649AA	NEBNext Ultra II Q5 Master Mix	2.4 ml

NEB #E7103S Table of Components

NEB #	PRODUCT	VOLUME
E7646A	NEBNext Ultra II End Prep Enzyme Mix	0.072 ml
E7647A	NEBNext Ultra II End Prep Reaction Buffer	0.168 ml
E7648A	NEBNext Ultra II Ligation Master Mix	0.720 ml
E7374A	NEBNext Ligation Enhancer	0.024 ml
E7649A	NEBNext Ultra II Q5 Master Mix	0.6 ml
E7104S	NEBNext Sample Purification Beads	4 ml

NEB #E7103L Table of Components

NEB #	PRODUCT	VOLUME
E7646AA	NEBNext Ultra II End Prep Enzyme Mix	0.288 ml
E7647AA	NEBNext Ultra II End Prep Reaction Buffer	0.672 ml
E7648AA	NEBNext Ultra II Ligation Master Mix	2.88 ml
E7374AA	NEBNext Ligation Enhancer	0.096 ml
E7649AA	NEBNext Ultra II Q5 Master Mix	2.4 ml
E7104L	NEBNext Sample Purification Beads	16 ml

Revision History

REVISION #	DESCRIPTION	DATE
1.1	Figure 1.1 on page 12: human DNA (NA10240) was changed to human DNA (NA19240)	
2.0	Protocol updated to include NEB #E7710 and NEB #E7730	6/16
3.0	Additional clarifying information and formatting changes. Adaptor recommended to dilute only in Tris-HCl with NaCl. Size selection and cleanup steps clarified. 25 µM index primer removed from PCR section. Checklist style protocol added. Protocol updated to include NEB #E7710 and NEB #E7730. Section C in the PCR setup step was removed because all of the 25 µM primers are now expired.	7/16
3.1	Table 3.1 on page 7, "average size of final library" row numbers adjusted.	9/16
3.2	Step 4.1.3. Table 4.1 Minimum number of PCR cycles was adjusted.	10/16
3.3	Step 4.1.3. Update PCR cycles Table 4.1 Combine manual with #E7103. Place updated workflow diagram. Edit protocol text.	7/17
4.0	Create "Kit Component – Table of Components" for small and large size kits. Delete individual component information pages.	4/18
5.0	Update Table 4.2 # of cycles required 100 ng row and the 50 ng row.	6/18
6.0	Convert manual to new template format	4/20
6.1	Updated product license information	5/20
7.0	Updated required materials and protocol	9/22

This product is intended for research purposes only. This product is not intended to be used for therapeutic or diagnostic purposes in humans or animals.
This product is covered by one or more patents, trademarks and/or copyrights owned or controlled by New England Biolabs, Inc. For more information about commercial rights, please email us at busdev@neb.com. While NEB develops and validates its products for various applications, the use of this product may require the buyer to obtain additional third party intellectual property rights for certain applications.

This product is licensed for research and commercial use from Bio-Rad Laboratories, Inc., under U.S. Pat. Nos. 6,627,424, 7,541,170, 7,670,808, 7,666,645, and corresponding patents in other countries. No rights are granted for use of the product for Digital PCR or real-time PCR applications, with the exception of quantification in Next Generation Sequencing workflows.

ALPAQUA® is a registered trademark of Alpaqua Engineering, LLC.
B CORPORATION® is a registered trademark of B Lab, Inc.
MICROSEAL® is a registered trademark of Bio-Rad.
ILLUMINA® and MISEQ® are registered trademarks of Illumina, Inc.
BIOANALYZER® is a registered trademark of Agilent Technologies
AMPURE® and SPRISELECT® are registered trademarks of Beckman Coulter, Inc.
USA SCIENTIFIC® is a registered trademark of USA Scientific, Inc.

© Copyright 2022, New England Biolabs, Inc.; all rights reserved.

ISO 9001 Registered Quality Management | ISO 14001 Registered Environmental Management | ISO 13485 Registered Medical Devices | Certified B Corporation

www.neb.com

NEW ENGLAND BioLabs® Inc.

be INSPIRED drive DISCOVERY stay GENUINE

New England Biolabs, Inc., 240 County Road, Ipswich, MA 01938-2723 Telephone: (978) 927-5054 Toll Free: (USA Orders) 1-800-632-5227 (USA Tech) 1-800-632-7799 Fax: (978) 921-1350 e-mail: info@neb.com
