# Supplementary material for: Genomic DNA extraction optimization and validation for genome sequencing using the marine gastropod Kellet’s whelk
Source: PeerJ. 2023 Dec 6;11:e16510. doi: 10.7717/peerj.16510 (PMC10710129; doi:10.7717/peerj.16510)
Supplement: Supplemental Information 7 [file peerj-11-16510-s007.zip › Zymo Quick-DNA HMW Magbead kit.pdf]

ZYMO RESEARCH

The Beauty of Science is to Make Things Simple

INSTRUCTION MANUAL

Quick-DNA™ HMW MagBead Kit

Catalog No. **D6060**

Highlights

- **High Molecular Weight DNA:** Extract high molecular weight DNA up to 150 kb from any sample.
- **Ultra-Pure**: Highest DNA yield and purity equipped with RNA removal technology.
- **Third-Generation Sequencing Ready**: Optimized for long read sequencing (including Oxford Nanopore™ and PacBio SMRT™ sequencing).

Contents

Product Contents 1
Product Specifications.................................. 1
Product Description...................................2-3
Purification Guide.. 4
Reagent Preparation 5
Protocol 5-6
Appendix A: Enzymatic Digestion of Microbes......7
Appendix B: Optional RNase Treatment...............8
Troubleshooting Guide.................................. 9
Ordering Information 10

For Research Use Only

Ver. 1.0.1

Notes:

Satisfaction of all Zymo Research products is guaranteed. If you are dissatisfied with this product, please call 1-888-882-9682.

This product is for research use only and should only be used by trained professionals. It is not for use in diagnostic procedures. Some reagents included with this kit are irritants. Wear protective gloves and eye protection. Follow the safety guidelines and rules enacted by your research institution or facility.

™ Trademarks of Zymo Research Corporation. Nanopore™ is a trademark of Oxford Nanopore Technologies. Qubit™and NanoDrop™ are trademarks of Thermo Fisher Scientific. TapeStation® is a registered trademark of Agilent Technologies, Inc.

Product Contents

***Quick*-DNA™ HMW MagBead Kit** (Kit Size)	**D6060** (96 Preps.)	**Storage Temperature**
**Proteinase K & Storage Buffer**[1]	2 x 20 mg	-20°C (after mixing)
**Biofluid & Solid Tissue Buffer**	25 ml	Room Temp.
***Quick*-DNA™ MagBinding Buffer**	150 ml	Room Temp.
**DNA Pre-Wash Buffer**[2]	50 ml	Room Temp.
**g-DNA Wash Buffer**	1 x 200 ml	Room Temp.
**DNA Elution Buffer**	50 ml	Room Temp.
**MagBinding Beads**	8 ml	Room Temp.

Note - Integrity of kit components is guaranteed for up to one year from date of purchase. Reagents are routinely tested on a lot-to-lot basis to ensure they provide maximal performance and reliability.

[1] Prior to use, reconstitute the lyophilized **Proteinase K** with 1040 µl **Proteinase K Storage Buffer**. Vortex to dissolve. Store at -20ºC.
[2] A precipitate may have formed in the **DNA Pre-Wash Buffer** during shipping. To completely resuspend the buffer, incubate the bottle at 30 – 37 ºC for 30 minutes and mix by inversion. DO NOT MICROWAVE.

Specifications

- **Sample Types** – Any cells, solid tissue, whole blood, saliva, biological fluids, buccal, swabs, stool, microbiome samples, samples stored in DNA/RNA Shield™, *etc.*
- **DNA Purity** - High quality DNA is ready for all sensitive downstream applications such as long read sequencing, PCR, endonuclease digestion, Southern blotting, genotyping, Next-Generation Sequencing, bisulfite conversion, *etc.* ($A_{260}/A_{230} \geq 1.8$).
- **DNA Yield** - The DNA binding capacity is 10 µg per 50 µl MagBinding Beads used.
- **DNA Size** - Capable of recovering genomic and mitochondrial DNA sized fragments up to 150 kb. If present, plasmid, parasitic, microbial, and viral DNA will also be recovered.
- **Elution Volume** - 50 µl **DNA Elution Buffer** per 33 µl MagBinding Beads.
- **Equipment:** Magnetic rack, shaker and/or rotator, and microcentrifuge (optional)
- **Automation:** For assistance with automating/scripting this workflow onto your device, contact one of our automation experts at automation@zyomresearch.com.

Product Description

The ***Quick*-DNA™ HMW MagBead Kit** is the easiest method for high molecular weight total DNA extraction (e.g., genomic, mitochondrial, viral) from any biological fluid, cell culture, solid tissue, or environmental sample. Innovative reagents and Zymo Research's unique system allow for a simple Bind, Wash, & Elute procedure that is unmatched in providing ultra-pure and high yield DNA of > 150 kb size. Isolated DNA is ready for immediate use in sensitive downstream applications including long read (Oxford Nanopore™) and NGS sequencing, qPCR, arrays, and methylation analysis.

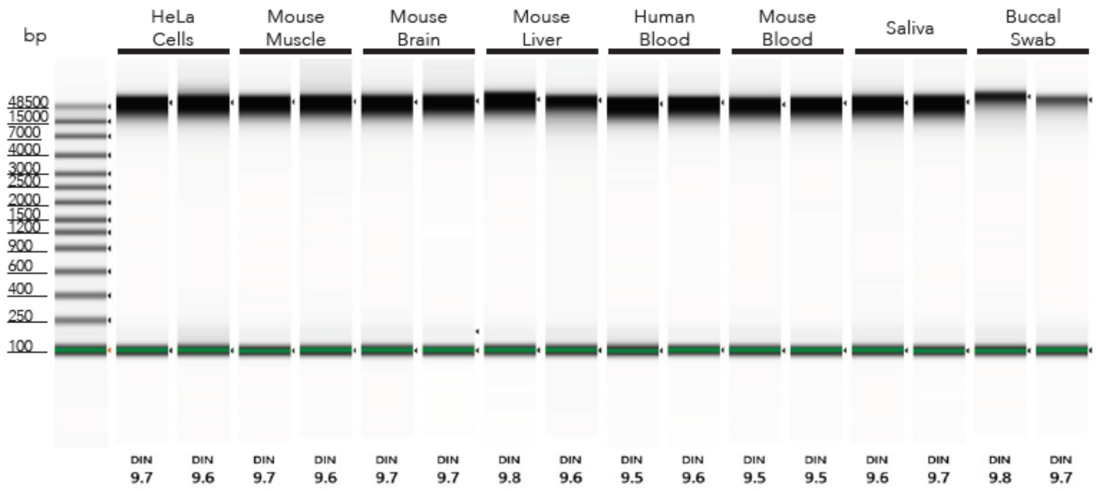

High Molecular Weight DNA From Any Sample Type. 10^6 Mammalian HeLa cells, 25 mg mouse muscle, brain, and liver, 200 µl human blood, 200 µl mouse blood, 200 µl human saliva, and buccal swabs stored in DNA/RNA Shield (R1100-50) were extracted using the *Quick*-DNA™ HMW Magbead Kit (n=2). DNA is of high molecular weight size (>60 kb). Quality was assessed using Agilent 2200 TapeStation®.

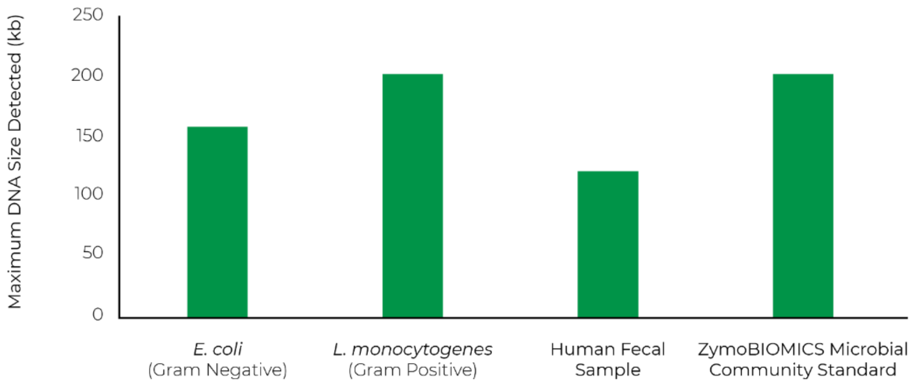

High Molecular Weight DNA. Cultured *E. coli* (~10^8 cells), cultured *L. monocytogenes* (~10^8 cells), 50 mg human feces, and 75 µl ZymoBIOMICS™ Microbial Community Standard (D6300) were input into the *Quick*-DNA™ HMW MagBead kit (n=2). Length of the highest detected peak were recorded and averaged for each sample. DNA size was analyzed using Agilent's Femto Pulse system.

Notes:

For **Technical Assistance**, please contact **Zymo** at 1-888-882-9682 or E-mail tech@zymoresearch.com.

Notes:

Product Description (Continued)

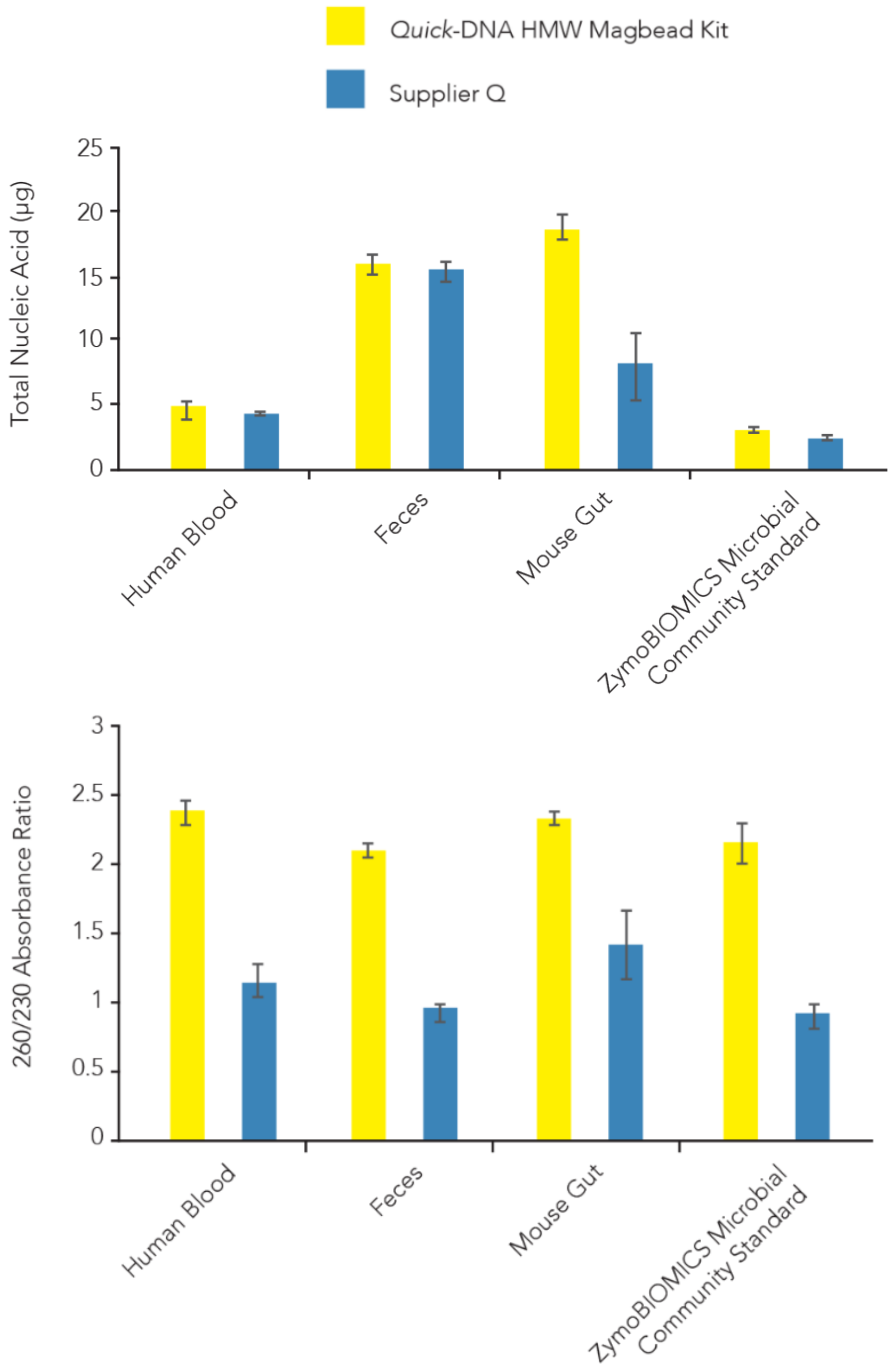

Ultra-Pure. 200 µl human blood, 50 mg feces, 25 mg mouse gut, and 75 µl ZymoBIOMICS™ Microbial Community Standard (D6300) were processed using the Quick-DNA™ HMW MagBead kit and Supplier Q kits (n=2). Zymo Research consistently had higher purities (A260/230: >1.8) at comparable DNA recovery (µg). Absorbance A260/230, and total DNA recovery (µg) were quantified by NanoDrop™ 2000.

The ***Quick*-DNA™ HMW MagBead Kit** facilitates rapid and efficient purification of high molecular weight DNA from any sample by combining enzymatic and chemical extraction regimens.

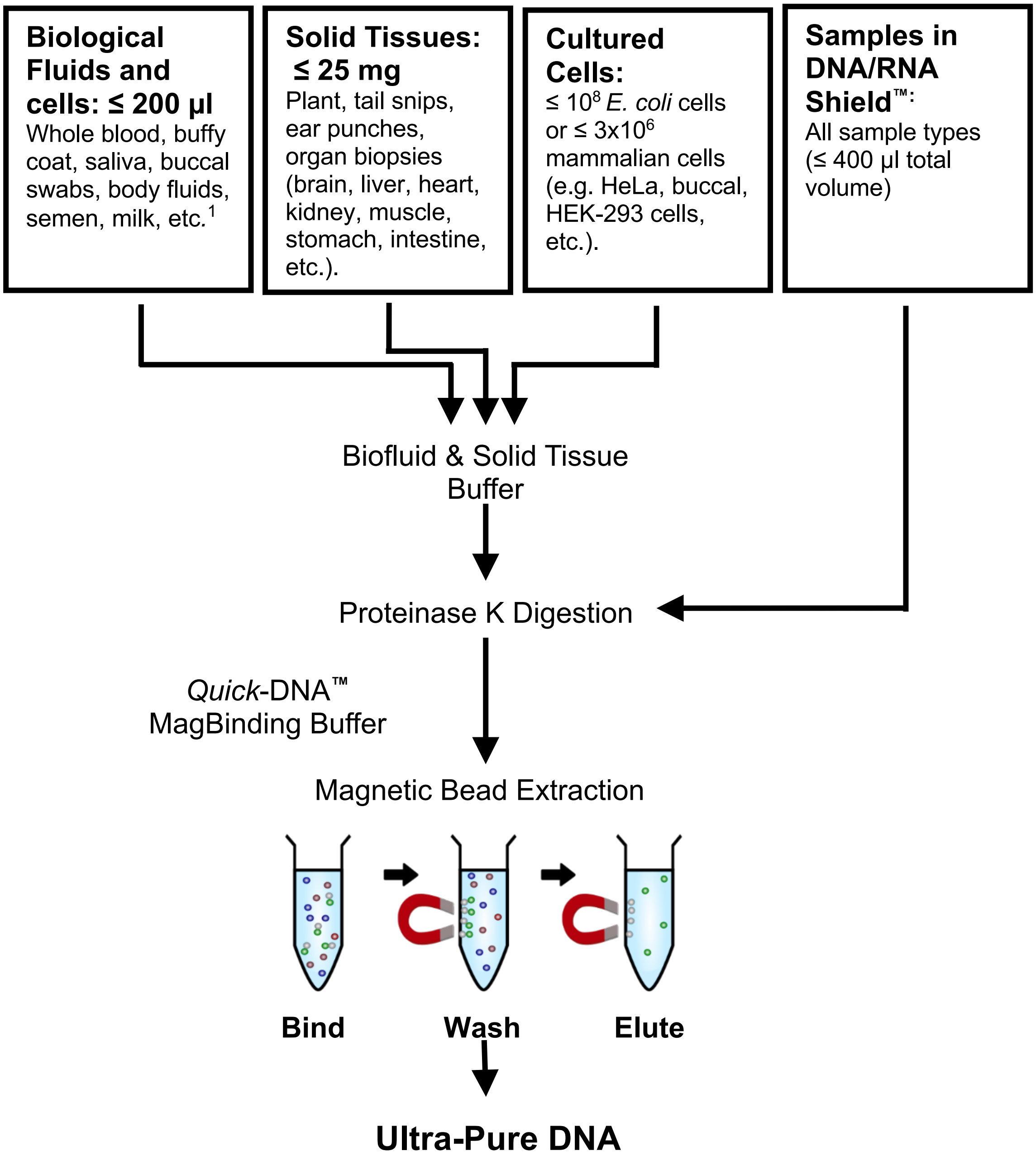

Notes:

[1] Viral DNA from serum or plasma samples can also be processed using this workflow. Not recommended for cell-free DNA isolation from urine, serum, or plasma samples.

Please refer to our **Quick-cfDNA™ Serum & Plasma Kit** (D4076) or **Quick-DNA™ Urine Kit** (D3061) for cell-free DNA isolation

Notes:

[1] If using < 200 µl sample, increase the volume to 200 µl using TE Buffer or an isotonic buffer before continuing.

[1] **DNA/RNA Shield**™ (R1100-50) is sold separately.

Reagent Preparation

✓ Add 1,040 µl **Proteinase K Storage Buffer** to each **Proteinase K** (20 mg) tube prior to use. The final concentration of **Proteinase K** is ~20 mg/ml. Store at -20ºC after mixing.

✓ Mix the **MagBinding Beads** until the beads are completely resuspended before use.

Protocol

The isolation consists of two steps: Sample Preparation & DNA Purification.

Sample Preparation

All steps should be performed at room temperature (20-30ºC) unless specified.

Wide bore tips can be used to prevent shearing of DNA.

Biological Fluids & Cells (Whole Blood, Saliva, *etc.*) ≤ 200 µl

1. Add 200 µl (equal volume) **Biofluid & Solid Tissue Buffer** to 200 µl liquid sample[1] and mix thoroughly.
2. Add 20 µl **Proteinase K** and pipette mix 5 times. Incubate at room temperature (20-30 °C) for 20 minutes.
3. Proceed to DNA Purification (Page 6).

Solid Tissue (Ear/Tail Snips, Liver, Plants, *etc.*) ≤ 25 mg

1. Add ≤ 25 mg solid tissue to 95 µl **DNA Elution Buffer**, 95 µl **Biofluid & Solid Tissue Buffer**, and 10 µl **Proteinase K**.
   Note: Plant tissue must be frozen with liquid nitrogen and ground into a fine powder before processing.
2. Pipette mix 5 times and incubate at 55ºC for 1-3 hours or until tissue solubilizes.
3. Centrifuge the sample at ≥ 10,000 x g with a microcentrifuge for 1 minute to pellet the debris.
4. Remove the supernatant while avoiding debris and transfer it to a new tube.
5. Proceed to DNA Purification (Page 6).

Samples in DNA/RNA Shield™ ≤ 400 µl or 25 mg

1. Add 20 µl **Proteinase K** to 400 µl sample in **DNA/RNA Shield**™[1] and mix well. Incubate at room temperature (20-30ºC) for 30 minutes.
   Note: Solid tissue in DNA/RNA Shield must be mechanically homogenized before processing.
2. Proceed to DNA Purification (Page 6).

Microbial/Environmental (Stool, Soil, Swabs) ≤ 100 mg or 10^8 Cells

1. Refer to Appendix A for enzymatic digestion of microbial samples (Page 8).

Phone: (949) 679-1190 ▪ Toll Free: (888) 882-9682 ▪ Fax: (949) 266-9452 ▪ info@zymoresearch.com ▪ www.zymoresearch.com

DNA Purification

1. Add 400 µl (equal volume) **Quick-DNA™ MagBinding Buffer** to 400 µl of the sample.
2. Pipette mix the solution.
3. Add the 33 µl of **MagBinding Beads**[1] to each sample.
4. Pipette mix 5 times and place the samples on a rotator or shaker[2] for 10 minutes.
5. Transfer the sample to the magnetic stand[3] until beads have separated from the solution, then remove[4] and discard the supernatant. Transfer the sample off the magnetic stand.
6. Add 500 µl ***Quick*-DNA™ MagBinding Buffer**.
7. Pipette mix to resuspend the beads (~5 times) and then place samples on a rotator or shaker for 5 minutes.
8. Transfer the sample to the magnetic stand until beads have separated from the solution, then remove[4] and discard the supernatant. Transfer the sample off the magnetic stand.
9. Add 500 µl **DNA Pre-Wash Buffer.**
10. Pipette mix to resuspend the beads (~10 times).
11. Transfer the sample to the magnetic stand until beads have separated from the solution, then remove[4] and discard the supernatant. Transfer the sample off the magnetic stand.
12. Add 900 µl **g-DNA Wash Buffer** and pipette mix to resuspend the beads (~10 times).
13. **Transfer all liquid into a new microcentrifuge tube or 96-well plate**.
14. Transfer the sample to the magnetic stand until beads have separated from the solution, and then remove[4] and discard the supernatant. Transfer the sample off the magnetic stand.
15. Repeat steps 12 – 14.
16. To dry the beads, transfer the sample to a heated element and incubate for 10 minutes. If no heating element is available, air dry for 20 minutes[5].
17. Add 50 µl of **DNA Elution Buffer** to each sample.
18. Pipette mix 20 times and incubate at room temperature for 5 minutes or mix via shaker for 5 minutes at room temperature (20-30ºC).
19. Transfer the sample to the magnetic stand until beads have separated from solution, then transfer the eluted DNA to a new tube (plate).

    The eluted DNA can be used immediately or stored at ≤-20ºC.

Notes:

[1] MagBinding Beads settle quickly, ensure that beads are kept in suspension while dispensing.

[2] Shaking speeds can be different for each shaker. Shaker should be fast enough to completely resuspending the beads (1100 – 1500 rpm).

[3] Magnetic stand (manual processing) or strong-field 96-well magnetic stand (*i.e.,* **ZR-96 MagStand**, P1005).

[4] Some beads will adhere to the sides of the well. When removing supernatant, aspirate slowly to allow these beads to be pulled to the magnet as the liquid level is lowered.

[5] Over drying the beads may result in lower DNA recovery. Beads will change in appearance from glossy black when still wet to a matte black/brown when fully dry.

Notes:

[1] **DNA/RNA Shield™ (2X Concentrate)** (R1200-25) is sold separately.

[2] **DNA/RNA Shield™** (R1100-50) is sold separately.

[2] **ZymoBIOMICS™ Microbial Community Standard** (D6300), **ZymoBIOMICS™ Microbial Community Standard II (Log Distribution)** (D6310), and **ZymoBIOMICS™ Spike-in Control I (High Microbial Load)** (D6320) are sold separately.

[3] Lysozyme (100 mg/mL) is available through Sigma-Aldrich (L2879-1G)

Appendix A: Ezymatic Digestion of Microbes

Enzymatic lysis of cells walls (e.g. Lysozyme, Zymolyase) from microbes is necessary to effectively isolate high molecular weight DNA from microbes.

Fluids (Whole Blood, Saliva, Water DNA/RNA Shield, Feces) ≤ 200 µl

1. Add 100 µl (equal volume) **DNA/RNA Shield™(2x Concentrate)**[1] to up to 100 µl sample and pipette mix 10 times.

   Note: If sample is already resuspended in DNA/RNA Shield™, add 100 µl **DNA/RNA Shield™**[2].

2. Incubate at room temperature (20-30°C) on a tube rotator or shaker for 5 minutes.

3. Proceed to Microbial Lysis.

Cells and Solids (Cultured Cells, Feces, Soil, etc.) ≤ 100 mg or 10^8 bacterial cells

1. Resuspend up to 100 mg of sample or up to 10^8 cells with 200 µl **DNA/RNA Shield™**[2] in a microcentrifuge tube and pipette mix well.

2. Incubate at room temperature (20-30°C) on a tube rotator for 5 minutes.

3. Proceed to Microbial Lysis.

Microbial Lysis

1. Centrifuge at 5,000 x g in a microcentrifuge for 1 minute to pellet the sample. Transfer the supernatant (~180 µl) in a **new** microcentrifuge tube. **Save both the supernatant and pellet.**

   Note: If a pellet does not appear after centrifugation, skip to step 9.

2. Add 100 µl PBS (user supplied) to sample pellet and pipette mix until pellet is visibly resuspended.

3. Centrifuge at 5,000 x g for 1 minute to pellet the sample. Combine the supernatant with the original sample supernatant (total ~280 µl) from the previous step.

4. Add 1 ml PBS (user supplied) to the new pellet and mix until pellet is visibly resuspended.

5. Centrifuge at 5,000 x g in a microcentrifuge for 1 minute to pellet the sample and discard the supernatant.

6. Add 100 µl TE Buffer and 25 µl lysozyme[3] (100 mg/ml; user supplied) to the pellet.

7. Pipette mix until pellet is visibly resuspended, then incubate at 55°C for 30 minutes.

8. Combine the saved supernatant (~280 µl) with the 125 µl digested sample.

9. Add 20 µl 10% SDS (user provided) and 10 µl **Proteinase K**. Briefly pipette mix and incubate at 55°C for 10 minutes.

10. Centrifuge 5,000 x g in a microcentrifuge for 1 minute to pellet residual debris. Transfer the supernatant to a new microcentrifuge tube.

11. Add 800 µl (2 volumes) **Quick-DNA™ MagBinding Buffer** to the sample and mix well

12. Proceed to step 3 of DNA Purification on Page 6.

Appendix B: Optional RNase Treatment

Notes:

This modification is necessary for downstream applications that are sensitive to trace amount of RNA. This step is not required for most long read sequencing applications (e.g., Nanopore, PacBio SMRT).

1. Continue after step 8 of the DNA Purification protocol on page 6.
2. Add 100 µl **DNA Elution Buffer** to the beads.
3. Pipette mix to resuspend the beads (~5 times).
4. Add 2 µl RNase A (1 mg/ml) to the sample.
5. Pipette mix to resuspend the beads (~5 times).
6. Incubate at room temperature (20 – 25 ºC) for 20 minutes.
7. Add 500 µl ***Quick*-DNA™ MagBinding Buffer**.
8. Pipette mix to resuspend the beads (~5 times) and then place samples on a rotator or shaker for 10 minutes.
9. Transfer the sample to the magnetic stand until beads have separated from the solution, then remove and discard the supernatant. Transfer the sample off the magnetic stand.
10. Continue to step 9 of the DNA Purification protocol on page 6.

Troubleshooting Guide

Problem	Possible Causes and Suggested Solutions
**Low DNA Yield or Quality**	
Binding Conditions	• **Binding Time.** Make sure to incubate on rotator or shaker for 10 minutes after the *Quick-DNA* MagBinding Buffer has been added to the sample. Incubation for longer periods of time may help to increase yield<br>• **Amount of MagBinding Beads.** The volume of beads used can be increased to 50 µl and eluted in 100 µl to increase the maximum binding capacity and accommodate samples of high biomass. 33 µl is the recommended starting point and can bind up to 10 µg (sample type dependent).
Proteinase K Digestion	• **Incubation Time.** The optimal time is largely sample dependent. 30 minutes is recommended for liquids whereas solid tissues may be incubated overnight for complete digestion. This will maximize yields but increases protocol time.
Magnetic Beads Preparation	• **Resuspension of Beads.** The MagBinding Beads settle quickly. Ensure complete resuspension before use by thoroughly shaking and/or vortexing the bottle.
Poor Mixing/Resuspension	• **Increase mixing cycles and/or speed.** Pipette mixing of the sample is crucial for some key steps (after adding Proteinase K and after adding MagBinding Beads) to ensure sufficient resuspension. Combined tip mixing and shaking at each step is recommended for optimal DNA yields and purities.
Drying Parameters	• **Prolonged Time or Increased Temperature.** Over-drying beads will result in severely reduced yields. To remove residual liquid, 55˚C for 10 minutes is a good starting point but can depend on specific plate dimensions and heater used.
Elution Parameters	• **Low Concentration.** If the final concentration of your extracted DNA is too low, use 15 µl MagBinding Beads and 30 µl DNA Elution Buffer when processing similar samples in future.<br>• **Incomplete Elution.** The recommended minimum elution volume is 1.5X ratio of the MagBinding Beads used (Ex. 50 µl beads to 75 µl elution). Using more volume ensures better surface coverage whereas using less volume can result in severely reduced yields and purities.<br>• **Temperature Conditions.** Incubating the elution step at 55˚C or greater during the minutes of shaking time may increase final yield.
**Low Purity**	
g-DNA Wash Conditions	• **New Tube Transfer.** It is crucial to transfer the g-DNA Wash / MagBead mixture to a new 1.5 mL microcentrifuge tube or 96-well plate during both wash steps. This prevents salt carryover which can lower purities.<br>• **Resuspension of Beads.** The MagBinding Beads settle out of solution quickly, so it is important to pre-mix the beads by pipette mixing to ensure full homogeneity before additional mixing via rotator or shaker.
Elution Washes	• **Insufficient Mixing.** It is important to properly mix the DNA Elution Buffer when added to the MagBinding Beads. Inefficient mixing can result in lower purities.
**Low Molecular Weight Size**	
Rigorous Mixing	• **Vortex and Shaking at High Speeds.** Mixing the sample using rigorous mixing parameters (e.g., vortex and shaking at high speeds) may cause shearing of the DNA, resulting in lower size recovery. Mixing via a rotator is recommended for higher size recovery.

Ordering Information

Product Description	Kit Size	Catalog No.
**Quick-DNA™ HMW MagBead Kit**	96 preps.	D6060

For Individual Sale	Amount	Catalog No.
**Proteinase K & Storage Buffer**	5 mg set<br>20 mg set	D3001-2-5<br>D3001-2-20
**Biofluid & Solid Tissue Buffer**	25 ml<br>100 ml	D4081-3-25<br>D4081-3-100
***Quick*-DNA™ MagBinding Buffer**	150 ml<br>250 ml	D4077-1-150<br>D4077-1-250
**DNA Pre-Wash Buffer**	15 ml<br>30 ml<br>50 ml<br>250 ml	D3004-5-15<br>D3004-5-30<br>D3004-5-50<br>D3004-5-250
**g-DNA Wash Buffer**	50 ml<br>100 ml<br>200 ml<br>250 ml<br>400 ml	D3004-2-50<br>D3004-2-100<br>D3004-2-200<br>D3004-2-250<br>D3004-2-400
**DNA Elution Buffer**	1 ml<br>4 ml<br>10 ml<br>16 ml<br>50 ml	D3004-4-1<br>D3004-4-4<br>D3004-4-10<br>D3004-4-16<br>D3004-4-50
**MagBinding Beads**	3 ml<br>8 ml<br>12 ml<br>16 ml<br>24 ml	D4100-4-3<br>D4100-4-8<br>D4100-4-12<br>D4100-4-16<br>D4100-4-24

ZYMO RESEARCH

The Beauty of Science is to Make Things Simple

ZYMO RESEARCH CORP.
Phone: (949) 679-1190 ▪ Toll Free: (888) 882-9682 ▪ Fax: (949) 266-9452 ▪ info@zymoresearch.com ▪ www.zymoresearch.com
