# Supplementary material for: Genomic DNA extraction optimization and validation for genome sequencing using the marine gastropod Kellet’s whelk
Source: PeerJ. 2023 Dec 6;11:e16510. doi: 10.7717/peerj.16510 (PMC10710129; doi:10.7717/peerj.16510)
Supplement: Supplemental Information 7 [file peerj-11-16510-s007.zip › Qiagen DNeasy Blood and Tissue Kit.pdf]

July 2020

DNeasy® Blood & Tissue Handbook

DNeasy Blood & Tissue Kit
DNeasy 96 Blood & Tissue Kit
For purification of total DNA from
animal blood
animal tissue
rodent tails
ear punches
cultured cells
fixed tissue
bacteria
insects

Sample to Insight

QIAGEN

Contents

Kit Contents 4

Storage 6

Intended Use 6

Safety Information 7

Quality Control 7

Introduction 8

- Principle and procedure 9
- Description of protocols 11
- Automated purification of DNA on QIAcube Instruments 12

Equipment and Reagents to Be Supplied by User 13

Important Notes 15

- Sample collection and storage 15
- Starting amounts of samples 15
- Maximum amount of starting material 15
- Very small sample sizes 16
- Quantification of starting material 17
- Preparation of Buffer AW1 and Buffer AW2 18
- Buffer AL 19
- Proteinase K 19
- Copurification of RNA 20
- Centrifugation (DNeasy 96 procedures) 21
- Elution of pure nucleic acids 23

Expected yields 24
Purification of high-molecular-weight DNA 25
Protocol: Purification of Total DNA from Animal Blood or Cells (Spin-Column Protocol) 26
Protocol: Purification of Total DNA from Animal Tissues (Spin-Column Protocol) 30
Protocol: Purification of Total DNA from Animal Blood or Cells (DNeasy 96 Protocol) 34
Protocol: Purification of Total DNA from Animal Tissues (DNeasy 96 Protocol) 39
Protocol: Pretreatment for Paraffin-Embedded Tissue 46
Protocol: Pretreatment for Formalin-Fixed Tissue 48
Protocol: Pretreatment for Gram-Negative Bacteria 49
Protocol: Pretreatment for Gram-Positive Bacteria 50
Troubleshooting Guide 52
Appendix A: Determination of Yield, Purity and Length of DNA 56
Appendix B: Cleaning S-Blocks 58
Ordering Information 59
Document Revision History 63

Kit Contents

DNeasy Blood & Tissue Kit<br>Catalog no.<br>Number of preps	(50)<br>69504<br>50	(250)<br>69506<br>250
DNeasy Mini Spin Columns (colorless) in 2 ml Collection Tubes	50	250
Collection Tubes (2 ml)	100	500
Buffer ATL	14 ml	50 ml
Buffer AL*	12 ml	2 x 33 ml
Buffer AW1 (concentrate)*†	19 ml	98 ml
Buffer AW2 (concentrate)†‡	13 ml	66 ml
Buffer AE	2 x 15 ml	2 x 60 ml
Proteinase K	1.25 ml	6 ml
Quick-Start Protocol	1	1

* Contains a chaotropic salt. Not compatible with disinfecting agents containing bleach. See page 7 for safety information.

† Buffer AW1 and Buffer AW2 are supplied as concentrates. Add ethanol (96–100%) according to the bottle label before use to obtain a working solution.

‡ Contains sodium azide as a preservative.

DNeasy 96 Blood & Tissue Kit<br>Catalog no.<br>Number of preps	(4)<br>69581<br>4 x 96	(12)<br>69582<br>12 x 96
DNeasy 96 Plates	4	12
S-Blocks*	2	2
Collection Microtubes, 1.2 ml (racked)	4 x 96	12 x 96
Collection Microtube Caps	2 x (120 x 8)	5 x (120 x 8)
Elution Microtubes RS (racked) and caps	4 x 96	12 x 96
AirPore Tape Sheets	25	3 x 25
Buffer AL†	86 ml	247 ml
Buffer ATL	80 ml	5 x 50 ml
Buffer AW1 (concentrate)†‡	98 ml	3 x 98 ml
Buffer AW2 (concentrate)‡§	81 ml	3 x 81 ml
Buffer AE	2 x 128 ml	500 ml
Proteinase K	2 x 7 ml	5 x 7 ml
96-Well-Plate Register	4	12
Quick-Start Protocol	1	1

* Reusable; see Appendix B (page 58) for cleaning instructions.

† Contains a chaotropic salt. Not compatible with disinfectants containing bleach. See page 7 for safety information.

‡ Buffer AW1 and Buffer AW2 are supplied as concentrates. Add ethanol (96–100%) according to the bottle label before use to obtain a working solution.

§ Contains sodium azide as a preservative.

Storage

DNeasy spin columns, DNeasy 96 plates and all buffers should be stored dry, at room temperature (15–25°C) and are stable for 1 year under these conditions, if not otherwise stated on the label.

DNeasy Blood & Tissue Kits contain a ready-to-use Proteinase K solution, which is supplied in a specially formulated storage buffer. Proteinase K is stable for at least 1 year after delivery when stored at room temperature. For storage longer than one year or if ambient temperatures often exceed 25°C, we suggest storing Proteinase K at 2–8°C.

Intended Use

DNeasy Blood & Tissue Kit and DNeasy 96 Blood & Tissue Kit are intended for molecular biology applications. This product is not intended for the diagnosis, prevention or treatment of a disease.

QIAcube® Connect is designed to perform fully automated purification of nucleic acids and proteins in molecular biology applications. The system is intended for use by professional users trained in molecular biological techniques and the operation of QIAcube Connect.

All due care and attention should be exercised in the handling of the products. We recommend all users of QIAGEN® products to adhere to the NIH guidelines that have been developed for recombinant DNA experiments or to other applicable guidelines.

Safety Information

When working with chemicals, always wear a suitable lab coat, disposable gloves and protective goggles. For more information, please consult the appropriate safety data sheets (SDSs). These are available online in convenient and compact PDF format at **www.qiagen.com/safety** where you can find, view and print the SDS for each QIAGEN kit and kit component.

**CAUTION**	DO NOT add bleach or acidic solutions directly to the sample preparation waste.

Buffers AL and AW1 contain guanidine salts, which can form highly reactive compounds when combined with bleach. If liquid containing these buffers is spilt, clean with a suitable laboratory detergent and water. If the spilt liquid contains potentially infectious agents, clean the affected area first with laboratory detergent and water, and then with 1% (v/v) sodium hypochlorite.

Quality Control

In accordance with QIAGEN's ISO-certified Quality Management System, each lot of the DNeasy Blood & Tissue Kit and DNeasy 96 Blood & Tissue Kit is tested against predetermined specifications to ensure consistent product quality.

Introduction

DNeasy Blood & Tissue Kits are designed for rapid purification of total DNA (e.g., genomic, mitochondrial and pathogen) from a variety of sample sources including fresh or frozen animal tissues and cells, blood or bacteria. DNeasy purified DNA is free of contaminants and enzyme inhibitors and is highly suited for PCR, Southern blotting, RAPD, AFLP and RFLP applications.

Purification requires no phenol or chloroform extraction or alcohol precipitation and involves minimal handling. This makes DNeasy Blood & Tissue Kits highly suited for simultaneous processing of multiple samples. For higher-throughput applications, the DNeasy 96 Blood & Tissue Kit enables simultaneous processing of 96 or 192 samples.

The buffer system is optimized to allow direct cell lysis followed by selective binding of DNA to the DNeasy membrane. After lysis, the DNeasy Blood & Tissue spin-column procedure can be completed in as little as 20 minutes. Using the DNeasy 96 Blood & Tissue Kit, 96 or 192 samples can be processed in just 1 hour after lysis.

Simple centrifugation processing completely removes contaminants and enzyme inhibitors, such as proteins and divalent cations, and allows simultaneous processing of multiple samples in parallel. In addition, DNeasy Blood & Tissue procedures are suitable for a wide range of sample sizes.

Purified DNA is eluted in low-salt buffer or water, ready for use in downstream applications. DNeasy purified DNA typically has an A_{260}/A_{280} ratio between 1.7 and 1.9 and is up to 50 kb in size, with fragments of 30 kb predominating. The DNeasy procedure also efficiently recovers DNA fragments as small as 100 bp.

Principle and procedure

DNeasy Blood & Tissue procedures are simple (see flowchart). Samples are first lysed using Proteinase K.* Buffering conditions are adjusted to provide optimal DNA-binding conditions and the lysate is loaded onto the DNeasy Mini spin column or the DNeasy 96 plate. During centrifugation, DNA is selectively bound to the DNeasy membrane as contaminants pass through. Remaining contaminants and enzyme inhibitors are removed in two efficient wash steps and DNA is then eluted in water or buffer, ready for use. DNeasy purified DNA has A_{260}/A_{280} ratios of 1.7–1.9, and absorbance scans show a symmetric peak at 260 nm confirming high purity.

The DNeasy membrane combines the binding properties of a silica-based membrane with simple microspin technology or with the QIAGEN 96-Well-Plate Centrifugation System. DNA adsorbs to the DNeasy membrane in the presence of high concentrations of chaotropic salt, which remove water from hydrated molecules in solution. Buffer conditions in DNeasy Blood & Tissue procedures are designed to enable specific adsorption of DNA to the silica membrane and optimal removal of contaminants and enzyme inhibitors.

* Lysis efficiency can be improved by cell disruption using a rotor–stator homogenizer, such as the TissueRuptor II, or a bead mill, such as the TissueLyser II. A supplementary protocol allowing the simultaneous disruption of up to 48 tissue samples using the TissueLyser II is available from QIAGEN Technical Services.

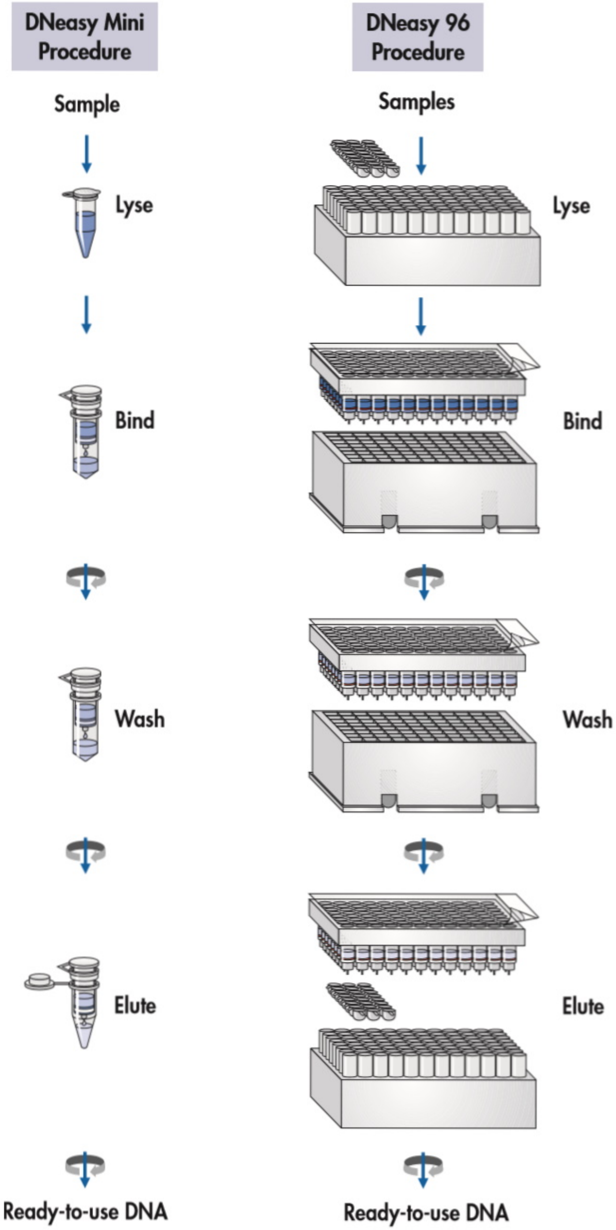
DNeasy Mini Procedure
Sample
Lyse
Bind
Wash
Elute
Ready-to-use DNA
DNeasy 96 Procedure
Samples
Lyse
Bind
Wash
Elute
Ready-to-use DNA

Description of protocols

Different protocols in this handbook provide detailed instructions to use DNeasy Kits for purification of total DNA.

- "Protocol: Purification of Total DNA from Animal Blood or Cells (Spin-Column Protocol)", page 26, is for use with the DNeasy Blood & Tissue Kit, for purification of DNA from animal blood (with nucleated or non-nucleated erythrocytes) or from cultured animal or human cells.
- "Protocol: Purification of Total DNA from Animal Tissues (Spin-Column Protocol)", page 30, is for use with the DNeasy Blood & Tissue Kit, for purification of DNA from animal tissues, including rodent tails.
- "Protocol: Purification of Total DNA from Animal Blood or Cells (DNeasy 96 Protocol)", page 34, is for use with the DNeasy 96 Blood & Tissue Kit, for high-throughput purification of DNA from animal blood (with nucleated or non-nucleated erythrocytes) or from cultured animal or human cells.
- "Protocol: Purification of Total DNA from Animal Blood or Cells (DNeasy 96 Protocol)", page 39, is for use with the DNeasy 96 Blood & Tissue Kit, for high-throughput purification of DNA from animal tissues, including rodent tails.

Pretreatment and specialized protocols

There are several pretreatment protocols included in this handbook, which are optimized for specific sample types. These pretreatment protocols are used in conjunction with one of the DNA purification protocols described above.

The following pretreatment protocols are included in this handbook.

- Pretreatment for Paraffin-Embedded Tissue, page 46
- Pretreatment for Formalin-Fixed Tissue, page 48
- Pretreatment for Gram-Negative Bacteria, page 49
- Pretreatment for Gram-Positive Bacteria, page 50

Additional optimized protocols for purification of DNA from yeast, hair, insects, crude lysates, bone, saliva and other specialized sample types are available online at **www.qiagen.com/shop/Sample-Technologies/DNeasy-Blood-and-Tissue-Kit** or from QIAGEN Technical Services (**support.qiagen.com**).

Automated purification of DNA on QIAcube Instruments

Purification of DNA can be fully automated on QIAcube Connect or the classic QIAcube. The innovative QIAcube instruments use advanced technology to process QIAGEN spin columns, enabling seamless integration of automated, low-throughput sample prep into your laboratory workflow. Sample preparation using QIAcube instruments follows the same steps as the manual procedure (i.e., lyse, bind, wash and elute), enabling you to continue using the DNeasy Blood & Tissue Kit for purification of high-quality DNA.

QIAcube instruments are preinstalled with protocols for purification of plasmid DNA, genomic DNA, RNA, viral nucleic acids and proteins, plus DNA and RNA cleanup. The range of protocols available is continually expanding, and additional QIAGEN protocols can be downloaded free of charge at **www.qiagen.com/qiacubeprotocols**.

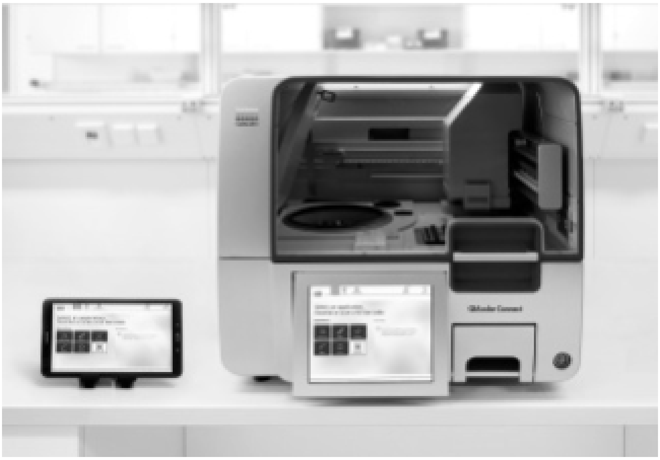

QIAcube Connect.

Equipment and Reagents to Be Supplied by User

When working with chemicals, always wear a suitable lab coat, disposable gloves and protective goggles. For more information, consult the appropriate material safety data sheets (SDSs), available from the product supplier.

For all protocols

- Pipettes and pipette tips
- Vortexer
- Ethanol (96–100%)*
- **Optional**: RNase A (100 mg/ml; cat. no. 19101)

For DNeasy Blood & Tissue Kit (spin column) protocols

- Microcentrifuge tubes (1.5 ml or 2 ml)
- Microcentrifuge with rotor for 1.5 ml and 2 ml tubes
- Thermomixer, shaking water bath or rocking platform for heating at 56°C

For DNeasy 96 Blood & Tissue Kit protocols

- Centrifuge 4-16S or 4-16KS with Plate Rotor 2 x 96 (see page 21)
- Multichannel pipette with extended tips

For efficient processing, we recommend the use of an electric multichannel pipette with a capacity of at least 1 ml per pipette tip. Options include the Matrix Impact® cordless electronic multichannel pipette, which has a unique, adjustable tip-spacing system allowing the user to transfer liquid directly from sample tubes to 96-well plates.

* Do not use denatured alcohol, which contains other substances, such as methanol or methylethylketone.

We recommend using extended tips with a maximum volume of 1250 µl with the Matrix multichannel pipette (available from Matrix, cat. no. 8255 for tips with filters or 8252 for tips without filters).

These multichannel pipettes and pipette tips can be purchased from Matrix Technologies Corporation **www.matrixtechcorp.com**).*

- Reagent reservoirs for multichannel pipettes
- Heavy plate or similar object to place on top of collection microtubes during incubation
- Oven or incubator for heating at 56°C

For blood and cultured cells

- PBS, pH 7.2 (50 mM potassium phosphate, 150 mM NaCl)

For pretreatment of paraffin-embedded tissue (page 46)

- Xylene

For pretreatment of formalin-fixed tissue (page 48)

- PBS, pH 7.2 (50 mM potassium phosphate, 150 mM NaCl)

For pretreatment of Gram-positive bacteria (page 50)

- Enzymatic lysis buffer:
  - 20 mM Tris·Cl, pH 8.0
  - 2 mM sodium EDTA
  - 1.2% Triton® X-100
  - Immediately before use, add lysozyme to 20 mg/ml

* This is not a complete list of suppliers and does not include many important vendors of biological supplies.

Important Notes

Sample collection and storage

Best results are obtained with fresh material or material that has been immediately frozen and stored at –90°C to –15°C. Repeated freezing and thawing of stored samples should be avoided, since this leads to reduced DNA size. Use of poor-quality starting material will also lead to reduced length and yield of purified DNA.

After Proteinase K digestion, tissue samples can also be stored in Buffer ATL for up to 6 months at ambient temperature without any reduction in DNA quality.

For certain bacterial cultures that accumulate large amounts of metabolites and/or form very dense cell walls, it is preferable to harvest cells in the early log phase of growth. Fresh or frozen cell pellets can be used in the procedure.

Starting amounts of samples

DNeasy Blood & Tissue procedures give DNA yields that increase linearly over a wide range, for both very small and large sample sizes (e.g., from as little as 100 cells up to 5 x 10^6 cells).

Maximum amount of starting material

To obtain optimum DNA yield and quality, it is important not to overload the DNeasy spin column or DNeasy 96 plate, as this can lead to significantly lower yields than expected (see Figure 1, page 16). For samples with very high DNA contents (e.g., spleen, which has a high cell density, and cell lines with a high degree of ploidy), less than the recommended amount of sample listed in Table 1 (page 16) should be used. If your starting material is not shown in Table 3 (page 24) and you have no information regarding DNA content, we recommend beginning with half the maximum amount of starting material indicated in Table 1. Depending on the yield obtained, the sample size can be increased in subsequent preparations.

Very small sample sizes

DNeasy Blood & Tissue procedures are also suitable for purifying DNA from very small amounts of starting material. If the sample has less than 5 ng DNA (<10,000 copies), 3–5 µg carrier DNA (a homopolymer, such as poly-dA, poly-dT or gDNA) should be added to the starting material. Ensure that the carrier DNA does not interfere with your downstream application. To prevent any interference of the carrier with the downstream application, an RNA carrier can be used. This can be removed later by RNase digestion. DNA or RNA homopolymers can be purchased from various suppliers.

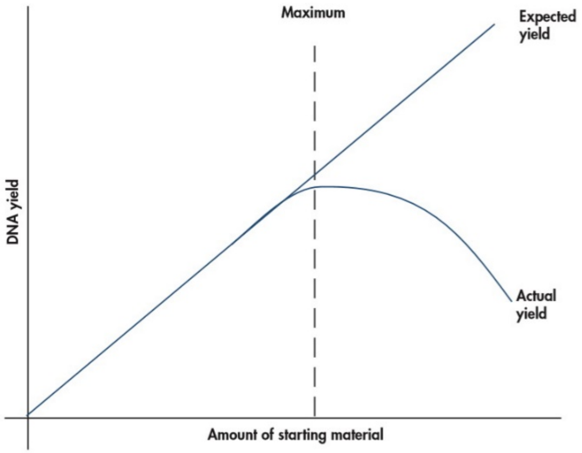

Figure 1. Schematic diagram of effect of sample size on DNA yield. If more than the maximum amount of starting material is used, DNA yield will be lower than expected.

Table 1. Maximum amounts of starting material

Sample	Amount
Animal tissue (see Table 3, page 24)	25 mg (spin-column protocols)<br>20 mg (DNeasy 96 protocols)
Mammalian blood (see Table 4, page 25)	100 µl
Bird or fish blood (with nucleated erythrocytes)	10 µl
Mouse tail	0.6–1.2 cm
Rat tail	0.6 cm
Cultured cells	5 x 10^6
Bacteria	2 x 10^9

Quantification of starting material

Weighing tissue or counting cells is the most accurate way to quantify starting material. However, the approximate guidelines given below can also be followed.

Animal tissue

A 2 mm cube (approximately this size: ■; volume, approximately 8 mm^3) of most animal tissues weighs approximately 10–15 mg.

Cells from cell culture

The number of HeLa cells obtained in various culture dishes after confluent growth is given in Table 2.

Table 2. Growth area and number of HeLa cells in various culture dishes

Cell culture vessel	Growth area* (cm^2)	Number of cells†
**Multiwell plates**		
96-well	0.32–0.6	4–5 x 10^4
48-well	1	1 x 10^5
24-well	2	2.5 x 10^5
12-well	4	5 x 10^5
6-well	9.5	1 x 10^6
**Dishes**		
35 mm	8	1 x 10^6
60 mm	21	2.5 x 10^6
100 mm	56	7 x 10^6
145–150 mm	145	2 x 10^7
**Flasks**		
40–50 ml	25	3 x 10^6
250–300 ml	75	1 x 10^7
650–750 ml	162–175	2 x 10^7

* Per well, if multiwell plates are used; varies slightly depending on the supplier.

† Cell numbers given are for HeLa cells (approximate length = 15 µm) assuming confluent growth. Cell numbers vary since animal cells can vary in length from 10 to 100 µm.

Bacteria

Bacterial growth is usually measured using a spectrophotometer. However, it is very difficult to give specific and reliable recommendations for the correlation between OD values and cell numbers in bacterial cultures. Cell density is influenced by a variety of factors (e.g., species, media and shaker speed) and OD readings of cultures measure light scattering rather than absorption. Measurements of light scattering are highly dependent on the distance between the sample and the detector and therefore readings vary between different types of spectrophotometer. In addition, different species show different OD values at defined wavelengths (e.g., 600 or 436 nm).

We therefore recommend calibrating the spectrophotometer used by comparing OD measurements at appropriate wavelengths with viable cell densities determined by plating experiments (e.g., see Ausubel, F.M. et al., eds. [1991] *Current Protocols in Molecular Biology*, New York: John Wiley & Sons, Inc.). OD readings should be between 0.05 and 0.3 to ensure significance. Samples with readings above 0.3 should be diluted so that the readings fall within this range and the dilution factor used in calculating the number of cells per milliliter.

The following calculation can be considered as a rough guide, which may be helpful. An *E. coli* culture of 1×10^9 cells per milliliter, diluted 1 in 4, gives OD_{600} values of 0.25 measured using a Beckman DU®-7400 or 0.125 using a Beckman DU-40 spectrophotometer. These correspond to calculated OD values of 1.0 or 0.5 respectively for 1×10^9 cells per milliliter.

Preparation of Buffer AW1 and Buffer AW2

Buffer AW1 and Buffer AW2 are supplied as concentrates. Before using for the first time, add the appropriate volume of ethanol (96–100%) as indicated on the bottle and shake thoroughly. Buffer AW1 and Buffer AW2 are stable for at least 1 year after the addition of ethanol when stored closed at room temperature (15–25°C).

Buffer AL

Purification of DNA from animal blood, cultured cells or Gram-positive bacteria

Buffer AL must be added to the sample and incubated at 56° C before ethanol is added. Ensure that ethanol has not been added to Buffer AL beforehand. Buffer AL can be purchased separately (see ordering information starting on page 59).

Purification of DNA from animal tissues

Buffer AL and ethanol (96–100%) are added in the same step. Buffer AL and ethanol can be premixed and added together in one step to save time when processing multiple samples.

For the protocol "Purification of Total DNA from Animal Tissues (DNeasy 96 Protocol)": Add 90 ml ethanol (96–100%) to the bottle containing 86 ml Buffer AL or 260 ml ethanol to the bottle containing 247 ml Buffer AL and shake thoroughly. Mark the bottle to indicate that ethanol has been added. (Please note that for purification of DNA from animal blood, Buffer AL must be used without ethanol. Buffer AL can be purchased separately if the same kit will be used for purification of DNA from animal blood.)

Buffer AL is stable for 1 year after the addition of ethanol when stored closed at room temperature (15–25°C).

Proteinase K

DNeasy Blood & Tissue Kits contain ready-to-use Proteinase K supplied in a specially formulated storage buffer. The activity of Proteinase K is 600 mAU/ml solution (or 40 mAU/mg protein) and has been chosen to provide optimal results.

Also included in the kits is an optimized buffer for tissue lysis, Buffer ATL. To enable efficient lysis, it is advisable to cut animal tissue into small pieces. If desired, lysis time can be reduced

to 20 minutes by grinding the sample in liquid nitrogen* before addition of Buffer ATL and Proteinase K. Alternatively, tissue samples can be effectively disrupted before Proteinase K digestion using a rotor–stator homogenizer, such as the TissueRuptor® II, or a bead mill, such as the TissueLyser II. A supplementary protocol for simultaneous disruption of up to 48 tissue samples using the TissueLyser II can be obtained by contacting QIAGEN Technical Services (**support.qiagen.com**).

Proteinase K is stable for at least one year after delivery when stored at room temperature (15–25°C). To store for more than one year or if ambient temperature often exceeds 25°C, we suggest keeping Proteinase K at 2–8°C. Please contact QIAGEN Technical Services or your local distributor if you have any questions about the use of Proteinase K (see back cover).

Copurification of RNA

DNeasy Blood & Tissue Kits copurify DNA and RNA when both are present in the sample. Transcriptionally active tissues, such as liver and kidney, contain high levels of RNA, which will be copurified. RNA may inhibit some downstream enzymatic reactions, although it does not affect PCR. If RNA-free genomic DNA is required, RNase A should be added to the sample before addition of Buffer AL, to digest the RNA. DNeasy protocols describe the use of an RNase A stock solution of 100 mg/ml. However, the amounts of RNase can be adjusted with less concentrated stock solutions, but not more than 20 µl of RNase solution should be used. Refer to the protocols for details.

* When working with chemicals, always wear a suitable lab coat, disposable gloves, and protective goggles. For more information, consult the appropriate safety data sheets (SDSs), available from the product supplier.

Centrifugation (DNeasy 96 procedures)

Centrifuges 4-16S and 4-16KS

DNeasy 96 spin protocols use a streamlined centrifugation procedure that enables purification of DNA from up to 2 x 96 samples in parallel for direct use in any downstream application. The DNeasy 96 Blood & Tissue procedure requires use of the QIAGEN 96-Well-Plate Centrifugation System, comprising the Plate Rotor 2 x 96 and the table-top Centrifuge 4-16S or the refrigerated table-top Centrifuge 4-16KS (see ordering information starting on page 59). In addition to the Plate Rotor 2 x 96, a wide range of other rotors can be used with these centrifuges.

Standard table-top centrifuges and microtiter plate rotors are not suitable for the DNeasy 96 protocol for 2 reasons: the microtiter plate buckets are either not deep enough for the complete DNeasy 96 package or they will not swing out properly, and, furthermore, high *g*-forces (>5500 x *g*) are required for optimal performance of the DNeasy 96 procedure. The speed limit of the Centrifuge 4-16S and the Centrifuge 4-16KS (6000 rpm; 5796 x *g*) is programmed so that the given *g*-force will not be exceeded. All centrifugation steps are performed at room temperature (15–25°C).

Important: Centrifuges must be properly maintained for optimal performance. It is particularly important that the buckets and rotor pins are routinely greased to prevent suboptimal running conditions that may lead to cracking of DNeasy 96 plates. For further information about QIAGEN Centrifuges and the Plate Rotor 2 x 96, contact QIAGEN Technical Services or your local distributor (see back cover for contact information).

Note: If the Centrifuge 4-16KS is used, set the temperature to 40° C for all centrifugation steps.

Note: Use AirPore Tape Sheets (provided) to seal DNeasy 96 plates during all centrifugation steps to prevent cross-contamination between samples.

Abbreviated instructions for using the Centrifuge 4-16S and Centrifuge 4-16KS

Warning: Never run the centrifuge with empty plate carriers placed inside the buckets, that is, without the collection microtubes or DNeasy 96 plates and S-Blocks. If unsupported, the carriers will collapse under high *g*-forces. Therefore, remove the carriers during test runs. Standard microtiter plates may be centrifuged in the same carriers if the *g*-force does not exceed 500 x *g*.

1. Switch on the centrifuge by pressing the main switch on the back.
2. Select the rotor selection list in the display field by turning the knob. After pressing the knob, turn the knob again to select the rotor/bucket combination **09100/09158** for the Plate Rotor 2 x 96. Confirm entry by pressing the knob. Entering the rotor number automatically sets the time and speed limits for centrifugation for that particular rotor, thus eliminating the danger of the centrifuge over-speeding.
3. Select **Speed** by turning the knob. Press the knob and by turning the knob again, set the speed to **6000**. Confirm entry by pressing the knob. The corresponding relative centrifugal force (RCF) is calculated from the rotor number and speed and appears automatically in the RCF field. It is also possible to enter the RCF value **5796 x *g*** manually in the RCF field after selecting **RCF** in the same way.
4. Select **Time** by turning the knob. Press once and by turning the knob again, set the time as recommended in the particular protocol step. Confirm entry by pressing the knob.
5. For the Centrifuge 4-16KS, set the temperature to 40°C.
6. Open the lid, place the 96-well plates with the metal carriers in the buckets then close the lid. The start and lid keys light up.
7. Push **Start** to start the centrifuge. When the centrifuge is running the lid key will not be lit. Each run can be interrupted by pushing **Stop**.
8. At the end of the run, the lid key will light up. Open the centrifuge lid by pressing the lid key. Remove the plates. All preset parameters remain after a run has finished.

Elution of pure nucleic acids

Purified DNA is eluted from the DNeasy Mini spin column or DNeasy 96 plate in either Buffer AE or water. For maximum DNA yield, elution is performed in two successive steps using 200 µl Buffer AE each. For more concentrated DNA, elution can be performed in two successive steps of 100 µl each. Keep in mind that elution volume and number of elution steps depends on the amount of DNA bound to the DNeasy membrane (see Figure 2).

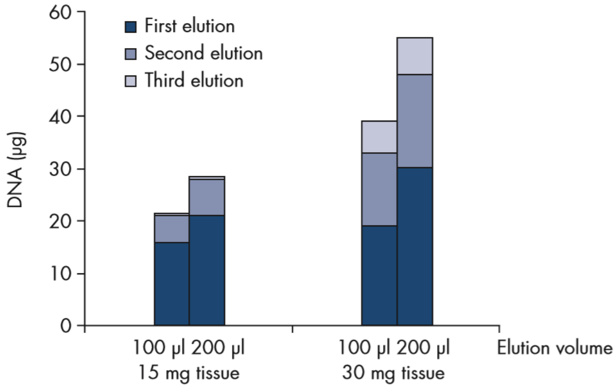

Figure 2. Yields of total nucleic acids in successive elutions of 100 or 200 µl.

For samples containing up to 10 µg DNA, a single elution step using 200 µl is sufficient. For samples containing more than 10 µg DNA, a second elution step with another 200 µl Buffer AE is recommended. Approximately 60–80% of the DNA will elute in the first elution. If >30 µg DNA is bound to the DNeasy membrane, elution in 3 x 200 µl may increase yield (Figure 2).

Elution in 100 µl increases the DNA concentration in the eluate, but reduces overall DNA yield. To prevent dilution of the first eluate, the subsequent elution step can be performed using a fresh 1.5 ml microcentrifuge tube. More than 200 µl should not be eluted into a 1.5 ml microcentrifuge tube because the spin column will come into contact with the eluate, leading to possible aerosol formation during centrifugation.

For very small samples (containing less than 1 µg DNA), only one elution in 50 µl of Buffer AE or water is recommended.

Buffer AE is 10 mM Tris·Cl, 0.5 mM EDTA, pH 9.0. Elution with Buffer AE guarantees optimal recovery and stability of eluted DNA. However, if you wish to elute DNA with water, please ensure that the pH of the water is at least 7.0 (deionized water from certain sources can be acidic). For long-term storage of DNA, elution in Buffer AE is strongly recommended since DNA stored in water is subject to acid hydrolysis. Buffer AE should be used at room temperature (15–25°C). Heating Buffer AE before elution is not necessary.

Expected yields

Yields of genomic DNA will vary from sample to sample depending on the amount and type of material processed. In addition, the quality of starting material will affect DNA yield.

Table 3 and Table 4 can be used to provide an estimate of expected yield.

Table 3. Typical DNA yields from animal tissues and cells

Source	Amount	DNA (µg)
Lymphocytes	5×10^6	15–25
HeLa cells	2×10^6	15–25
Liver	25 mg	10–30
Brain	25 mg	15–30
Lung	25 mg	5–10
Heart	25 mg	5–10
Kidney	25 mg	15–30
Spleen	10 mg	5–30
Mouse tail	1.2 cm (tip)	10–25
Rat tail	0.6 cm (tip)	20–40
Pig ear	25 mg	10–30
Horse hair	10 hairs	2–4
Fish fin	20 mg	10–20
Fish spawn (mackerel)	10 mg	5–10

Table 4. Typical DNA yields from animal blood

Animal	Amount (µl)	DNA (µg)
Cattle	100	4–5
Horse	100	3–5
Pig	100	3–6
Sheep	100	3–6
Dog	100	4–5
Cat	100	3–6
Goat	50*	3
Chicken†	5	9–15

* Using more than 50 µl goat blood gave no significant increase in DNA yield.
† Bird blood contains nucleated erythrocytes, giving higher DNA yields than mammalian blood.

Purification of high-molecular-weight DNA

QIAGEN Genomic-tips and Blood & Cell Culture DNA Kits are recommended for large-scale purification of high-molecular-weight DNA (see ordering information starting on page 59). QIAGEN Genomic-tips are available for purification of up to 500 µg of genomic DNA ranging in size from 50 to 150 kb. They are highly suited for use in Southern blotting, library construction, genome mapping and other demanding applications. QIAGEN also offers the MagAttract® HMW DNA Kit enables purification of high-molecular-weight (100–200 kb) DNA using a simple, magnetic bead-based protocol.

Please contact QIAGEN Technical Services at **support.qiagen.com** for more information.

Protocol: Purification of Total DNA from Animal Blood or Cells (Spin-Column Protocol)

This protocol is designed for purification of total DNA from animal blood (with nucleated or non-nucleated erythrocytes) or from cultured animal or human cells.

Important points before starting

- If using the DNeasy Blood & Tissue Kit for the first time, read "Important Notes" (page 15).
- All centrifugation steps are carried out at room temperature (15–25°C) in a microcentrifuge.
- Vortexing should be performed by pulse-vortexing for 5–10 s.
- PBS is required for use in step 1 (see page 14 for composition). Buffer ATL is not required in this protocol.
- **Optional**: RNase A may be used to digest RNA during the procedure. RNase A is not provided in the DNeasy Blood & Tissue Kit (see "Copurification of RNA", page 20).

Things to do before starting

- Buffer AL may form a precipitate upon storage. If necessary, warm to 56°C until the precipitate has fully dissolved.
- Buffer AW1 and Buffer AW2 are supplied as concentrates. Before using for the first time, add the appropriate amount of ethanol (96–100%) as indicated on the bottle to obtain a working solution.
- Preheat a thermomixer, shaking water bath or rocking platform to 56°C for use in step 2.

Procedure

1. For blood with non-nucleated erythrocytes, follow step 1a; for blood with nucleated erythrocytes, follow step 1b; for cultured cells, follow step 1c. Blood from mammals contains non-nucleated erythrocytes. Blood from animals, such as birds, fish or frogs, contains nucleated erythrocytes.

   1a. **Non-nucleated**: Pipet 20 µl Proteinase K into a 1.5 ml or 2 ml microcentrifuge tube (not provided). Add 50–100 µl anticoagulated blood. Adjust the volume to 220 µl with PBS. Continue with step 2.
   Optional: If RNA-free genomic DNA is required, add 4 µl RNase A (100 mg/ml) and incubate for 2 min at room temperature (15–25°C) before continuing with step 2.

   1b. **Nucleated**: Pipet 20 µl Proteinase K into a 1.5 ml or 2 ml microcentrifuge tube (not provided). Add 5–10 µl anticoagulated blood. Adjust the volume to 220 µl with PBS. Continue with step 2.
   Optional: If RNA-free genomic DNA is required, add 4 µl RNase A (100 mg/ml) and incubate for 2 min at room temperature before continuing with step 2.

   1c. **Cultured cells**: Centrifuge the appropriate number of cells (maximum 5 x 10^6) for 5 min at 300 x *g*. Resuspend the pellet in 200 µl PBS. Add 20 µl Proteinase K. Continue with step 2.
   When using a frozen cell pellet, allow cells to thaw before adding PBS until the pellet can be dislodged by gently flicking the tube.
   Ensure that an appropriate number of cells is used in the procedure. For cell lines with a high degree of ploidy (e.g., HeLa cells), it is recommended to use less than the maximum number of cells listed in Table 1 (page 16).
   Optional: If RNA-free genomic DNA is required, add 4 µl RNase A (100 mg/ml), mix by vortexing, and incubate for 2 min at room temperature before continuing with step 2.

2. Add 200 µl Buffer AL (without added ethanol). Mix thoroughly by vortexing, and incubate at 56°C for 10 min.

   Ensure that ethanol has not been added to Buffer AL (see "Buffer AL", page 19). Buffer AL can be purchased separately (see ordering information starting on page 59).

   It is essential that the sample and Buffer AL are mixed immediately and thoroughly by vortexing or pipetting to yield a homogeneous solution.

3. Add 200 µl ethanol (96–100%) to the sample, and mix thoroughly by vortexing.

   It is important that the sample and the ethanol are mixed thoroughly to yield a homogeneous solution.

4. Pipet the mixture from step 3 into the DNeasy Mini spin column placed in a 2 ml collection tube (provided). Centrifuge at ≥6000 x *g* (8000 rpm) for 1 min. Discard flow-through and collection tube.*

5. Place the DNeasy Mini spin column in a new 2 ml collection tube (provided), add 500 µl Buffer AW1, and centrifuge for 1 min at ≥6000 x *g* (8000 rpm). Discard flow-through and collection tube.*

6. Place the DNeasy Mini spin column in a new 2 ml collection tube (provided), add 500 µl Buffer AW2, and centrifuge for 3 min at 20,000 x *g* (14,000 rpm) to dry the DNeasy membrane. Discard flow-through and collection tube.

   It is important to dry the membrane of the DNeasy Mini spin column, since residual ethanol may interfere with subsequent reactions. This centrifugation step ensures that no residual ethanol will be carried over during the following elution.

   Following the centrifugation step, remove the DNeasy Mini spin column carefully so that the column does not come into contact with the flow-through, since this will result in carryover of ethanol. If carryover of ethanol occurs, empty the collection tube, then reuse it in another centrifugation for 1 min at 20,000 x *g* (14,000rpm).

* Flow-through contains Buffer AL or Buffer AW1 and is therefore not compatible with bleach. See page 6 for safety information

7. Place the DNeasy Mini spin column in a clean 1.5 ml or 2 ml microcentrifuge tube (not provided), and pipet 200 µl Buffer AE directly onto the DNeasy membrane. Incubate at room temperature for 1min, and then centrifuge for 1 min at ≥ 6000 x *g* (8000 rpm) to elute.

   Elution with 100 µl (instead of 200 µl) increases the final DNA concentration in the eluate, but also decreases the overall DNA yield (see Figure 2, page 23).

8. **Recommended**: For maximum DNA yield, repeat elution once as described in step 7.

   This step leads to increased overall DNA yield.

   A new microcentrifuge tube can be used for the second elution step to prevent dilution of the first eluate. Alternatively, to combine the eluates, the microcentrifuge tube from step 7 can be reused for the second elution step.

   Note: Do not elute more than 200 µl into a 1.5 ml microcentrifuge tube because the DNeasy Mini spin column will come into contact with the eluate.

Protocol: Purification of Total DNA from Animal Tissues (Spin-Column Protocol)

This protocol is designed for purification of total DNA from animal tissues, including rodent tails.

Important points before starting

- If using the DNeasy Blood & Tissue Kit for the first time, read "Important Notes" (page 15).
- For fixed tissues, refer to the pretreatment protocols "Pretreatment for Paraffin Embedded Tissue", page 46, and "Pretreatment for Formalin-Fixed Tissue", page 48.
- All centrifugation steps are carried out at room temperature (15–25°C) in a microcentrifuge.
- Vortexing should be performed by pulse-vortexing for 5–10 s.
- **Optional**: RNase A may be used to digest RNA during the procedure. RNase A is not provided in the DNeasy Blood & Tissue Kit (see "Copurification of RNA", page 20).

Things to do before starting

- Buffer ATL and Buffer AL may form precipitates upon storage. If necessary, warm to 56°C until the precipitates have fully dissolved.
- Buffer AW1 and Buffer AW2 are supplied as concentrates. Before using for the first time, add the appropriate amount of ethanol (96–100%) as indicated on the bottle to obtain a working solution.
- Preheat a thermomixer, shaking water bath or rocking platform to 56°C for use in step 2. If using frozen tissue, equilibrate the sample to room temperature (15–25°C).
- Avoid repeated thawing and freezing of samples, because this will lead to reduced DNA size.

Procedure

1. Cut up to 25 mg tissue (up to 10 mg spleen) into small pieces, and place in a 1.5 ml microcentrifuge tube. For rodent tails, place one (rat) or two (mouse) 0.4–0.6 cm lengths of tail into a 1.5 ml microcentrifuge tube. Add 180 µl Buffer ATL. Earmark the animal appropriately.

   Ensure that the correct amount of starting material is used (see "Starting amounts of samples", page 15). For tissues, such as spleen, with a very high number of cells for a given mass of tissue, no more than 10 mg starting material should be used.

   We strongly recommend cutting the tissue into small pieces to enable more efficient lysis. If desired, lysis time can be reduced by grinding the sample in liquid nitrogen* before addition of Buffer ATL and Proteinase K. Alternatively, tissue samples can be effectively disrupted before Proteinase K digestion using a rotor–stator homogenizer, such as the TissueRuptor II, or a bead mill, such as the TissueLyser II (see ordering information starting on page 59). A supplementary protocol for simultaneous disruption of up to 48 tissue samples using the TissueLyser II can be obtained by contacting QIAGEN Technical Services (see back cover). For rodent tails, a maximum of 1.2 cm (mouse) or 0.6 cm (rat) tail should be used. When purifying DNA from the tail of an adult mouse or rat, it is recommended to use only 0.4–0.6 cm.

2. Add 20 µl Proteinase K. Mix thoroughly by vortexing, and incubate at 56°C until the tissue is completely lysed. Vortex occasionally during incubation to disperse the sample or place in a thermomixer, shaking water bath or on a rocking platform.

   Lysis time varies depending on the type of tissue processed. Lysis is usually complete in 1–3 h or, for rodent tails, 6–8 h. If it is more convenient, samples can be lysed overnight; this will not affect them adversely.

   After incubation the lysate may appear viscous, but should not be gelatinous as it may clog the DNeasy Mini spin column. If the lysate appears very gelatinous, see the "Troubleshooting Guide", page 52, for recommendations.

* When working with chemicals, always wear a suitable lab coat, disposable gloves, and protective goggles. For more information, consult the appropriate safety data sheets (SDSs), available from the product supplier.

Optional: If RNA-free genomic DNA is required, add 4 µl RNase A (100 mg/ml), mix by vortexing, and incubate for 2 min at room temperature (15–25°C) before continuing with step 3.

Transcriptionally active tissues, such as liver and kidney, contain high levels of RNA, which will copurify with genomic DNA. For tissues that contain low levels of RNA, such as rodent tails, or, if residual RNA is not a concern, RNase A digestion is not necessary.

3. Vortex for 15 s. Add 200 µl Buffer AL to the sample, and mix thoroughly by vortexing. Then add 200 µl ethanol (96–100%), and mix again thoroughly by vortexing.

   It is essential that the sample, Buffer AL, and ethanol are mixed immediately and thoroughly by vortexing or pipetting to yield a homogeneous solution. Buffer AL and ethanol can be premixed and added together in one step to save time when processing multiple samples.

   A white precipitate may form on addition of Buffer AL and ethanol. This precipitate does not interfere with the DNeasy procedure. Some tissue types (e.g., spleen, lung) may form a gelatinous lysate after addition of Buffer AL and ethanol. In this case, vigorously shaking or vortexing the preparation is recommended.

4. Pipet the mixture from step 3 (including any precipitate) into the DNeasy Mini spin column placed in a 2 ml collection tube (provided). Centrifuge at ≥6000 x *g* (8000 rpm) for 1 min. Discard flow-through and collection tube.*

5. Place the DNeasy Mini spin column in a new 2 ml collection tube (provided), add 500 µl Buffer AW1, and centrifuge for 1 min at ≥6000 x *g* (8000 rpm). Discard flow-through and collection tube.*

* Flow-through contains Buffer AL or Buffer AW1 and is therefore not compatible with bleach. See page 6 for safety information.

6. Place the DNeasy Mini spin column in a new 2 ml collection tube (provided), add 500 µl Buffer AW2, and centrifuge for 3 min at 20,000 x *g* (14,000 rpm) to dry the DNeasy membrane. Discard flow-through and collection tube.

   It is important to dry the membrane of the DNeasy Mini spin column, since residual ethanol may interfere with subsequent reactions. This centrifugation step ensures that no residual ethanol will be carried over during the following elution.

   Following the centrifugation step, remove the DNeasy Mini spin column carefully so that the column does not come into contact with the flow-through, since this will result in carryover of ethanol. If carryover of ethanol occurs, empty the collection tube, then reuse it in another centrifugation for 1 min at 20,000 x *g* (14,000rpm).

7. Place the DNeasy Mini spin column in a clean 1.5 ml or 2 ml microcentrifuge tube (not provided), and pipet 200 µl Buffer AE directly onto the DNeasy membrane. Incubate at room temperature for 1 min, and then centrifuge for 1 min at ≥ 6000 x *g* (8000 rpm) to elute.

   Elution with 100 µl (instead of 200 µl) increases the final DNA concentration in the eluate, but also decreases the overall DNA yield (see Figure 2, page 23).

8. **Recommended**: For maximum DNA yield, repeat elution once as described in step 7.

   This step leads to increased overall DNA yield.

   A new microcentrifuge tube can be used for the second elution step to prevent dilution of the first eluate. Alternatively, to combine the eluates, the microcentrifuge tube from step 7 can be reused for the second elution step.

   Note: Do not elute more than 200 µl into a 1.5 ml microcentrifuge tube because the DNeasy Mini spin column will come into contact with the eluate.

Protocol: Purification of Total DNA from Animal Blood or Cells (DNeasy 96 Protocol)

This protocol is designed for high-throughput purification of total DNA from animal blood (with nucleated or non-nucleated erythrocytes) or from cultured animal or human cells

Important points before starting

- If using the DNeasy 96 Blood & Tissue Kit for the first time, read "Important Notes" (page 15).
- All centrifugation steps are carried out at room temperature (15–25°C).
- PBS is required for use in step 1 (see page 14 for composition). Buffer ATL is not required in this protocol.
- Ensure that ethanol has not been added to Buffer AL (see "Important Notes", page 15). Buffer AL can be purchased separately (see ordering information starting on page 59).
- **Optional**: RNase A may be used to digest RNA during the procedure. RNase A is not provided in the DNeasy 96 Blood & Tissue Kit (see "Copurification of RNA", page 20).

Things to do before starting

- Buffer ATL and Buffer AL may form precipitates upon storage. If necessary, warm to 56°C for 5 min until the precipitates have fully dissolved.
- Buffer AW1 and Buffer AW2 are supplied as concentrates. Before using for the first time, add the appropriate amount of ethanol (96–100%) as indicated on the bottle to obtain a working solution.
- Mix Buffer AW1 before use by inverting several times.
- Preheat an incubator to 56°C for use in step 2.

Procedure

1. For blood with non-nucleated erythrocytes, follow step1a; for blood with nucleated erythrocytes, follow step 1b; for cultured cells, follow step 1c.

   Blood from mammals contains non-nucleated erythrocytes. Blood from animals, such as birds, fish or frogs, contains nucleated erythrocytes.

   1a. **Non-nucleated**: Pipet 20 µl Proteinase K into each collection microtube. Add 50–100 µl anticoagulated blood per collection microtube. Use a 96-Well-Plate Register (provided) to identify the position of each sample. Adjust the volume to 220 µl each with PBS. Continue with step 2.
   Optional: If RNA-free genomic DNA is required, add 4 µl RNase A (100 mg/ml) and incubate for 5 min at room temperature (15–25°C) before continuing with step 2.

   Keep the clear covers from the collection microtube racks for use in step 3.

   1b. **Nucleated**: Pipet 20 µl Proteinase K into each collection microtube. Add 5–10 µl anticoagulated blood. Use a 96-Well-Plate Register (provided) to identify the position of each sample. Adjust the volume to 220 µl each with PBS. Continue with step 2.
   Optional: If RNA-free genomic DNA is required, add 4 µl RNase A (100 mg/ml) and incubate for 5 min at room temperature before continuing with step 2.

   Keep the clear covers from the collection microtube racks for use in step 3.

   1c. **Cultured cells**: Centrifuge the appropriate number of cells (maximum 5 x 10^6 each) for 5 min at 300 x *g*. Use a 96-Well-Plate Register (provided) to identify the position of each sample. Resuspend the pellets in 200 µl PBS each. Add 20 µl Proteinase K each. Continue with step 2.
   When using a frozen cell pellet, allow cells to thaw before adding PBS until the pellet can be dislodged by gently flicking the tube.

   Ensure that an appropriate number of cells is used in the procedure. For cell lines with a high degree of ploidy (e.g., HeLa cells), it is recommended to use less than the maximum number of cells listed in Table 1 (page 16).

Optional: If RNA-free genomic DNA is required, add 4 µl RNase A (100 mg/ml). Seal the collection microtubes properly using the caps provided, mix by vortexing, and incubate for 5 min at room temperature before continuing with step 2.

Keep the clear covers from the collection microtube racks for use in step 3.

2. Add 200 µl Buffer AL (without added ethanol).

   Ensure that ethanol has not been added to Buffer AL (see "Buffer AL", page 19).

   Buffer AL can be purchased separately (see page 59 for ordering information).

3. Seal the collection microtubes properly using the caps provided.

   Place a clear cover (saved from step 1) over each rack of collection microtubes, and shake the racks vigorously up and down for 15 s. To collect any solution from the caps, centrifuge the collection microtubes. Allow the centrifuge to reach 3000 rpm, and then stop the centrifuge.

   Do not prolong this step.

   Important: The rack of collection microtubes must be vigorously shaken up and down with both hands to obtain a homogeneous lysate. Inverting the rack of collection microtubes is not sufficient for mixing. The genomic DNA will not be sheared by vigorous shaking. The lysate and Buffer AL should be mixed immediately and thoroughly to yield a homogeneous solution.

   Keep the clear covers from the collection microtube racks for use in step 6.

4. Incubate at 56°C for 10 min. Place a weight on top of the caps during the incubation. Mix occasionally during incubation to disperse the sample, or place on a rocking platform.

   Note: Do not use a rotary- or vertical-type shaker as continuous rotation may release the caps. If incubation is performed in a water bath make sure that the collection microtubes are not fully submerged and that any remaining water is removed prior to removing the caps in step 5.

5. Carefully remove the caps, and add 200 µl ethanol (96–100%) to each sample.

6. Seal the collection microtubes properly using the caps provided. Place a clear cover over each rack of collection microtubes, and shake the racks vigorously up and down for 15 s. To collect any solution from the caps, centrifuge the collection microtubes. Allow the centrifuge to reach 3000 rpm, and then stop the centrifuge.

   Do not prolong this step.

   Important: The rack of collection microtubes must be vigorously shaken up and down with both hands to obtain a homogeneous lysate. Inverting the rack of collection microtubes is not sufficient for mixing. The genomic DNA will not be sheared by vigorous shaking. The lysate and ethanol should be mixed immediately and thoroughly to yield a homogeneous solution.

7. Place two DNeasy 96 plates on top of S-Blocks (provided). Mark the DNeasy 96 plates for later sample identification.

8. Remove and discard the caps from the collection microtubes. Carefully transfer the lysis mixture (maximum 900 µl) of each sample from step 6 to each well of the DNeasy 96 plates.

   Take care not to wet the rims of the wells to avoid aerosols during centrifugation. Do not transfer more than 900 µl per well.

   Note: Lowering pipette tips to the bottoms of the wells may cause sample overflow and cross-contamination. Therefore, remove one set of caps at a time, and begin drawing up the samples as soon as the pipette tips contact the liquid. Repeat until all the samples have been transferred to the DNeasy 96 plates.

9. Seal each DNeasy 96 plate with an AirPore Tape Sheet (provided). Centrifuge for 4 min at 6000 rpm.

   AirPore Tape prevents cross-contamination between samples during centrifugation.

   After centrifugation, check that all of the lysate has passed through the membrane in each well of the DNeasy 96 plates. If lysate remains in any of the wells, centrifuge for a further 4 min.

10. Remove the tape. Carefully add 500 µl Buffer AW1 to each sample.

    Note: Ensure that ethanol has been added to Buffer AW1 prior to use.

11. Seal each DNeasy 96 plate with a new AirPore Tape Sheet (provided). Centrifuge for 2 min at 6000 rpm.
12. Remove the tape. Carefully add 500 µl Buffer AW2 to each sample.

    Note: Ensure that ethanol has been added to Buffer AW2 prior to use.
13. Centrifuge for 15 min at 6000 rpm.

    Do not seal the plate with AirPore Tape.

    The heat generated during centrifugation ensures evaporation of residual ethanol in the sample (from Buffer AW2) that might otherwise inhibit downstream reactions.
14. Place each DNeasy 96 plate in the correct orientation on a new rack of Elution Microtubes RS (provided).
15. To elute the DNA, add 200 µl Buffer AE to each sample, and seal the DNeasy 96 plates with new AirPore Tape Sheets (provided). Incubate for 1 min at room temperature. Centrifuge for 4 min at 6000 rpm.

    Two hundred microliters Buffer AE is sufficient to elute up to 75% of the DNA from each well of the DNeasy 96 plate.

    Elution with volumes less than 200 µl significantly increases the final DNA concentration of the eluate but may reduce overall DNA yield. For samples containing less than 1 µg DNA, elution in 50 µl Buffer AE is recommended.
16. **Recommended**: For maximum DNA yield, repeat step 15 with another 200 µl Buffer AE.

    A second elution with 200 µl Buffer AE will increase the total DNA yield by up to 25%. However due to the increased volume, the DNA concentration is reduced. If a higher DNA concentration is desired, the second elution step can be performed using the 200 µl eluate from the first elution. This will increase the yield by up to 15%.

    Use new caps (provided) to seal the Elution Microtubes RS for storage.

Protocol: Purification of Total DNA from Animal Tissues (DNeasy 96 Protocol)

This protocol is designed for high-throughput purification of total DNA from animal tissues, including rodent tails.

Important points before starting

- If using the DNeasy 96 Blood & Tissue Kit for the first time, read "Important Notes" (page 15).
- All centrifugation steps are carried out at room temperature (15–25°C).
- **Optional**: RNase A may be used to digest RNA during the procedure. RNase A is not provided in the DNeasy 96 Blood & Tissue Kit (see "Copurification of RNA", page 20).

Things to do before starting

- Buffer AL should be premixed with ethanol before use. Add 90 ml ethanol (96–100%) to the bottle containing 86 ml Buffer AL or 260 ml ethanol to the bottle containing 247 ml Buffer AL and shake thoroughly. Mark the bottle to indicate that ethanol has been added. (Please note that, for purification of DNA from animal blood, Buffer AL must be used without ethanol. Buffer AL can be purchased separately if the same kit will be used for purification of DNA from animal blood.)
- Buffer AW1 and Buffer AW2 are supplied as concentrates. Before using for the first time, add the appropriate amount of ethanol (96–100%) as indicated on the bottle to obtain a working solution.
- Buffer ATL and Buffer AL may form precipitates upon storage. If necessary, warm to 56°C for 5 min until the precipitates have fully dissolved.

- Mix Buffer AW1 before use by inverting several times.
- Preheat an incubator to 56°C for use in step 4.
- If using frozen tissue, equilibrate the sample to room temperature (15–25°C). Avoid repeated thawing and freezing of samples since this will lead to reduced DNA size.

Procedure

1. Cut up to 20 mg tissue (up to10 mg spleen) into small pieces. For rodent tails, place one (rat) or two (mouse) 0.4–0.6 cm lengths of tail into a collection microtube. Earmark the animal appropriately. Use a 96-Well-Plate Register (provided) to identify the position of each sample.

   Ensure that the correct amount of starting material is used (see "Starting amounts of samples", page 15). For tissues, such as spleen, with a very high number of cells for a given mass of tissue, no more than 10 mg starting material should be used.

   We strongly recommend cutting the tissue into small pieces to enable more efficient lysis. If desired, lysis time can be reduced by disrupting the sample using a bead mill, such as the TissueLyser II (see ordering information starting on page 59), before addition of Buffer ATL and Proteinase K. A supplementary protocol for simultaneous disruption of up to 48 tissue samples using the TissueLyser II can be obtained by contacting QIAGEN Technical Services at **support.qiagen.com**.

   For rodent tails, a maximum of 1.2 cm (mouse) or 0.6 cm (rat) tail should be used. When purifying DNA from the tail of an adult mouse or rat, it is recommended to use only 0.4–0.6 cm.

   Store the samples at –30°C to –15°C until a suitable number has been collected (up to 192 samples). Samples can be stored at –30°C to –15°C for several weeks to months without any reduction in DNA yield. DNA yields will be approximately 10–30 µg, depending on the type, length, age and species of sample used (see "Expected yields", page 24).

   Keep the clear covers from the collection microtube racks for use in step 3.

2. Prepare a Proteinase K–Buffer ATL working solution containing 20µl Proteinase K stock solution and 180 µl Buffer ATL per sample, and mix by vortexing. For one set of 96 samples, use 2 ml Proteinase K stock solution and 18 ml Buffer ATL. Immediately pipet 200 µl working solution into each collection microtube containing the tail sections or tissue samples. Seal the microtubes properly using the caps provided.

   Note: Check Buffer ATL for precipitate. If necessary, dissolve the precipitate by incubation at 56°C for 5 min before preparing the working solution.

   Important: After preparation, the Proteinase K–Buffer ATL working solution should be dispensed immediately into the collection microtubes containing the tail or tissue samples. Incubation of the working solution in the absence of substrate for >30 min reduces lysis efficiency and DNA purity.

3. Ensure that the microtubes are properly sealed to avoid leakage during shaking. Place a clear cover (saved from step 1) over each rack of collection microtubes, and mix by inverting the rack of collection microtubes. To collect any solution from the caps, centrifuge the collection microtubes. Allow the centrifuge to reach 3000 rpm, and then stop the centrifuge. It is essential that the samples are completely submerged in the Proteinase K–Buffer ATL working solution after centrifugation.

   If the Proteinase K–Buffer ATL working solution does not completely cover the sample, increase the volume of the solution to 300 µl per sample (additional reagents are available separately; see ordering information starting on page 59). Do not increase volumes above 300 µl as this will exceed the capacity of the collection microtubes in subsequent steps.

   Keep the clear covers from the collection microtube racks for use in step 5.

4. Incubate at 56°C overnight or until the samples are completely lysed. Place a weight on top of the caps during the incubation. Mix occasionally during incubation to disperse the sample, or place on a rocking platform.

   Lysis time varies depending on the type, age and amount of tail or tissue being processed. Lysis is usually complete in 1–3 h or, for rodent tails, 6–8h, but optimal results will be achieved after overnight lysis.

   After incubation the lysate may appear viscous, but should not be gelatinous as it may clog the DNeasy 96 membrane. If the lysate appears very gelatinous, see the "Troubleshooting Guide", page 52, for recommendations.

   Note: Do not use a rotary- or vertical-type shaker as continuous rotation may release the caps. If incubation is performed in a water bath make sure that the collection microtubes are not fully submerged and that any remaining water is removed prior to centrifugation in step 5.

5. Ensure that the microtubes are properly sealed to avoid leakage during shaking. Place a clear cover over each rack of collection microtubes and shake the racks vigorously up and down for 15 s. To collect any solution from the caps, centrifuge the collection microtubes. Allow the centrifuge to reach 3000 rpm, and then stop the centrifuge.

   Important: The rack of collection microtubes must be vigorously shaken up and down with both hands to obtain a homogeneous lysate. Inverting the rack of collection microtubes is not sufficient for mixing. The genomic DNA will not be sheared by vigorous shaking.

   Keep the clear covers from the collection microtube racks for use in step 7.

   Ensure that lysis is complete before proceeding to step 6. The lysate should be homogeneous following the vigorous shaking. To check this, slowly invert the rack of collection microtubes (making sure that the caps are tightly closed) and look for a gelatinous mass. If a gelatinous mass is visible, lysis needs to be extended by adding another 100 µl Buffer ATL and 15 µl Proteinase K, and incubating for a further 3 h. It is very important to ensure that samples are completely lysed to achieve optimal yields and to avoid clogging of individual wells of the DNeasy 96 plate.

Optional: If RNA-free genomic DNA is required, add 4 µl RNase A (100 mg/ml). Close the collection microtubes with fresh caps, mix by shaking vigorously, and incubate for 5 min at room temperature (15–25°C). To collect any solution from the caps, centrifuge the collection microtubes. Allow the centrifuge to reach 3000 rpm, and then stop the centrifuge. Remove the caps, and continue with step 6. Transcriptionally active tissues, such as liver and kidney, contain high levels of RNA, which will copurify with genomic DNA. For tissues that contain low levels of RNA, such as rodent tails, or, if residual RNA is not a concern, RNase A digestion is usually not necessary.

6. Carefully remove the caps. Add 410 µl premixed Buffer AL–ethanol to each sample.

   Note: Ensure that ethanol has been added to Buffer AL prior to use (see "Buffer AL", page 19).

   Note: A white precipitate may form upon addition of Buffer AL–ethanol to the lysate. It is important to apply all of the lysate, including the precipitate, to the DNeasy 96 plate in step 9. This precipitate does not interfere with the DNeasy procedure or with any subsequent application.

   If the volumes of Buffer ATL and Proteinase K were increased in steps 3 or 5, increase the volume of Buffer AL and ethanol accordingly. For example, 300 µl Proteinase K–Buffer ATL working solution will require 615 µl Buffer AL–ethanol.

7. Ensure that the microtubes are properly sealed to avoid leakage during shaking. Place a clear cover over each rack of collection microtubes and shake the racks vigorously up and down for 15 s. To collect any solution from the caps, centrifuge the collection microtubes. Allow the centrifuge to reach 3000 rpm, and then stop the centrifuge.

   Do not prolong this step.

   Important: The rack of collection microtubes must be vigorously shaken up and down with both hands to obtain a homogeneous lysate. Inverting the rack of collection microtubes is not sufficient for mixing. The genomic DNA will not be sheared by vigorous shaking. The lysate and Buffer AL–ethanol should be mixed immediately and thoroughly to yield a homogeneous solution.

8. Place two DNeasy 96 plates on top of S-Blocks (provided). Mark the DNeasy 96 plates for later sample identification.
9. Remove and discard the caps from the collection microtubes. Carefully transfer the lysate (maximum 900 µl) of each sample from step 7 to each well of the DNeasy 96 plates.

   Take care not to wet the rims of the wells to avoid aerosols during centrifugation. Do not transfer more than 900 µl per well.

   Note: Lowering pipette tips to the bottoms of the wells may cause sample overflow and cross-contamination. Therefore, remove one set of caps at a time, and begin drawing up the samples as soon as the pipette tips contact the liquid. Repeat until all the samples have been transferred to the DNeasy 96 plates.

   Note: If the volume of Proteinase K–Buffer ATL working solution was increased in steps 3 or 5, transfer no more than 900 µl of the supernatant from step 7 to the DNeasy 96 plate. Larger amounts will exceed the volume capacity of the individual wells. Discard any remaining supernatant from step 7 as this will not contribute significantly to the total DNA yield.
10. Seal each DNeasy 96 plate with an AirPore Tape Sheet (provided). Centrifuge for 10 min at 6000 rpm.

    AirPore Tape prevents cross-contamination between samples during centrifugation.

    After centrifugation, check that all of the lysate has passed through the membrane in each well of the DNeasy 96 plates. If lysate remains in any of the wells, centrifuge for a further 10 min.
11. Remove the tape. Carefully add 500 µl Buffer AW1 to each sample.

    Note: Ensure that ethanol has been added to Buffer AW1 prior to use. It is not necessary to increase the volume of Buffer AW1 if the volume of Proteinase K–Buffer ATL working solution was increased in steps 3 or 5.
12. Seal each DNeasy 96 plate with a new AirPore Tape Sheet (provided). Centrifuge for 5 min at 6000 rpm.

13. Remove the tape. Carefully add 500 µl Buffer AW2 to each sample.

    Note: Ensure that ethanol has been added to Buffer AW2 prior to use.

    It is not necessary to increase the volume of Buffer AW2 if the volume of Proteinase K–Buffer ATL working solution was increased in steps 3 or 5.

14. Centrifuge for 15 min at 6000 rpm.

    Do not seal the plate with AirPore Tape.

    The heat generated during centrifugation ensures evaporation of residual ethanol in the sample (from Buffer AW2) that might otherwise inhibit downstream reactions.

15. Place each DNeasy 96 plate in the correct orientation on a new rack of Elution Microtubes RS (provided).

16. To elute the DNA, add 200 µl Buffer AE to each sample, and seal the DNeasy 96 plates with new AirPore Tape Sheets (provided). Incubate for 1 min at room temperature. Centrifuge for 2 min at 6000 rpm.

    To elute up to 75% of the DNA from each well of the DNeasy 96 plate, 200 µl Buffer AE is required.

    Elution with volumes less than 200 µl significantly increases the final DNA concentration of the eluate but may reduce overall DNA yield. For samples containing less than 1 µg DNA, elution in 50 µl Buffer AE is recommended.

17. **Recommended**: For maximum DNA yield, repeat step 16 with another 200 µl Buffer AE.

    A second elution with 200 µl Buffer AE will increase the total DNA yield by up to 25%. However due to the increased volume, the DNA concentration is reduced. If a higher DNA concentration is desired, the second elution step can be performed using the 200 µl eluate from the first elution. This will increase the yield by up to 15%.

    Use new caps (provided) to seal the Elution Microtubes RS for storage.

Protocol: Pretreatment for Paraffin-Embedded Tissue

This protocol is designed for purification of total DNA from fixed, paraffin-embedded tissues using the DNeasy Blood & Tissue Kit. The protocol describes the preliminary removal of paraffin by extraction with xylene.

Important points before starting

- The length of DNA purified from fixed tissues is usually <650 bp, depending on the type and age of the sample and the quality of the fixative used.
- Use of fixatives, such as alcohol and formalin, is recommended. Fixatives that cause cross-linking, such as osmic acid, are not recommended as it can be difficult to obtain amplifiable DNA from tissue fixed with these agents.
- Lysis time will vary from sample to sample depending on the type of tissue processed.
- Yields will depend both on the size and the age of the sample processed. Reduced yields compared with fresh or frozen tissues are to be expected. Therefore, eluting purified DNA in 50–100 µl Buffer AE is recommended.
- This pretreatment protocol has not been thoroughly tested and optimized for high-throughput DNA purification using the DNeasy 96 Blood & Tissue Kit. As a general guideline, we recommend decreasing the amount of starting material when using this protocol with the DNeasy 96 Blood & Tissue Kit.

Things to do before starting

- Preheat a heating block, incubator or water bath to 37°C for use in step 9.

Procedure

1. Place a small section (not more than 25 mg) of paraffin-embedded tissue in a 2 ml microcentrifuge tube (not provided).
2. Add 1200 µl xylene. Vortex vigorously.
3. Centrifuge in a microcentrifuge at full speed for 5 min at room temperature (15–25°C).
4. Remove supernatant by pipetting. Do not remove any of the pellet.
5. Add 1200 µl ethanol (96–100%) to the pellet to remove residual xylene, and mix gently by vortexing.
6. Centrifuge in a microcentrifuge at full speed for 5 min at room temperature.
7. Carefully remove the ethanol by pipetting. Do not remove any of the pellet.
8. Repeat steps 5–7 once.
9. Incubate the open microcentrifuge tube at 37° C for 10–15 min until the ethanol has evaporated.
10. Resuspend the tissue pellet in 180 µl Buffer ATL, and continue with step 2 of the protocol “Purification of Total DNA from Animal Tissues (Spin-Column Protocol)”, page 30.

Protocol: Pretreatment for Formalin-Fixed Tissue

This protocol is designed for purification of total DNA from fixed, paraffin-embedded tissues. The protocol describes the preliminary washing with PBS to remove the fixative.

Important points before starting

- The length of DNA purified from fixed tissues is usually <650 bp, depending on the type and age of the sample and the quality of the fixative used.
- Use of fixatives, such as alcohol and formalin, is recommended. Fixatives that cause cross-linking, such as osmic acid, are not recommended as it can be difficult to obtain amplifiable DNA from tissue fixed with these agents.
- Lysis time will vary from sample to sample depending on the type of tissue processed.
- Yields will depend both on the size and the age of the sample processed. Reduced yields compared with fresh or frozen tissues are to be expected. Therefore, eluting purified DNA in a total volume of 50–100 µl Buffer AE is recommended.
- This pretreatment protocol has not been thoroughly tested and optimized for high-throughput DNA purification using the DNeasy 96 Blood & Tissue Kit. As a general guideline, we recommend decreasing the amount of starting material when using this protocol with the DNeasy 96 Blood & Tissue Kit.

Procedure

1. Wash the sample (not more than 25 mg) twice in PBS to remove the fixative.
2. Discard the PBS and continue with step 1 of the protocol "Purification of Total DNA from Animal Tissues (Spin-Column Protocol)", page 30.

Protocol: Pretreatment for Gram-Negative Bacteria

This protocol is designed for purification of total DNA from Gram-negative bacteria, such as *E. coli*. The protocol describes the preliminary harvesting of bacteria before DNA purification.

Important points before starting

- See “Quantification of starting material”, page 17, for details of how to collect and store samples, and how to determine the number of cells in a bacterial culture.
- This pretreatment protocol has not been thoroughly tested and optimized for high-throughput DNA purification using the DNeasy 96 Blood & Tissue Kit. As a general guideline, we recommend decreasing the amount of starting material when using this protocol with the DNeasy 96 Blood & Tissue Kit.

Procedure

- Harvest cells (maximum 2 x 10^9 cells) in a microcentrifuge tube by centrifuging for 10 min at 5000 x *g* (7500 rpm). Discard supernatant.
- Resuspend pellet in 180 µl Buffer ATL.
- Continue with step 2 of the protocol “Purification of Total DNA from Animal Tissues (Spin-Column Protocol)”, page 30.

Protocol: Pretreatment for Gram-Positive Bacteria

This protocol is designed for purification of total DNA from Gram-positive bacteria, such as *Corynebacterium* spp. and *B. subtilis*. The protocol describes the preliminary harvesting of bacteria and incubation with lysozyme to lyse their cell walls before DNA purification.

Important points before starting

- See "Quantification of starting material", page 17, for details of how to collect and store samples, and how to determine the number of cells in a bacterial culture.
- Ensure that ethanol has not been added to Buffer AL (see "Buffer AL", page 19).
  Buffer AL can be purchased separately (see ordering information starting on page 59).
- This pretreatment protocol has not been thoroughly tested and optimized for high-throughput DNA purification using the DNeasy 96 Blood & Tissue Kit. As a general guideline, we recommend decreasing the amount of starting material when using this protocol with the DNeasy 96 Blood & Tissue Kit.

Things to do before starting

- Prepare enzymatic lysis buffer as described in "Equipment and Reagents to Be Supplied by User", page 13.
- Preheat a heating block or water bath to 37°C for use in step 3.

Procedure

1. Harvest cells (maximum 2 x 10^9 cells) in a microcentrifuge tube by centrifuging for 10 min at 5000 x *g* (7500 rpm). Discard supernatant.
2. Resuspend bacterial pellet in 180 µl enzymatic lysis buffer.
3. Incubate for at least 30 min at 37°C.

   After incubation, heat the heating block or water bath to 56°C if it is to be used for the incubation in step 5.

4. Add 25 µl Proteinase K and 200 µl Buffer AL (without ethanol). Mix by vortexing.

   Note: Do not add Proteinase K directly to Buffer AL. Ensure that ethanol has not been added to Buffer AL (see "Buffer AL", page 19). Buffer AL can be purchased separately (see ordering information starting on page 59).

5. Incubate at 56°C for 30 min.

   Optional: If required, incubate at 95°C for 15 min to inactivate pathogens. Note that incubation at 95°C can lead to some DNA degradation.

6. Add 200 µl ethanol (96–100%) to the sample, and mix thoroughly by vortexing.

   It is important that the sample and the ethanol are mixed thoroughly to yield a homogeneous solution.

   A white precipitate may form on addition of ethanol. It is essential to apply all of the precipitate to the DNeasy Mini spin column. This precipitate does not interfere with the DNeasy procedure.

7. Continue with step 4 of the protocol "Purification of Total DNA from Animal Tissues Spin-Column Protocol)", page 30.

Troubleshooting Guide

This troubleshooting guide may be helpful in solving any problems that may arise. For more information, see also the Frequently Asked Questions page at our Technical Support Center: **www.qiagen.com/FAQ/FAQList.aspx**. The scientists in QIAGEN Technical Services are always happy to answer any questions you may have about either the information and protocols in this handbook or sample and assay technologies (for contact information, see back cover or visit **www.qiagen.com**).

		Comments and suggestions
**Low yield**		
a)	Storage of starting material	DNA yield is dependent on the type, size, age and storage of starting material. Lower yields will be obtained from material that has been inappropriately stored (see "Sample collection and storage", page 15).
b)	Too much starting material	In future preparations, reduce the amount of starting material used (see "Quantification of starting material", page 17).
c)	Insufficient mixing of sample with Buffer AL and ethanol before binding	**DNeasy spin-column protocols**: In future preparations, mix sample first with Buffer AL and then with ethanol by pulse vortexing for15 s each time before applying the sample to the DNeasy Mini spin column.<br>**DNeasy 96 protocols**: In future preparations, ensure that samples are mixed by vigorous shaking, as described in the protocols, before applying the sample to the DNeasy 96 plate.
d)	DNA inefficiently eluted	Increase elution volume to 200 µl and perform another elution step. See also "Elution of pure nucleic acids", page 23. Check that ethanol was added before applying the sample to the DNeasy Mini spin column. Check that any precipitate in Buffer ATL and/or Buffer AL was dissolved before use.
e)	Buffer AW1 or Buffer AW2 prepared incorrectly	Make sure that ethanol has been added to Buffer AW1 and Buffer AW2 before use (see "Things to do before starting", pages 26, 30, 34, and 39).
f)	Water used instead of Buffer AE for elution	The low pH of deionized water from some water purifiers may reduce DNA yield. When eluting with water, ensure that the pH of the water is at least 7.0.

		Comments and suggestions
g)	Animal tissue: Insufficient lysis	In future preparations, reduce the amount of starting material used (see "Quantification of starting material", page 17).<br>Cut tissue into smaller pieces to facilitate lysis. After lysis, vortex sample vigorously; this will not damage or reduce the size of the DNA.<br>If a substantial gelatinous pellet remains after incubation and vortexing, extend incubation time at 56°C for Proteinase K digest and/or increase amount of Proteinase K to 40 µl. (For DNeasy 96 protocols, always check that the sample is completely lysed before addition of Buffer AL and ethanol. If a gelatinous mass is still present after the overnight incubation, lysis needs to be extended.)<br>Ensure that the sample is fully submerged in the buffer containing Proteinase K. If necessary, double the amount of Buffer ATL and Proteinase K, and use a 2 ml microcentrifuge tube for lysis. Remember to adjust the amount of Buffer AL and ethanol proportionately in subsequent steps. (For example, a lysis step with 360 µl Buffer ATL plus 40 µl Proteinase K will require 400 µl Buffer AL plus 400 µl ethanol to bind DNA to the DNeasy membrane).<br>**DNeasy spin-column protocols**: Pipet the sample into the DNeasy Mini spin column in two sequential loading steps. Discard flow-through between these loading steps.<br>**DNeasy 96 protocols**: Transfer a maximum of 900 µl of each sample to the DNeasy 96 plate.
h)	Bacteria: Insufficient lysis	In future preparations, extend incubation with cell-wall–lysing enzyme and/or increase amount of lysing enzyme. Harvest bacteria during early log phase of growth (see "Sample collection and storage", page 15).
i)	**DNeasy spin-column protocols**: DNA not bound to DNeasy Mini spin column.	Check that ethanol was added before applying the sample to the DNeasy Mini spin column
j)	**DNeasy 96 protocols**: Inefficient DNA elution	Repeat elution with Buffer AE preheated to 70°C.<br>After addition of Buffer AE preheated to 70°C, the DNeasy 96 plate should be incubated at room temperature (15–25°C) for 1 min. To increase elution efficiency, extend the incubation to 5 min at 70°C.
k)	**DNeasy 96 protocols**: Unequal volumes of Buffer AE or water delivered by the multichannel pipette	Ensure that all tips are firmly fitted to the pipette. Check liquid levels in tips before dispensing.

		Comments and suggestions
**DNeasy Mini spin column or DNeasy 96 plate clogged**		
	Too much starting material and/or insufficient lysis	Increase *g*-force and/or duration of centrifugation step. In future preparations, reduce the amount of starting material used (see "Quantification of starting material", page 17). For rodent tails or bacteria, see also "Insufficient lysis" in the "Low yield" section above.
**Low concentration of DNA in the eluate**		
	Second elution step diluted the DNA	Use a new collection tube for the second eluate to prevent dilution of the first eluate. Reduce elution volume to 50–100 µl. See "Elution of pure nucleic acids", page 23.
**A_{260}/A_{280} ratio of purified DNA is low**		
a)	Water used instead of buffer to measure A_{260}/A_{280}	Use 10 mM Tris·Cl, pH 7.5 instead of water to dilute the sample before measuring purity. See "Appendix A: Determination of Yield, Purity and Length of DNA", page 56.
b)	Inefficient cell lysis	See "Low yield", above.
**A_{260}/A_{280} ratio of purified DNA is high**		
	High level of residual RNA	Perform the optional RNase treatment in the protocol.
**DNA does not perform well in downstream applications**		
a)	Salt carryover	Ensure that Buffer AW2 has been used at room temperature. Ensure that Buffer AW1 and Buffer AW2 were added in the correct order.
b)	Ethanol carryover	**DNeasy spin-column protocols**: Ensure that, when washing with Buffer AW2, the column is centrifuged for 3 min at 20,000 x *g* (14,000 rpm) to dry the DNeasy membrane. Following the centrifugation step, remove the DNeasy Mini spin column carefully so that the column does not come into contact with the flow-through. If ethanol is visible in the DNeasy Mini spin column (as either drops or a film), discard the flow-through, keep the collection tube, and centrifuge for a further 1 min at 20,000 x *g*.<br><br>**DNeasy 96 protocols**: Incubate the DNeasy 96 plate, uncovered, in an oven or incubator for 10 min at 80°C after the second wash to remove all traces of BufferAW2.
c)	Too much DNA used	For PCR applications, a single-copy gene can typically be detected after 35 PCR cycles with 100 ng template DNA.

	Comments and suggestions
**DNA sheared**	
a) Sample repeatedly frozen and thawed	Avoid repeated freezing and thawing of. starting material.
b) Sample too old	Old samples often yield only degraded DNA
**White precipitate in Buffer ATL or Buffer AL**	
White precipitate may form at low temperature after prolonged storage	Any precipitate formed when Buffer ATL or Buffer AL are added must be dissolved by incubating the buffer at 56°C until it disappears.
**Discolored membrane after wash with Buffer AW2 or colored eluate**	
a) Rodent tails: Hair not removed from rodent tails during preparation	**DNeasy spin-column protocols**: In future preparations, centrifuge lysate for 5 min at 20,000 x *g* after digestion with Proteinase K. Transfer supernatant into a new tube before proceeding with step 3.<br>**DNeasy 96 protocols**: In future preparations, centrifuge the rack of collection microtubes containing the lysates for 5 min at 6000 rpm at step 5. Remove the caps. Carefully transfer the lysates, without disturbing the pelleted debris, to another rack of collection microtubes. Continue the protocol at step 6.
b) Animal blood: Contamination with hemoglobin	Reduce amount of blood used and/or double the amount of Proteinase K used per preparation. Try using buffy coat instead of whole blood.

Appendix A: Determination of Yield, Purity and Length of DNA

Determination of yield and purity

DNA yield is determined by measuring the concentration of DNA in the eluate by its absorbance at 260 nm. Absorbance readings at 260 nm should fall between 0.1 and 1.0 to be accurate. Sample dilution should be adjusted accordingly. Measure the absorbance at 260 nm or scan absorbance from 220–330 nm (a scan will show if there are other factors affecting absorbance at 260 nm; for instance, absorbance at 325 nm would indicate contamination by particulate matter or a dirty cuvette). An A_{260} value of 1 (with a 1 cm detection path) corresponds to 50 µg DNA per milliliter water. Water should be used as diluent when measuring DNA concentration since the relationship between absorbance and concentration is based on extinction coefficients calculated for nucleic acids in water.* Both DNA and RNA are measured with a spectrophotometer at 260 nm; to measure only DNA in a mixture of DNA and RNA, a fluorimeter must be used.

An example of the calculations involved in DNA quantification is shown below.

Volume of DNA sample	= 100 µl
Dilution	= 20 µl of DNA sample + 180 µl distilled water (1/10 dilution)

Measure absorbance of diluted sample in a 0.2 ml cuvette

A_{260}	= 0.2
Concentration of DNA sample	= 50 µg/ml x A_{260} x dilution factor
	= 50 µg/ml x 0.2 x 10
	= 100 µg/ml
Total amount	= concentration x volume of sample in milliliters
	= 100 µg/ml x 0.1 ml
	= 10 µg DNA

* Wilfinger, W.W., Mackey, M., and Chomcynski, P. (1997) Effect of pH and ionic strength on the spectrophotometric assessment of nucleic acid purity. BioTechniques **22**, 474.

The ratio of the readings at 260 nm and 280 nm (A_{260}/A_{280}) provides an estimate of the purity of DNA with respect to contaminants that absorb UV, such as protein. However, the A_{260}/A_{280} ratio is influenced considerably by pH. Since water is not buffered, the pH and the resulting A_{260}/A_{280} ratio can vary greatly. Lower pH results in a lower A_{260}/A_{280} ratio and reduced sensitivity to protein contamination. For accurate values, we recommend measuring absorbance in 10 mM Tris·Cl, pH 7.5, in which pure DNA has an A_{260}/A_{280} ratio of 1.8–2.0. Always be sure to calibrate the spectrophotometer with the same solution.

Determination of length

The precise length of genomic DNA should be determined by pulse-field gel electrophoresis (PFGE) through an agarose gel. To prepare the sample for PFGE, the DNA should be concentrated by alcohol precipitation and the DNA pellet dried briefly at room temperature (15–25°C) for 5–10 minutes. Avoid drying the DNA pellet for more than 10 minutes since overdried genomic DNA is very difficult to redissolve. Redissolve in approximately 30 µl TE buffer, pH 8.0,* for at least 30 minutes at 60°C. Load 3–5 µg of DNA per well. Standard PFGE conditions are as follows:

- 1% agarose gel in 0.5x TBE electrophoresis buffer*
- Switch intervals = 5–40 seconds
- Run time = 17 hours
- Voltage = 170 V

*When working with chemicals, always wear a suitable lab coat, disposable gloves, and protective goggles. For more information, consult the appropriate safety data sheets (SDSs), available from the product supplier.

Appendix B: Cleaning S-Blocks

Cleaning S-Blocks

Cleaning S-Blocks To avoid cross-contamination, after each use rinse the S-Blocks thoroughly in tap water, incubate for 1 min at room temperature (15–25°C) in 0.4 M HCl,* empty, and wash thoroughly with distilled water. Used S-Blocks can also be autoclaved after washing. Additional S-Blocks can be ordered separately (see ordering information starting on page 59).

* When working with chemicals, always wear a suitable lab coat, disposable gloves, and protective goggles. For more information, consult the appropriate safety data sheets (SDSs), available from the product supplier.

Ordering Information

Product	Contents	Cat. no.
DNeasy Blood & Tissue Kit (50)	50 DNeasy Mini Spin Columns, Proteinase K, Buffers, Collection Tubes (2 ml)	69504
DNeasy Blood & Tissue Kit (250)	250 DNeasy Mini Spin Columns Proteinase K, Buffers, Collection Tubes (2 ml)	69506
DNeasy 96 Blood & Tissue Kit (4)*	For 4 x 96 DNA minipreps: 4 DNeasy 96 Plates, Proteinase K, Buffers, S-Blocks, AirPore Tape Sheets, Collection Microtubes (1.2 ml), Elution Microtubes RS, Caps, 96-Well Plate Registers	69581
DNeasy 96 Tissue Kit (12)	For 12 x 96 DNA minipreps: 12 DNeasy 96 Plates, Proteinase K, Buffers, S-Blocks, AirPore Tape Sheets, Collection Microtubes (1.2 ml), Elution Microtubes RS, Caps, 96-Well Plate Registers	69582
**QIAcube Connect — for fully automated nucleic acid extraction with QIAGEN spin-column kits**		
QIAcube Connect†	Instrument, connectivity package, 1 year warranty on parts and labor	Inquire
Starter Pack, QIAcube	Reagent bottle racks (3); 200 µl filter-tips (1024); 1000 µl filter-tips (1024); 30 ml reagent bottles (12); rotor adapters (240); rotor adapter holder	990395

* Larger kit sizes and/or formats available; see **www.qiagen.com**.

† All QIAcube Connect instruments are provided with a region-specific connectivity package, including tablet and equipment necessary to connect to the local network. Further, QIAGEN offers comprehensive instrument service products, including service agreements, installation, introductory training and preventive subscription. Contact your local sales representative to learn about your options.

Product	Contents	Cat. no.
**QIAGEN 96-Well Plate Centrifugation System**		
Centrifuge 4-16S	Universal laboratory centrifuge with brushless motor	Inquire
Centrifuge 4-16KS	Universal refrigerated laboratory centrifuge with brushless motor	Inquire
Plate Rotor 2 x 96	Rotor for 2 QIAGEN 96-well plates for use with QIAGEN Centrifuges	81031
**Accessories**		
Collection Tubes (2 ml)	1000 Collection Tubes (2 ml)	19201
Collection Microtubes (racked, 10 x 96)	Nonsterile polypropylene tubes (1.2 ml), 960 in racks of 96	19560
Collection Microtube Caps (120 x 8)	Nonsterile polypropylene caps for collection microtubes (1.2 ml) and round-well blocks, 960 in strips of 8	19566
S-Blocks (24)	96-well blocks with 2.2 ml wells, 24 per case	19585
AirPore Tape Sheets (50)	Microporous tape sheets for covering 96-well blocks: 50 sheets per pack	19571
TissueRuptor II	Handheld rotor–stator homogenizer	Inquire
TissueRuptor II Disposable Probes (25)	25 nonsterile plastic disposable probes for use with the TissueRuptor II	990890
TissueLyser II	Universal laboratory mixer-mill disruptor	Inquire
TissueLyser Adapter Set 2 x 24	2 sets of Adapter Plates and 2 racks or use with 2.0 ml microcentrifuge tubes on the TissueLyser II	69982

Product	Contents	Cat. no.
TissueLyser Adapter Set 2 x 96	2 sets of Adapter Plates for use with Collection Microtubes (racked) on the TissueLyser II	69984
Stainless Steel Beads, 5 mm (200)	Stainless Steel Beads, suitable for use with the TissueLyser II system	69989
QIAGEN Proteinase K (2 ml)	2 ml (>600 mAU/ml, solution)	19131
QIAGEN Proteinase K (10 ml)	10 ml (>600 mAU/ml, solution)	19133
RNase A (17,500 U)	2.5 ml (100 mg/ml; 7000 units/ml, solution)	19101
Buffer AL (216 ml)	216 ml Lysis Buffer	19075
Buffer ATL (200 ml)	200 ml Tissue Lysis Buffer for 1000 preps	19076
Buffer AW1 (Concentrate, 242 ml)	242 ml Wash Buffer (1) Concentrate	19081
Buffer AW2 (Concentrate, 324 ml)	324 ml Wash Buffer (2) Concentrate	19072
Buffer AE (240 ml)	240 ml Elution Buffer	19077
**Related products**		
QIAGEN Genomic-tip 20/G	25 columns	10223
QIAGEN Genomic-tip 100/G	25 columns	10243
QIAGEN Genomic-tip 500/G	10 columns	10262
Blood & Cell Culture DNA Mini Kit (25)	25 QIAGEN Genomic-tip 20/G, QIAGEN Protease, Buffers	13323

Product	Contents	Cat. no.
Blood & Cell Culture DNA Midi Kit (25)	25 QIAGEN Genomic-tip 100/G, QIAGEN Protease, Buffers	13343
Blood & Cell Culture DNA Maxi Kit (10)	10 QIAGEN Genomic-tip 500/G, QIAGEN Protease, Buffers	13362
BioSprint 15 DNA Blood Kit (45)*	For 45 preps on the BioSprint 15 workstation: 5-Rod Covers	940014
BioSprint® 96 DNA Blood Kit (48)*	For 48 preps on the BioSprint 96 workstation: Large 96-Rod Covers, 96-Well Microplates MP, S-Blocks, MagAttract Suspension G, Buffers and Reagents	940054
RNeasy® Mini Kit (50)*	For 50 RNA minipreps: 50 RNeasy Mini Spin Columns, Collection Tubes (1.5 ml and 2 ml), RNase-free Reagents and Buffers	74104
RNeasy Maxi Kit (12)	For 12 RNA maxipreps: 12 RNeasy Maxi Spin Columns, Collection Tubes (50 ml), RNase-free Reagents and Buffers	75162
RNeasy Protect Mini Kit (50)*	For RNA stabilization and 50 RNA minipreps: RNAprotect® RNA Stabilization Reagent (50 ml), 50 RNeasy Mini Spin Columns, Collection Tubes (1.5 ml and 2 ml), RNase-free Reagents and Buffers	74124

* Larger kit sizes and/or formats available; see **www.qiagen.com**.

† All QIAcube Connect instruments are provided with a region-specific connectivity package, including tablet and equipment necessary to connect to the local network. Further, QIAGEN offers comprehensive instrument service products, including service agreements, installation, introductory training and preventive subscription. Contact your local sales representative to learn about your options.

For up-to-date licensing information and product-specific disclaimers, see the respective QIAGEN kit handbook or user manual. QIAGEN kit handbooks and user manuals are available at **www.qiagen.com** or can be requested from QIAGEN Technical Services or your local distributor.

Document Revision History

Date	Changes
01/2020	Updated text, ordering information and intended use for QIAcube Connect.
07/2020	Updated quantities in "Kit Contents". Added warning for Buffers AL and AW1 in "Safety Information". Corrected step-numbering error in "Protocol: Purification of Total DNA from Animal Blood or Cells (DNeasy 96 Protocol)" and restored missing step 2 for Buffer AL addition. Updated recommended centrifuge models. Added missing word in Appendix A. Replaced reference to RNA*later* with RNAprotect. Deleted PCR and QIAzol disclaimers.

Limited License Agreement for the DNeasy Blood & Tissue Kit and DNeasy 96 Blood & Tissue Kit

Use of this product signifies the agreement of any purchaser or user of the product to the following terms:

1. The product may be used solely in accordance with the protocols provided with the product and this handbook and for use with components contained in the kit only. QIAGEN grants no license under any of its intellectual property to use or incorporate the enclosed components of this kit with any components not included within this kit except as described in the protocols provided with the product, this handbook, and additional protocols available at www.qiagen.com. Some of these additional protocols have been provided by QIAGEN users for QIAGEN users. These protocols have not been thoroughly tested or optimized by QIAGEN. QIAGEN neither guarantees them nor warrants that they do not infringe the rights of third-parties.
2. Other than expressly stated licenses, QIAGEN makes no warranty that this kit and/or its use(s) do not infringe the rights of third-parties.
3. This kit and its components are licensed for one-time use and may not be reused, refurbished, or resold.
4. QIAGEN specifically disclaims any other licenses, expressed or implied other than those expressly stated.
5. The purchaser and user of the kit agree not to take or permit anyone else to take any steps that could lead to or facilitate any acts prohibited above. QIAGEN may enforce the prohibitions of this Limited License Agreement in any Court, and shall recover all its investigative and Court costs, including attorney fees, in any action to enforce this Limited License Agreement or any of its intellectual property rights relating to the kit and/or its components.

For updated license terms, see **www.qiagen.com**.

Trademarks: QIAGEN®, Sample to Insight®, QIAcube®, BioSprint®, DNeasy®, MagAttract®, RNeasy®, TissueRuptor® (QIAGEN Group); DU® (Beckman Instruments, Inc.); Impact® (Matrix Technologies Corp.); Triton® (Union Carbide Corporation). Registered names, trademarks, etc. used in this document, even when not specifically marked as such, are not to be considered unprotected by law.

07/2020 HB-2061-003 © 2020 QIAGEN, all rights reserved.

HB-2061-003 07/2020
