# Supplementary material for: Genomic DNA extraction optimization and validation for genome sequencing using the marine gastropod Kellet’s whelk
Source: PeerJ. 2023 Dec 6;11:e16510. doi: 10.7717/peerj.16510 (PMC10710129; doi:10.7717/peerj.16510)
Supplement: Supplemental Information 7 [file peerj-11-16510-s007.zip › Zymo Clean and Concentrator.pdf]

ZYMO RESEARCH

DNA Purification Made Simple

DNA Clean & Concentrator®-5

For recovery of ultra-pure DNA from PCR, enzymatic reactions, and other sources.

Highlights

- Quick, 2 minute recovery of ultra-pure DNA from PCR, enzymatic reactions, and other sources.
- DNA can be eluted in as little as 6 µl and is ideal for DNA ligation, sequencing, labeling, PCR, microarray, transfection, transformation, restriction digestion, *etc*.

Catalog Numbers:
D4003T, D4003, D4004, D4013, D4014

Scan with your smart-phone camera to view the online protocol/video.

tech@zymoresearch.com www.zymoresearch.com Toll Free: (888) 882-9682

Table of Contents

Product Contents 01
Specifications 02
Product Description 03
Formats 04
Applications 04
Protocol 05
Buffer Preparation 05
Sample Processing 05
Appendix 06
cDNA Clean-Up 06
M13 phage ssDNA Purification 06
Isolated DNA stored in DNA/RNA Shield .. 07
RNase A Treatment 07
Troubleshooting 08
Ordering Information 09
Complete Your Cloning Workflow 10
Notes 11
Guarantee 13

Product Contents

DNA Clean & Concentrator®-5	D4003T (10 Preps.)	D4003, D4013 (50 Preps.)	D4004, D4014 (200 Preps.)	Storage Temperature
DNA Binding Buffer	10 ml	50 ml	2 x 100 ml	Room Temp.
DNA Wash Buffer[1]	6 ml	6 ml	24 ml	Room Temp.
DNA Elution Buffer	1 ml	1 ml	4 ml	Room Temp.
Zymo-Spin™ Columns	10<br>uncapped	50<br>D4003 – uncapped<br>D4013 – capped	200<br>D4004 – uncapped<br>D4014 – capped	Room Temp.
Collection Tubes	10	50	200	Room Temp.
Instruction Manual	1	1	1	-

[1] Ethanol must be added prior to use as indicated on the **DNA Wash Buffer** label. DNA Wash Buffer included with D4003S and D4003T is supplied ready-to-use and does not require the addition of ethanol prior to use.

Specifications

- **DNA Purity –** High-quality DNA ($A_{(260/280)}$ ≥ 1.8) ideal for ligation, sequencing, labeling, PCR, microarray, transfection, transformation, and restriction digestion procedures.

- **DNA Size Limits –** From ~50 bp to 23 kb.

- **DNA Recovery –** Typically, up to 5 µg total DNA per column can be eluted into as little as 6 µl of low salt **DNA Elution Buffer** or water. For DNA 50 bp to 10 kb, the recovery is 70-90%. For DNA 11 kb to 23 kb, the recovery is 50-70%.

- **Sample Sources –** DNA from enzymatic reactions (*e.g.*, PCR, restriction endonuclease digestions), plasmid preparations, and impure preparations. Suitable for isolated DNA stored in DNA/RNA Shield (page 7).

- **Product Detergent Tolerance –** ≤5% Triton X-100, ≤5% Tween-20, ≤5% Sarkosyl, ≤ 0.1% SDS.

Product Description

The **DNA Clean & Concentrator®-5** (**DCC®-5**) provides a hassle-free method for the rapid purification and concentration of high-quality DNA from PCR, endonuclease digestions, cell lysates, and other impure DNA preparations. It can also be used for post-RT cDNA clean-up and purification of sequencing-ready DNA from M13 phage. Simply add the specially formulated **DNA Binding Buffer** to your sample and transfer the mixture to the supplied **Zymo-Spin™ Column**. There is no need for organic denaturants or chloroform. Instead, the product features *Fast-Spin* column technology to yield DNA that is free of salts and contaminants in just 2 minutes. The purified DNA is ideal for DNA ligation, sequencing, labeling, PCR, microarray, transfection, transformation, and restriction digestion procedures.

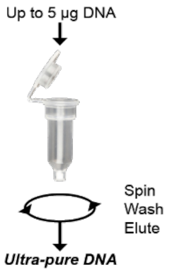

Two minute **DCC®-5** procedure

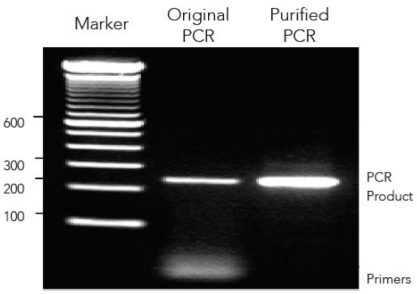

DNA samples, such as the PCR products shown here, can be efficiently purified and concentrated using the **DNA Clean & Concentrator®-5**.

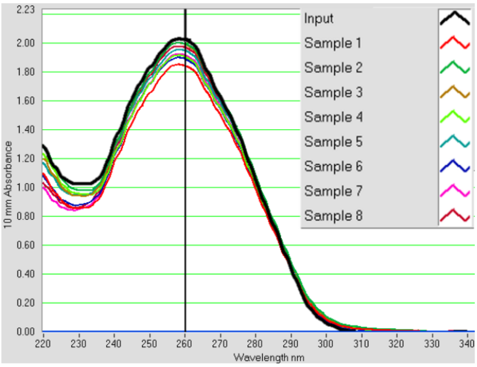

Pure and Reliable Recovery with the DCC®-5. Shown here is the recovery of 1 µg of 100 bp marker DNA eluted into 10 µl of water analyzed using a NanoDrop® spectrophotometer. The DNA Clean & Concentrator®-5 consistently recovers > 90 % of input DNA.

Formats

	DCC™-5	DCC™-25	DCC™-100	DCC™-500	Genomic DCC™	ZR-96 DCC™-5
	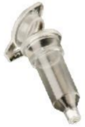	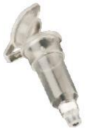	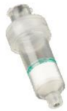	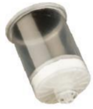	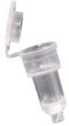	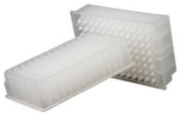
Name	Zymo-Spin™ I & IC	Zymo-Spin™ II & IIC	Zymo-Spin™ V	Zymo-Spin™ VI	Zymo-Spin ™ IC-XL	Zymo-Spin™ I-96
Capacity	5 µg/ prep.	25 µg/ prep.	100 µg/ prep.	500 µg/ prep.	10 µg/ prep.	5 µg/ prep.
Elution Vol.	≥ 6 µl	≥ 25 µl	≥ 150 µl	≥ 2 ml	≥ 10 µl	≥ 10 µl
Cat. Nos.	D4003, D4013	D4005, D4033	D4029, D4030	D4031, D4032	D4010, D4011	D4023, D4024

Applications

**Post-PCR DNA Clean-up**	Efficient desalting of DNA with the removal of DNA polymerases, primers, and free dNTPs.
**DNA Clean-up From Enzymatic Reactions**	Efficient desalting of DNA with the removal of modifying enzymes, RNA polymerases, ligases, kinases, nucleases, phosphatases, endonucleases, *etc.*
**Post-Reverse Transcription (RT) & cDNA Clean-up**	Efficiently purifies DNA following RT, either as a DNA/RNA complex or as single stranded cDNA following chemical hydrolysis of the RNA template.
**Plasmid DNA Clean-up**	Efficiently purifies plasmid DNA from "home-made" preparations of cell free lysates or from commercial kits. Plasmid DNA purified and concentrated using the **DCC®** has proven an excellent substrate for high quality DNA sequencing.
**Isotope and Dye Removal**	Efficiently removes unincorporated fluorescent (*i.e.*, AMCA, FITC, BIO, DIG, Cy3, Cy5, FAM, *etc.*) and radiolabeled dNTP derivatives from DNA following *in vitro* labeling reactions.
**Purification of M13 ssDNA**	The **DCC®** can be used for the rapid isolation of single stranded M13 phage DNA directly from phage-infected *E. coli* culture supernatant.

✓ For purification of short DNA or RNA oligonucleotides ≥ 16 nt, use the **Oligo Clean & Concentrator (D4060, D4061)**.

✓ For ChIP (Chromatin Immunoprecipitation) sample cleanup, use the **ChIP DNA Clean & Concentrator® (D5201, D5205)** for high quality DNA from any step in a standard ChIP protocol.

✓ For post-cycle sequencing samples, use the **ZR Sequencing DNA Clean-up Kit™ (D4050, D4051)** for dye blob elimination.

✓ For samples containing PCR inhibitors, use the ***OneStep*™ PCR Inhibitor Removal Kit (D6030, D6035)**.

Protocol

Buffer Preparation

✓ *Before starting*: Add 24 ml 100% ethanol (26 ml 95% ethanol) to the 6 ml **DNA Wash Buffer** concentrate. Add 96 ml 100% ethanol (104 ml 95% ethanol) to the 24 ml **DNA Wash Buffer** concentrate.

✓ **DNA Wash Buffer** included with D4001S and D4001T is supplied ready-to-use and does not require the addition of ethanol prior to use.

Sample Processing

All centrifugation steps should be performed between 10,000 - 16,000 x g.

1. In a 1.5 ml microcentrifuge tube, add 2-7 volumes of **DNA Binding Buffer** to each volume of DNA sample (see table below). Mix briefly by vortexing.

Application	DNA Binding Buffer : Sample	Example
Plasmid, genomic DNA (>2 kb)[1]	2 : 1	200 µl : 100 µl
PCR product, DNA fragment	5 : 1	500 µl : 100 µl
ssDNA[2] (*e.g.* cDNA, M13 phage)	7 : 1	700 µl : 100 µl

2. Transfer mixture to a provided **Zymo-Spin™ Column**[3] in a **Collection Tube.**

3. Centrifuge for 30 seconds. Discard the flow-through.

4. Add 200 µl **DNA Wash Buffer** to the column. Centrifuge for 30 seconds. Repeat this wash step.

5. Add ≥ 6 µl **DNA Elution Buffer**[4] or water[5] directly to the column matrix and incubate at room temperature for one minute. Transfer the column to a 1.5 ml microcentrifuge tube and centrifuge for 30 seconds to elute the DNA. Ultra-pure DNA is now ready for use.

---

[1] For efficient recovery of DNA > 20 kb, use the **Genomic DNA Clean & Concentrator (D4010, D4011)**.
[2] For ssDNA purification, see page 6 in the appendix.
[3] The sample capacity of the column is 800 µl. Therefore, it may be necessary to load and spin a column multiple times if a sample has a volume larger than 800 µl.
[4] The **DNA Elution Buffer** contains10 mM Tris-HCl, pH 8.5, 0.1 mM EDTA.
[5] Elution of DNA from the column is dependent on pH and temperature. If water is used, make sure the pH is >6.0. Waiting 1 minute prior to elution may improve the yield of large (> 6 kb) DNA. For even larger DNA (> 10 kb), the total yield may be improved by eluting the DNA with 60-70°C **DNA Elution Buffer**.

Appendix

cDNA clean-up

*The **DCC®** kit can be used to effectively clean and concentrate cDNA (> 500 nt) following reverse transcription (RT) in the presence/absence of fluorescent dyes. Unincorporated free nucleotides and fluorescent derivatives are efficiently removed using the **DCC®**, and the recovered cDNA may be used directly for microarray analysis, second-strand cDNA synthesis, or indirect labeling with a fluorescent dye such as NHS ester Cy3 or Cy5.*

*For clean-up of short cDNAs or ESTs (≥ 16 nt), we recommend the **Oligo Clean & Concentrator (Cat. Nos. D4060, D4061)**.*

Hydrolysis

1. Add 10 µl 0.5 M EDTA and 10 µl 1 N NaOH to 50 µl of RT reaction.

   The volumes of EDTA and NaOH should be scaled proportionally depending on the starting volume of the RT reaction.

2. Incubate at 65°C for 15 minutes.

Clean-up

1. Add 490 µl (7 volumes) of **DNA Binding Buffer** to the hydrolysis reaction above. Mix well.

   Neutralization (pH) following RNA hydrolysis is not necessary as the **DNA Binding Buffer** will effectively neutralize the NaOH added to the reaction.

2. Continue with Step 2 of the Sample Processing Protocol on page 5.

M13 phage ssDNA purification

1. Centrifuge phage-infected bacterial culture at 8,000 x *g* for 1 minute.

2. Transfer 100 µl of phage-containing supernatant to a 1.5 ml microcentrifuge tube and add 700 µl (7 volumes) of **DNA Binding Buffer**. Mix briefly by vortexing.

   Increased supernatant volumes may be processed by proportionally increasing the amount of **DNA Binding Buffer** added to the sample.

3. Continue with Step 2 of the Sample Processing Protocol on page 5.

Isolated DNA stored in DNA/RNA Shield

*For previously isolated/purified DNA stored in **DNA/RNA Shield**, use the following protocol to recover ultra-pure DNA, ready for downstream applications.*

1. If frozen, thaw samples[1] at room temperature (20-30°C).

2. Add an equal volume of ethanol (95-100%) to the sample and mix well.

3. Continue with Step 2 of the Sample Processing Protocol on page 5.

RNase A Treatment

Dissolve RNase A (E1008-30), sold separately, in DNase/RNase-free water or TE to a stock concentration of 10 mg/ml.

1. Add enough 10 mg/ml RNase A to the sample for a final concentration of 10-100 µg/mL and mix well.

2. Incubate at room temperature for 15 minutes.

3. Continue with step 1 of the Sample Processing protocol on page 5.

[1] Adjust the sample volume to 50 µl (minimum) with **DNA/RNA Shield**.

Troubleshooting

Problem	Possible Causes and Suggested Solutions
**Low Recovery**	**Improperly Prepared/Stored DNA Wash Buffer.** Make sure ethanol has been added to the **DNA Wash Buffer** concentrate. Cap the bottle tightly to prevent evaporation over time.<br><br>**Addition of DNA Elution Buffer.** Add elution buffer directly to the column matrix, not to the walls of the column. Elution buffer requires contact with the matrix for at least 1 minute for large DNA ≥ 10 kb.<br><br>**Incomplete Elution.** DNA elution is dependent on pH, temperature, and time. For large genomic DNA (≥50 kb), apply heated elution buffer (60-70 °C) to the column and incubate for several minutes prior to elution. Sequential elutions may be performed for quantitatively higher recovery but lower final DNA concentration. This is recommended for DNA ≥ 10 kb.
**Low A_{260}/A_{230} ratio**	**Column tip contaminated.** When removing the column from the collection tube, be careful that the tip of the column does not come into contact with the flowthrough. Trace amounts of salt from the flowthrough can contaminate a sample resulting in a low A_{260}/A_{230} ratio. Ethanol contamination from the flowthrough can also interfere with DNA elution. Zymo-Spin™ columns are designed for complete elution with no buffer retention or carryover.
**Following Clean-up with DCC®, Multiple Bands Appear in an Agarose Gel**	**Acidification of DNA Loading Dye.** Most loading dyes do not contain EDTA and will acidify (pH ≤ 4) over time due to some microbial growth. This low pH is enough to cause DNA degradation. Therefore, if water is used to elute the DNA, 6X Loading Dye containing 1 mM EDTA is recommended.

Ordering Information

Product Description	Catalog No.	Size
**DNA Clean & Concentrator®-5**<br>(for purification of up to 5 µg DNA per prep.)	D4003T (uncapped)<br>D4003 (uncapped)<br>D4004 (uncapped)<br><br>D4013 (capped)<br>D4014 (capped)	10 Preps.<br>50 Preps.<br>200 Preps.<br><br>50 Preps.<br>200 Preps.
**ZR-96 DNA Clean & Concentrator®-5**<br>(for 96-well purification of up to 5 µg DNA per well)	D4023<br>D4024	2 x 96 Preps.<br>4 x 96 Preps.
**DNA Clean & Concentrator®-25**<br>(for purification of up to 25 µg DNA per prep.)	D4005 (uncapped)<br>D4006 (uncapped)<br><br>D4033 (capped)<br>D4034 (capped)	50 Preps.<br>200 Preps.<br><br>50 Preps.<br>200 Preps.
**DNA Clean & Concentrator®-100**<br>(for purification of up to 100 µg DNA per prep.)	D4029<br>D4030	25 Preps.<br>50 Preps.
**DNA Clean & Concentrator®-500**<br>(for purification of up to 500 µg DNA per prep.)	D4031<br>D4032	10 Preps.<br>20 Preps.
**Genomic DNA Clean & Concentrator®**<br>(for purification of up to 10 µg genomic DNA per prep.)	D4010<br>D4011	25 Preps.<br>100 Preps.

Individual Kit Components	Catalog No.	Amount
**DNA Binding Buffer**	D4003-1-L<br>D4004-1-L	50 ml<br>100 ml
**DNA Wash Buffer** (concentrate)	D4003-2-6<br>D4003-2-24	6 ml<br>24 ml
**DNA Elution Buffer**	D3004-4-1<br>D3004-4-4	1 ml<br>4 ml
**Zymo-Spin™ I Columns**	C1003-50 (uncapped)<br>C1003-250 (uncapped)<br><br>C1004-50 (capped)<br>C1004-250 (capped)	50 Pack<br>250 Pack<br><br>50 Pack<br>250 Pack
**Collection Tubes**	C1001-50<br>C1001-500<br>C1001-1000	50 Pack<br>500 Pack<br>1000 Pack

Complete Your Cloning Workflow

✓ **Transfection-grade plasmid DNA from a miniprep**

ZymoPURE™ Plasmid Miniprep	Size	Catalog No.
**ZymoPURE™ Plasmid Miniprep Kit**	10 Preps.<br>50 Preps.<br>100 Preps.<br>400 Preps.<br>800 Preps.	D4208T<br>D4209<br>D4210<br>D4211<br>D4212

✓ **20 Minute Endotoxin-Free Midi & Maxipreps**

ZymoPURE™ II Plasmid Prep Kits	Size	Catalog No.
**ZymoPURE™ II Plasmid Midiprep Kit**	25 Preps.<br>50 Preps.	D4200<br>D4201
**ZymoPURE™ II Plasmid Maxiprep Kit**	10 Preps.<br>20 Preps.	D4202<br>D4203
**ZymoPURE™ II Plasmid Gigaprep Kit**	5 Preps.	D4204

✓ **Simple 20 second High Efficiency Transformations**

Mix & Go! Competent Cells	Size	Catalog No.
**DH5α**	10 x 100 µl aliquots<br>96 x 50 µl aliquots<br>96 x 50 µl aliquots PCR Plate	T3007<br>T3009<br>T3010
**Zymo10B**	10 x 100 µl aliquots<br>96 x 50 µl aliquots	T3019<br>T3020
**JM109**	10 x 100 µl aliquots<br>96 x 50 µl aliquots	T3003<br>T3005
**HB101**	10 x 100 µl aliquots<br>96 x 50 µl aliquots	T3011<br>T3013
**TG1**	10 x 100 µl aliquots	T3017

✓ **Recover ultra-pure highly concentrated DNA from PCR & other sources**

DNA Clean & Concentrator™	Size	Catalog No.
**DNA Clean & Concentrator™-5**	50 Preps.<br>200 Preps.	D4003<br>D4004
**ZR-96 DNA Clean-Up Kit™**	2 x 96 Preps.<br>4 x 96 Preps.	D4017<br>D4018

✓ **Rapid extraction of ultra-pure DNA from agarose gels**

Zymoclean Gel DNA Recovery™	Size	Catalog No.
**Zymoclean™ Gel DNA Recovery Kit**	50 Preps.<br>200 Preps.	D4001<br>D4002

Notes

Notes

100% satisfaction guarantee on all Zymo Research products, or your money back.

Zymo Research is committed to simplifying your research with quality products and services. If you are dissatisfied with this product for any reason, please call 1(888) 882-9682.

Integrity of kit components is guaranteed for up to one year from date of purchase.
Reagents are routinely tested on a lot-to-lot basis to ensure they provide the highest performance and reliability.

This product is for research use only and should only be used by trained professionals. It is not for use in diagnostic procedures. Some reagents included with this kit are irritants. Wear protective gloves and eye protection. Follow the safety guidelines and rules enacted by your research institution or facility.

™ Trademarks of Zymo Research Corporation
NanoDrop® is a registered trademark of NanoDrop Technologies, Inc.

ZYMO RESEARCH

The **BEAUTY** *of* **SCIENCE** *is to Make Things* **SIMPLE**®

tech@zymoresearch.com www.zymoresearch.com Toll Free: (888) 882-9682
