# Supplementary material for: Genomic DNA extraction optimization and validation for genome sequencing using the marine gastropod Kellet’s whelk
Source: PeerJ. 2023 Dec 6;11:e16510. doi: 10.7717/peerj.16510 (PMC10710129; doi:10.7717/peerj.16510)
Supplement: Supplemental Information 7 [file peerj-11-16510-s007.zip › Guide-overview-Short-Read-Eliminator-SRE-XS-and-XL-kits.pdf]

Short Read Eliminator (SRE) XS and XL kits

Guide & overview

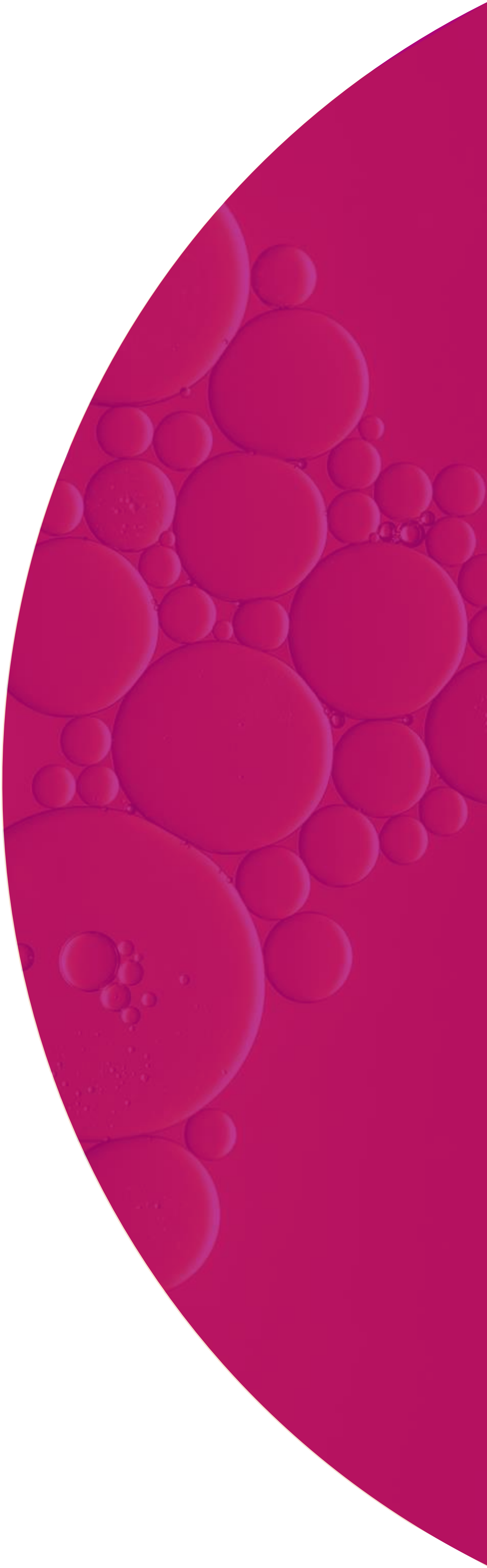

Table of contents

Prior to starting ... 3

Storage ... 3

Product use ... 3

User supplied equipment and reagent list ... 3

Introduction ... 4

Sample information and expected performance ... 5

HMW DNA size selection ... 6

Sheared/fragmented DNA size selection (SRE XS kit only) ... 7

Workflow ... 8

Processing tips ... 9

Pipetting ... 9

Heterogeneity and viscosity ... 9

Size selection protocol for SRE XL ... 10

HMW gDNA ... 10

Size selection protocol for SRE XS ... 11

HMW gDNA or sheared/fragmented DNA ... 11

Troubleshooting guide ... 12

© 2023 PacBio. All rights reserved. Research use only. Not for use in diagnostic procedures.

Prior to starting

Create 70% EtOH wash buffer by diluting ethanol (96–100%) with DI water.

Storage

All buffers should be stored at room temperature (15–30°C).

Product use

SRE XS and SRE XL kits are intended for research use only.

User supplied equipment and reagent list

Equipment	Model
SRE XS kit	PacBio® (102-208-200)
SRE kit*	PacBio (102-208-300)
SRE XL kit	PacBio (102-208-400)
1.5 mL DNA LoBind microcentrifuge tubes	Eppendorf (022431021)
200 µL wide bore pipette tips	USA Scientific (1011-8410)
Ethanol (96–100%)	
DI water	
UV/Vis	Thermo Fisher Scientific NanoDrop 2000
Fluorescent DNA quantification	Thermo Qubit 3.0, Qubit dsDNA BR Assay Kit

*The SRE kit is recommended for the HiFi workflow. Please see the SRE Procedure & checklist for more information.

For all protocols

- Eppendorf DNA LoBind tubes (Eppendorf #022431021) are recommended for most library preparations.

© 2023 PacBio. All rights reserved. Research use only. Not for use in diagnostic procedures.

Introduction

The SRE XS and SRE XL kits can be used for rapid high-pass size selection of high molecular weight (HMW) DNA samples. This method can significantly enhance mean read length by progressively depleting short DNA up to 10 kb (SRE XS kit) or 40 kb (SRE XL kit) in length (Figure 1). The kits use a centrifugation procedure similar to standard ethanol precipitation techniques.

Note: If you are using the SRE kit, refer to the SRE Procedure & checklist. This Guide & overview is specific to SRE XS and SRE XL kits only. For the HiFi workflow, we recommend using the SRE kit on starting DNA (before shearing and library preparation).

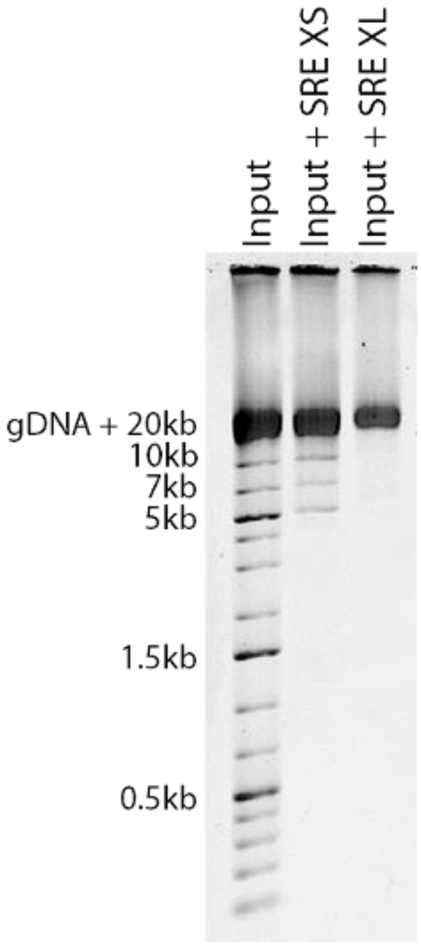

Figure 1. 1% Agarose gel separation of size-selected DNA with size cutoffs demonstrated using a spiked-in ladder (Thermo Scientific GeneRuler 1 kb Plus, #SM1334). Input is 50 ng/µL gDNA extracted from GM12878 cells using Nanobind® CBB kit + 20 ng/µL ladder.

© 2023 PacBio. All rights reserved. Research use only. Not for use in diagnostic procedures.

Sample information and expected performance

The choice of which kit to use should be based on the desired size selection performance and the quality of the input DNA as outlined in the table below. The stated recovery efficiencies will only be achieved when suitable quality input DNA is used at the appropriate Qubit DNA concentration.

Short Read Eliminator kits				
**Version**	**DNA depletion range**	**Qubit DNA input**	**Recovery efficiency**	**Sample notes**
SRE XS	Progressive depletion: <10 kb<br>Near complete depletion: <5 kb	25–150 ng/µL	50–90%	Suitable for sheared/fragmented DNA
SRE XL	Progressive depletion: <40 kb<br>Near complete depletion: <10 kb	50	40	Requires very high quality HMW DNA (>>48 kb)

The SRE XS kit should be used if the DNA sample is sheared/fragmented, has low concentration, or if very high recovery is needed. For this kit, the DNA concentration must be between 25–150 ng/µL.

The SRE XL kit should be used where there is very high quality HMW DNA and the majority of DNA is >> 48 kb. For this kit, the DNA concentration must be between 50–150 ng/µL. Using a lower quality DNA sample than suggested will result in lower-than-expected recovery efficiency. Using a higher than suggested DNA input concentration could impact size selection performance.

It is essential that DNA concentration is determined by a Qubit system or PicoGreen assay. Using concentrations derived solely from UV-Vis measurements will often result in low recovery, as the DNA concentration will be over-estimated due to RNA that may also be present in the sample.

The DNA sample should be in TE buffer (pH 8), the supplied Buffer EB, or water. If the sample buffer differs significantly or contains high levels of salt, the size selection properties and recoveries may be affected.

© 2023 PacBio. All rights reserved. Research use only. Not for use in diagnostic procedures.

HMW DNA size selection

Each SRE kit progressively depletes short DNA beginning at its upper cutoff, with depletion efficiency increasing as the DNA gets shorter.

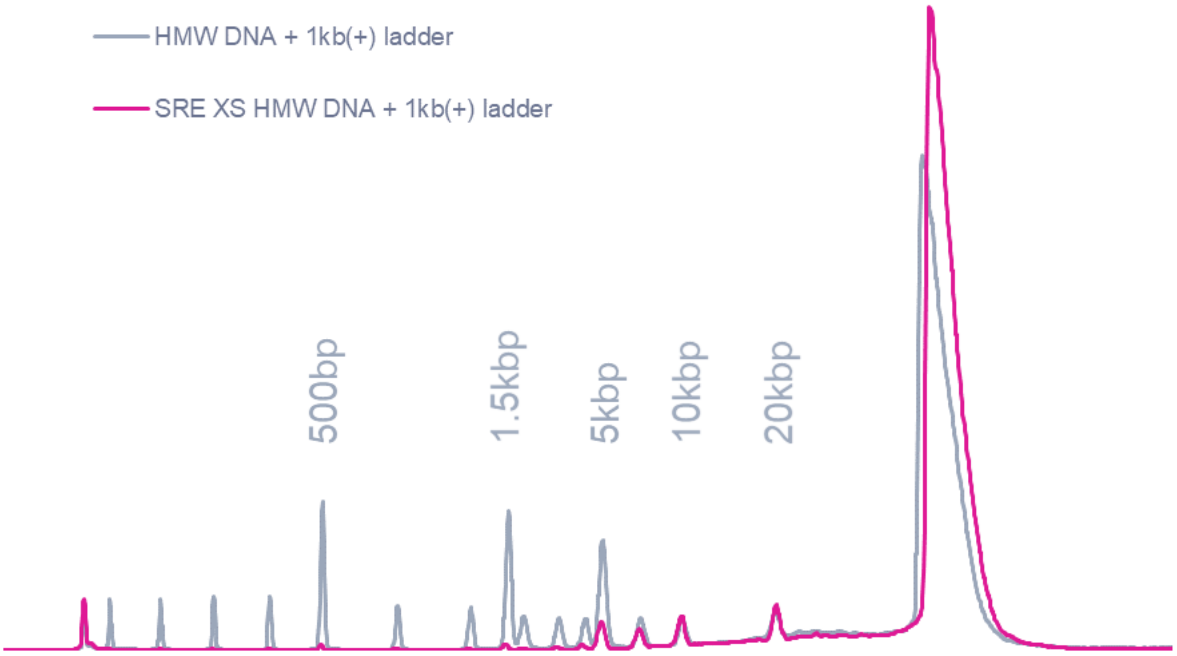

Figure 2 illustrates the performance of the Short Read Eliminator XS kit on HMW DNA spiked with 1kb+ ladder. DNA <5 kb in length is removed to trace levels as detected by gel or a Femto Pulse system.

© 2023 PacBio. All rights reserved. Research use only. Not for use in diagnostic procedures.

Sheared/fragmented DNA size selection (SRE XS kit only)

For sheared or fragmented DNA samples, only the SRE XS kit should be used. Use of the SRE XL kit on sheared/fragmented DNA samples will likely result in very low recovery efficiency.

Figure 3 shows size selection and recovery data from DNA samples that were sheared down to 10, 20, and 30 kb using a Diagenode Megaruptor 3 system. DNA input concentration down to 25 ng/µL can be used for all samples except the 10 kb sample, which requires 50 ng/µL to obtain suitable recovery.

The SRE XS kit can also be used on long PCR amplicons.

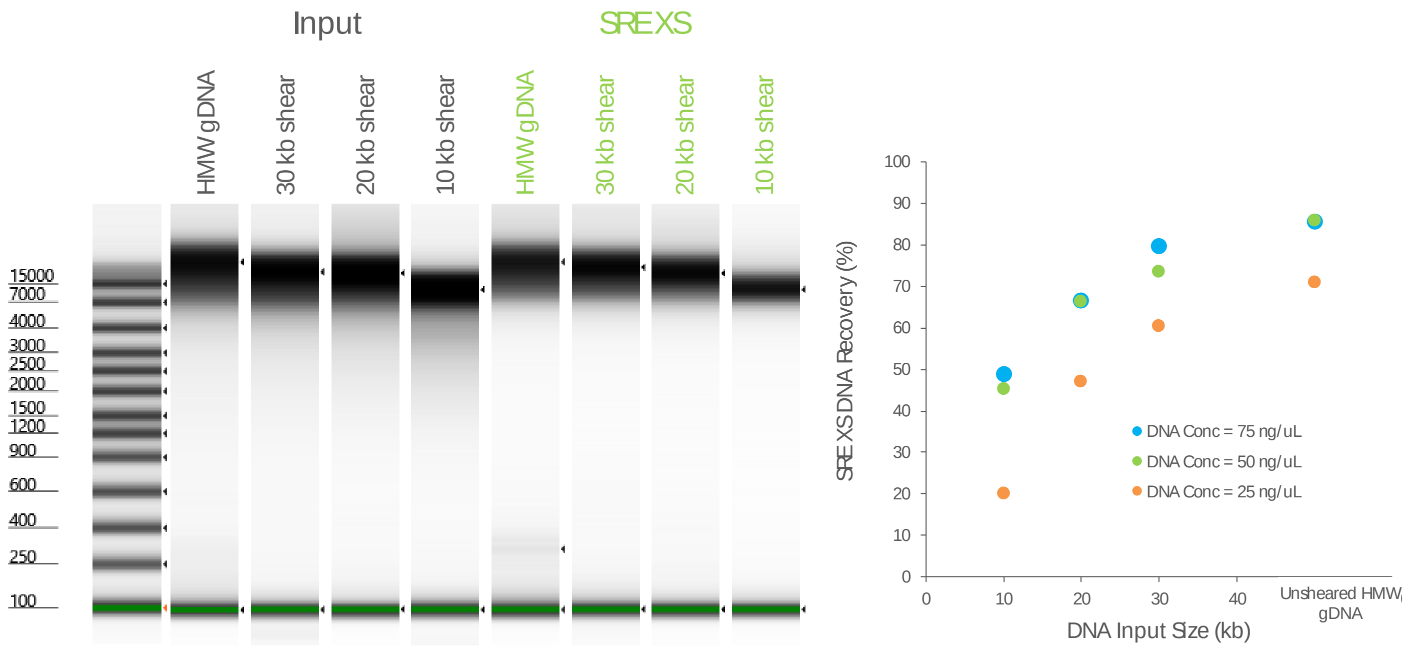

Figure 3 gDNA was extracted from GM12878 cells using the Nanobind CBB kit, diluted to 100 ng/µL, and sheared to 10, 20, or 30 kb using a Megaruptor 2 system. The samples were then size selected using the SRE XS kit and analyzed on an Agilent Tapestation 4200 system. Recovery efficiency decreases with DNA length but is >50% for all samples at input concentrations ≥50 ng/µL. Also, 25 ng/µL can be used for all samples except the 10 kb sample, unless low recovery efficiency can be tolerated.

© 2023 PacBio. All rights reserved. Research use only. Not for use in diagnostic procedures.

Workflow

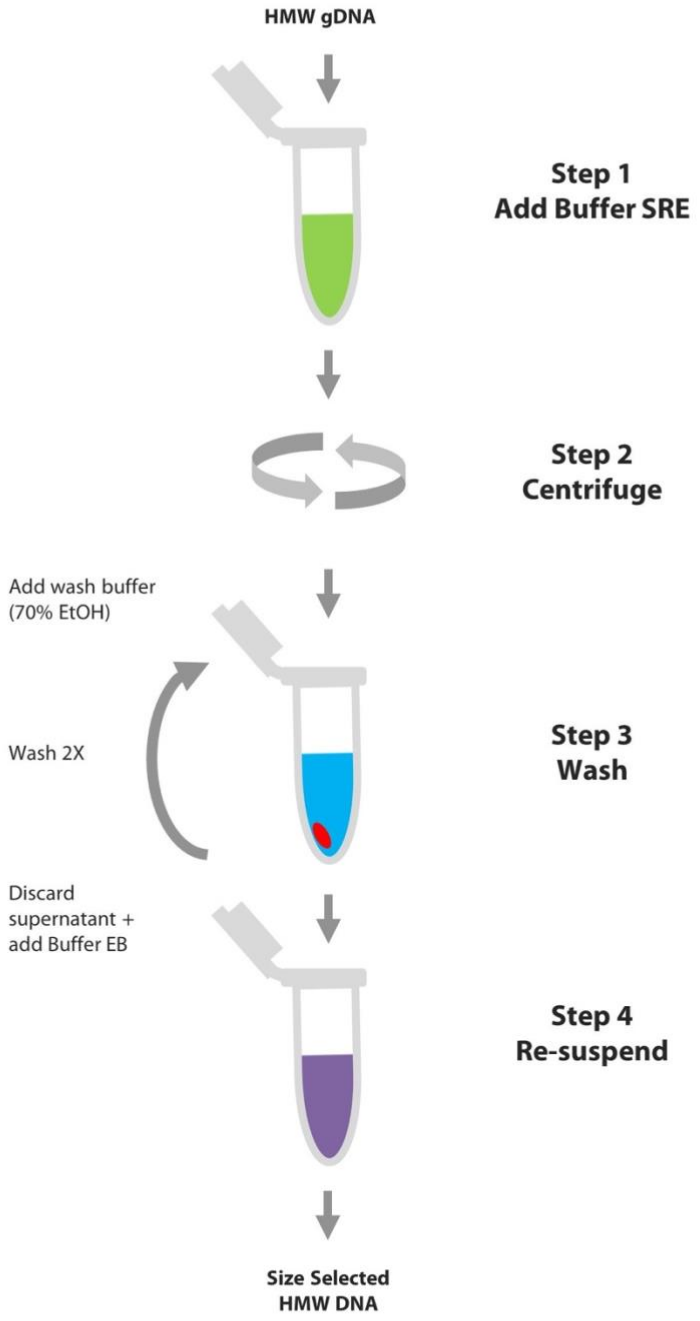

© 2023 PacBio. All rights reserved. Research use only. Not for use in diagnostic procedures.

Processing tips

Pipetting

Load the tube into a centrifuge with the hinge facing toward the outside of the rotor. This will help to avoid disturbing the pellet if it cannot be seen. After centrifugation, the DNA pellet will have formed on the bottom side of the microcentrifuge tube under the hinge region. Pipette on opposite side towards the thumb lip to avoid disturbing the pellet as shown in Figure 7.

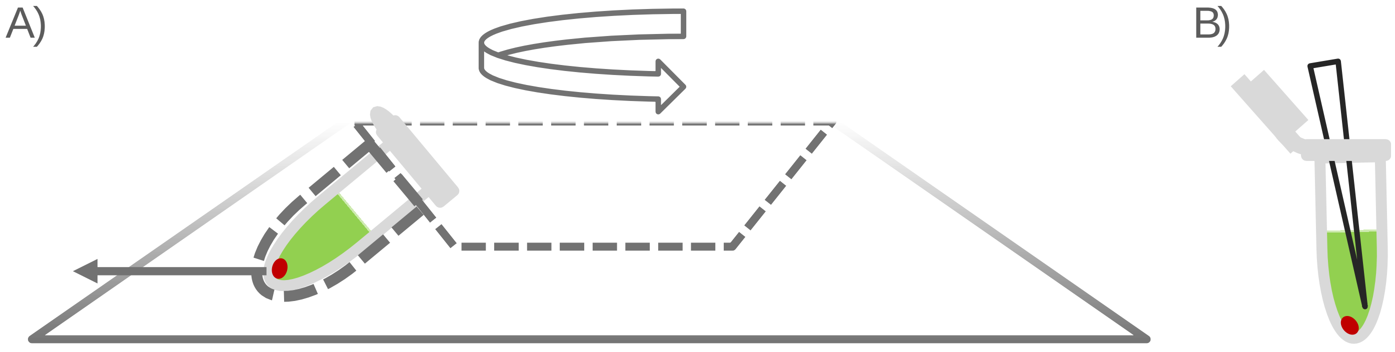

Figure 7. A) Note orientation of tube in centrifuge. Pellet will form on side of the tube facing outwards, in this case underneath the hinge region. B) Pipette from opposite side of tube on the thumb lip side to avoid disturbing pellet. Pellet may not be visible.

Heterogeneity and viscosity

Recovery efficiency and size selection performance of the Short Read Eliminator kits depend on the input DNA being homogeneous and fully in solution. HMW DNA can sometimes be difficult to re-solubilize after extraction and results in an inhomogeneous sample. Such samples will result in low yields and carry-over of short DNA if used with the Short Read Eliminator kits. If the HMW DNA sample is inhomogeneous or contains viscous jellies, we recommend needle shearing with 5-10X with a 26G needle and then allowing the DNA to rest at room temperature overnight before beginning size selection. Sample homogeneity can be evaluated by performing triplicate concentration measurements and verifying that the concentration CV is <20%.

© 2023 PacBio. All rights reserved. Research use only. Not for use in diagnostic procedures.

Size selection protocol for SRE XL

The following protocol details size selection of HMW DNA**. For HiFi sequencing, size selection should be performed on gDNA prior to shearing and library preparation.** The input HMW DNA should have length >48 kb and Qubit DNA concentration >50 ng/µL. **Always use wide bore pipettes.**

HMW gDNA

1. Adjust the DNA sample to a total volume of 60 µL and a Qubit DNA concentration of between 50–150 ng/µL. Pipette the sample into a 1.5 mL Eppendorf DNA LoBind tube.
   - This concentration MUST be measured using a Qubit dsDNA Broad Range Assay or equivalent.
   - Dilute sample using TE buffer (pH 8), Buffer EB, or water.
2. Add 60 µL of SRE XL to the sample. Mix thoroughly by gently tapping the tube or by gently pipetting up and down.
3. Load the tube into a centrifuge with the hinge facing toward the outside of the rotor.
4. Centrifuge at 10,000 x *g* for 30 mins at room temperature.
   - If using a centrifuge with temperature control (i.e., cooling function), turn this function off by setting the temperature to 29°C.
5. Carefully remove supernatant from tube without disturbing the DNA pellet. Place the pipette tip on the thumb lip side of the tube (see Figure 7).
   - The DNA pellet will have formed on the bottom of the tube under the hinge region.
6. Add 200 µL of the 70% EtOH wash solution to the tube and centrifuge at 10,000 x *g* for 2 mins at RT.
   - Do not tap or mix after adding 70% EtOH. Place the tube directly into the centrifuge.
7. Carefully remove the wash solution from the tube without disturbing the DNA pellet. Place the pipette tip on the thumb lip side of the tube (see Figure 7).
8. Add 50–100 µL of Buffer EB to the tube and incubate at room temperature for 20 minutes. Buffer volume may be adjusted to achieve desired concentration.
9. After incubation, gently tap the tube to ensure that the DNA is properly re-suspended and mixed.
10. Analyze the recovery and purity of the DNA using a NanoDrop and Qubit system.

Quick tip

Using concentrations derived from UV-Vis measurements without accounting for RNA concentrations will adversely affect yields.

Quick tip

The DNA pellet may not be visible. Placing the tube and pipetting in the directed orientations will prevent accidentally aspirating the DNA pellet.

Quick tip

Longer DNA can take more time to re-suspend. Heating to 50°C or eluting for more time can help increase recoveries.

© 2023 PacBio. All rights reserved. Research use only. Not for use in diagnostic procedures.

Size selection protocol for SRE XS

The following protocol details size selection of HMW gDNA or fragmented DNA. **For HiFi sequencing, size selection should be performed on gDNA prior to shearing and library preparation.** The Qubit DNA concentration should be >25 ng/μL.

HMW gDNA or sheared/fragmented DNA

1. Adjust the DNA sample to a total volume of 60 μL and a Qubit DNA concentration of between 25–150 ng/μL. Pipette the sample into a 1.5 mL Eppendorf DNA LoBind tube.
   - This concentration MUST be measured using a Qubit dsDNA Broad Range Assay or equivalent.
   - Dilute the sample using TE buffer (pH 8), Buffer EB, or water.
2. Add 60 μL of Buffer SRE XS to the sample. Mix thoroughly by gently tapping the tube or by gently pipetting up and down.
3. Load the tube into the centrifuge with the hinge facing toward the outside of the rotor.
4. Centrifuge at 10,000 x *g* for 30 mins at room temperature.
   - If using a centrifuge with temperature control (i.e., cooling function), turn this function off by setting the temperature to 29°C.
5. Carefully remove the supernatant from tube without disturbing the DNA pellet. Place the pipette tip on the thumb lip side of the tube (see Figure 7).
   - The DNA pellet will have formed on the bottom of the tube under the hinge region.
6. Add 200 μL of the 70% EtOH wash solution to tube and centrifuge at 10,000 x *g* for 2 mins at RT.
   - Do not tap or mix after adding 70% EtOH. Place tube directly into the centrifuge.
7. Carefully remove the wash solution from the tube without disturbing the DNA pellet. Place the pipette tip on the thumb lip side of the tube (see Figure 7).
8. Add 50–100 μL of Buffer EB to the tube and incubate at room temperature for 20 minutes. The buffer volume may be adjusted to achieve desired concentration.
9. After incubation, gently tap the tube to ensure that the DNA is properly re-suspended and mixed.
10. Analyze the recovery and purity of the DNA by using a NanoDrop and Qubit system.

Quick tip

Using concentrations derived from UV-Vis measurements without accounting for RNA concentrations will adversely affect yields.

Quick tip

The DNA pellet may not be visible. Placing the tube and pipetting in the directed orientations will prevent accidentally aspirating the DNA pellet.

Quick tip

Longer DNA can take more time to re-suspend. Heating to 50°C or eluting for more time can help increase recoveries.

© 2023 PacBio. All rights reserved. Research use only. Not for use in diagnostic procedures.

Troubleshooting guide

If recovery is poor:

- **Highly fragmented gDNA.** When using the SRE XL kit, recovery will be low if DNA is not HMW. Verify that a significant fraction of the input DNA is >48 kb by using PFGE or capillary electrophoresis.
- **Low input DNA concentration.** Recovery will be low if dsDNA concentration is <50 ng/µL. Verify the input gDNA concentration using Qubit dsDNA broad range assay or equivalent. Using concentrations derived solely from UV-Vis measurements will often result in low recovery as the estimated DNA concentration will not account for RNA that is also present in the solution. Try increasing concentration of input DNA up to the maximum of 150 ng/µL.
- **Incorrect centrifugation speed.**
- **Incorrect centrifugation temperature.** Recovery will be impacted if centrifugation is performed at low temperature (e.g., 4°C). Verify that the centrifuge is not cooling by turning off cooling or setting the temperature above ambient (e.g., 29°C).
- **Heterogeneous input sample.** If input sample is heterogeneous and contains fractions of DNA that are not fully solubilized, recovery will be affected. Verify homogeneity by pipetting to ensure that no viscous jellies exist in the sample. Homogeneity can also be determined by performing triplicate concentration measurements and verifying that the CV <20%. If the sample fails these tests, needle shear the input DNA 10X using a 26G needle or pipette mix 10X using a standard P200 pipette and allow to rest overnight at RT before proceeding.
- **Non-standard DNA buffer.** This method has only been tested using DNA in solubilized in TE buffer (pH 8), Buffer EB, or water. If the DNA sample contains high levels of contaminants or salts, recovery may be affected.
- **Handling error.** The DNA pellet is often invisible. If the pellet is disturbed during the wash steps, it is possible to accidentally aspirate it into the pipette tip. Ensure that proper care is taken with tube orientation during centrifuge and pipetting steps such that pipetting is always performed on the opposite side of the tube from the pellet.

If the cutoff seems too low/too high:

Non-standard DNA buffer. This method has only been tested using DNA in water, TE buffer, or Buffer EB. If the DNA sample contains high levels of contaminants or salts or compounds that affect DNA solubility/precipitation, size selection performance may be affected.

Revision history (description)	Version	Date
Initial release	01	July 2022
Minor updates throughout	02	December 2022
Moved information specific to the SRE kit to separate Procedure & checklist	03	March 2023

Research use only. Not for use in diagnostic procedures. © 2023 Pacific Biosciences of California, Inc. ("PacBio"). All rights reserved. Information in this document is subject to change without notice. PacBio assumes no responsibility for any errors or omissions in this document. Certain notices, terms, conditions and/or use restrictions may pertain to your use of PacBio products and/or third-party products. Refer to the applicable PacBio terms and conditions of sale and to the applicable license terms at pacb.com/license. Pacific Biosciences, the PacBio logo, PacBio, Circulomics, Omniome, SMRT, SMRTbell, Iso-Seq, Sequel, Nanobind, SBB, Revio, and Onso are trademarks of PacBio.

© 2023 PacBio. All rights reserved. Research use only. Not for use in diagnostic procedures.
