# Supplementary material for: Genomic DNA extraction optimization and validation for genome sequencing using the marine gastropod Kellet’s whelk
Source: PeerJ. 2023 Dec 6;11:e16510. doi: 10.7717/peerj.16510 (PMC10710129; doi:10.7717/peerj.16510)
Supplement: Supplemental Information 7 [file peerj-11-16510-s007.zip › Salting Out Protocol Checklist.pdf]

Salting Out Protocol Checklist

Preparation

- [ ] Wash hands, put on gloves and lab coat and wipe down lab bench with 70% ethanol
- [ ] Turn on water bath (set to 65°C) and place 1% SDS cell lysis reagent inside
- [ ] Fill cooler with ice and grab assigned 20 samples out of the freezer
- [ ] Clean the tissue homogenizer with 70% ethanol and wipe clean
- [ ] Grab the proteinase K and set out at room temp to begin thawing and grab the RNase A and place in ice tray
- [ ] Fill homogenizer wash beakers labeled with H2O and EtOH (ethanol) and fill with DI H2O and 70% ethanol
- [ ] Grab the 1% SDS cell lysis buffer out of the water bath (make sure its at 65°C, and cell lysis buffer is clear)
- [ ] Lay out 1% SDS cell lysis buffer, 0.5M EDTA, and Proteinase K for pipetting

Cell Lysis and DNA stabilization

***Create a Cell Lysis Working Solution (WS) (mix of 1% SDS cell lysis buffer, 0.5M EDTA, and Proteinase K) using a 15mL Falcon tube

- [ ] Add **7000 µl of 1% SDS cell lysis buffer** to the WS (one tip) (1,000µl, 7 times, if you lose track, there's a 7mL marker on the falcon tube)
- [ ] Add **840 µl of 0.5M EDTA** to the WS (one tip)
- [ ] Add **400 µl of Proteinase K** to WS (one tip), mix by vortexing lightly (don't want too many bubbles!)
- [ ] Add **412 µl of Cell Lysis WS** to each sample (one tip)
- [ ] Bring **Proteinase K back to freezer**
- [ ] Begin grinding the tissue samples with the tissue homogenizer (~10x each) making sure to clean with water - wipe down, then clean with water and ethanol - wipe down between each sample
- [ ] Place all samples in the water bath rack and mix by vortexing each sample for ~5 seconds **(make sure the lids are on tight!)**
- [ ] Place samples in the 65°C water bath and set a timer for 1 hour
- [ ] Set incubator to 37°C
- [ ] Label 20 test tubes with the sample number (letter-number) on top and side

- [ ] Vortex samples for ~3 seconds and place back in water bath
- [ ] Label 20 test tubes with sample number (letter-number) on top and full label on side (location-A/R-year-number) and place in separate test tube rack for later
- [ ] Vortex samples for 3 seconds and place back in water bath
- [ ] Wait until timer goes off and bring samples back to lab bench
- [ ] Add **25 µl RNase A** to each sample (new tip)
- [ ] Place RNase A back on ice and mix by inversion 10 times
- [ ] Place samples in 37°C incubator and set timer for 10 minutes
- [ ] Bring the **RNase A back to the freezer**
- [ ] Turn on the large centrifuge and set it to 4°C
- [ ] Wait until timer goes off

Remove proteins and cellular debris

- [ ] [ ] Add **150 µl 7.5M Ammonium Acetate** to each sample (one tip), for the second round add **60 µl 7.5M Ammonium Acetate**

- [ ] [ ] Mix by vortexing for 5 seconds and place samples in 4ºC centrifuge and incubate for 5 minutes (DO NOT PUSH START) (can use fridge if centrifuge is occupied)

- [ ] [ ] Set centrifuge to **12,000xg for 10 minutes at 4ºC** and make sure centrifuge is balanced - press start

- [ ] [ ] Set timer for 10 minutes

- [ ] Bring samples back to bench, **place on ice** and transfer supernatant (560 µl) to the test tubes labeled with just the sample letter-number (**DO NOT TRANSFER ANY MUCUS OR CELL DEBRIS**) (new tip)

- [ ] Repeat 5 preceding steps and transfer supernatant (620 µl) to test tubes with full labels (**DO NOT TRANSFER ANY MUCUS OR CELL DEBRIS**)

Precipitation of DNA

- [ ] Move the 70% Ethanol on ice

- [ ] Add **620 µl of 100% Isopropanol** to each test tube (one tip)

- [ ] Invert samples **50 times** gently and place in centrifuge

- [ ] Set centrifuge to **8000xg for 5 minutes at 4ºC**, place samples in and balance - press start

- [ ] Carefully discard the supernatant by dumping out the isopropanol **(DO NOT LET DNA PELLET SLIDE OUT**)

Washing DNA

☐ ☐ Add **400 µl of 70% Ethanol** to each DNA pellet (one tip)

☐ ☐ Invert the tubes 3 times to wash the DNA pellet and centrifuge the samples at **4ºC at 16,000xg for 5 minutes** (caps on tight!)

☐ ☐ Dump out Ethanol carefully **(DO NOT LET DNA PELLET SLIDE OUT)**

☐ Repeat previous three steps - remove any excess ethanol with **100 µl pipette** (new tip)

☐ Place samples in speedvac with caps open at 30ºC **(don't touch inside of caps)**

☐ Press start and set a timer for 4 minutes

☐ Check each sample to make sure all ethanol has evaporated (if there is still ethanol or any liquid dry for another minute in speedvac)

Rehydration of DNA

☐ Add **100 µl of TE buffer** to each sample (one tip)

☐ Place test tube rack in the fridge overnight

☐ Make sure all equipment is turned off (water bath, centrifuge, incubator, and speedvac) and clean up lab bench

THE END
