# Supplementary material for: Genomic DNA extraction optimization and validation for genome sequencing using the marine gastropod Kellet’s whelk
Source: PeerJ. 2023 Dec 6;11:e16510. doi: 10.7717/peerj.16510 (PMC10710129; doi:10.7717/peerj.16510)
Supplement: Supplemental Information 7 [file peerj-11-16510-s007.zip › NatesPlates_protocol_v2-PATENTPENDING.pdf]

PROTOCOL FOR USE OF "NATE'S PLATES" TAGGING AND NORMALIZATION KITS

I. PCR 2

Thaw Qiagen Plus master mix at RT.

Dilute PCR1 product 1:20 using nuclease free water or TE.

Remove and discard adhesive seal on one of Nate's Plates. Transfer 2 µL diluted PCR1 product to Nate's Plate.

Mix Qiagen Plus MM and centrifuge briefly. Add 2 µL Qiagen Plus MM to each well.

PCR cycling conditions:

Step	Temp. (°C)	Time	Cycles
Hot Start	95	15:00	1
Denaturation	94	0:30	
Annealing	57	0:30	2
Extension	72	2:00	
Denaturation	94	0:30	18
Extension	72	0:45	
Final Extension	72	2:00	1
Hold	4	∞	1

II. Sample Normalization

a. Bind

Pool samples from each well in **Nate's Plate** and collect in tube; label tube as POOL. Add 500 µL of **Nate's Plates Bead Buffer** (2X) to POOL tube. Vortex the tube of Streptavidin beads and ensure they are adequately homogenized. Carefully transfer 1 µL strep beads to POOL tube. Vortex and incubate POOL for 15 minutes at room temperature, inverting tube occasionally.

Place POOL tube on magnetic rack and incubate for 3 minutes. Remove and discard supernatant, being carefµL to not disturb the bead pellet.

***!!Note:* The bead pellet will be very small and difficult to see!**

(similar to what a wisp of dirt would look like if stuck to the inside of the tube).

Wash twice with 1 ml **Nate's Plates Wash Buffer** (1X) while still on magnetic rack. Discard supernatant.

Resusupend beads in 20 µL sterile nuclease-free water.

PATENT PENDING

II. Sample Normalization (continued)

b. Release

Thaw components at room temperature. Mix well and centrifuge briefly.

Pipette 10 µL resuspended beads into well of a strip tube or plate for thermal cycling.

**Component**	**x1**
Qiagen Plus MM (2X)	20.00
10X Bead Release primers	4.00
Nuclease-free water	6.00
Resuspended beads	10.00
Total Volume (µL)	40.00

Optional: The remaining 10 µL of resuspended beads can be stored at -20 to 4°C if desired.

PCR cycling conditions:

Step	Temp. (°C)	Time	Cycles
Hot Start	95	15:00	1
Denaturation	94	0:30	
Annealing	60	0:30	6
Extension	72	0:30	
Final Extension	72	2:00	1
Hold	4	∞	1

III. Bead Size Selection

!!Note: When discarding supernatant in the following steps, visually inspect pipette tip to ensure you are not discarding magnetic beads!

Transfer PCR product to a new tube and place on magnetic rack. Let sit for three minutes.

Transfer 25 µL cleared supernatant to new tube. Add 15 µL SPRI beads; mix well and incubate at room temperature for 5 minutes.

Move tube to magnetic rack and let sit for three minutes. Transfer supernatant to a new tube and add 15 µL SPRI beads; mix well and incubate at room temperature for 5 minutes.

Move tube to magnetic rack and let sit for three minutes. Remove and discard supernatant. While on magnetic rack, add 200 µL of 75% EtOH (freshly prepared) to tube; incubate for 30 seconds and discard. Repeat wash step.

Remove tube from magnetic rack and incubate for 5-10 minutes with open lid at room temperature. If there is residual liquid after 10 minutes, carefully remove with a pipette tip and discard. Elute with 15 µL 1X TE buffer. Place on magnetic stand and collect cleared supernatant in new tube. Add 1.5 µL EB with 1% Tween-20.

IV. Proceed to qPCR

PATENT PENDING
