# Supplementary material for: Genomic DNA extraction optimization and validation for genome sequencing using the marine gastropod Kellet’s whelk
Source: PeerJ. 2023 Dec 6;11:e16510. doi: 10.7717/peerj.16510 (PMC10710129; doi:10.7717/peerj.16510)
Supplement: Supplemental Information 7 [file peerj-11-16510-s007.zip › GTseq Library Prep Protocol.pdf]

1. Exo-SAP Treat gDNA

(optional: improves results with sheared gDNA)

Thaw components at room temperature.
Mix well and centrifuge briefly; place Exo1 enzyme on ice.

Component	0.00
Exonuclease 1 Buffer	1.30
Nuclease Free H_2O	1.25
SAP	0.50
Exonuclease 1	0.50
gDNA	10.00
Total Volume	13.00

2. PCR 1

Thaw components at room temperature.
Mix well and centrifuge briefly.
Pipette 2 µl gDNA** into PCR plate. Add 5 ul PCR cocktail to each sample.

Component	x1
Qiagen Plus MM (2X)	3.50
0.25 µM GT-seq primers	1.50
gDNA **	2.00
Total Volume	7.00

*(** or Exo-SAP treated gDNA)*

PCR cycling conditions:

Step	Temp. (°C)	Time	Cycles
Hot Start	95	15:00	1
Denaturation	95	0:30	
Annealing	57	0:30	5
Extension	72	2:00	
Denaturation	95	0:30	
Annealing	65	0:30	10
Extension	72	0:30	
Hold	4	∞	1

Slow cool: 5% ramp rate (~ 0.1-0.3 degrees/sec)

Dilute PCR1 1:20 by adding 133 ul Nuclease-free H_2O

3. PCR 2

Thaw components at room temperature.
Mix well and centrifuge briefly.

Component	x1
Qiagen Plus MM (2X)	5.00
10 µM i7 index	1.00
10 µM i5 index	1.00
Diluted PCR1	3.00
Total Volume	10.00

PCR cycling conditions:

Step	Temp. (°C)	Time	Cycles
Hot Start	95	15:00	1
Denaturation	95	0:10	
Annealing	65	0:30	10
Extension	72	0:30	
Final Extension	72	5:00	1
Hold	4	∞	1

4. Sample Normalization

Transfer PCR 2 product to a CharmBiotech Just-A-Plate. Add 10 µl Binding Buffer to each well; seal, vortex and centrifuge. Incubate at room temperature for 1 hour.

Discard all liquid from plate but be careful not to scrape wells of normalization plate. Add 50 µl Wash Buffer to each well. Mix and discard. Invert plate on paper towel to remove as much remaining wash buffer as possible.

Incubate for 5 minutes at 55C or at room temp for 15 minutes to ensure residual ethanol has evaporated.

Add 20 µl Elution Buffer to each well. Seal, vortex and centrifuge. Incubate at room temperature for 5 minutes. Pool 10 µl from each well in a microcentrifuge tube.

5. Bead Size Selection

Combine in new tube 50 ul pooled normalized library and 25 ul SPRI beads; mix well and incubate for 5 minutes at room temp.
Move tube to magnetic rack and let sit for three minutes.
Transfer cleared supernatant to new tube. Add 35 ul SPRI beads; mix well and incubate at room temperature for 5 minutes.
Move tube to magnetic rack and let sit for three minutes; Remove supernatant and discard while being careful not to discard any beads.
While on magnetic rack, add 200 ul of 70% EtOH (freshly prepared) to tube; incubate for 30 seconds and discard.
Repeat wash step.
Remove tube from magnetic rack and incubate for 10 minutes with open lid at room temperature.
Elute with 15 ul 1X TE buffer. Place on magnetic stand and collect cleared supernatant in new tube. Add 1.5 ul EB with 1% Tween-20.

6. qPCR

In a new tube, prepare a 1:1000 dilution of your library using 1X TE buffer with 0.1% Tween-20; perform a serial dilution for a final dilution series of 1:1000, 1:2000, 1:4000 & 1:8000.

7. Run in duplicate using your favorite flavor of qPCR for quantitation of NGS libraries.
