# Supplementary material for: Genomic DNA extraction optimization and validation for genome sequencing using the marine gastropod Kellet’s whelk
Source: PeerJ. 2023 Dec 6;11:e16510. doi: 10.7717/peerj.16510 (PMC10710129; doi:10.7717/peerj.16510)
Supplement: Supplemental Information 7 [file peerj-11-16510-s007.zip › Protocol ezRAD whelks.pdf]

Step 1: Digest (page 16 protocol)

Digest reagents	Volume µl (per sample)
gDNA	25
CutSmart Buffer	5
HPLC grade H_2O	18
MboI	1
Sau3AI	1
Total volume	50

Thermocycler

(18 hours at 37°C, 20mins at 65°C, Hold at 15°C)

Step 2: Post-digest clean-up and check (Bead Clean)

Step 3: Kapa library prep (page 24 protocol)

1) End-repair and A-Tailing-Kapa

Components	Volume µl (per sample)
DNA	25
End-Repair & A-Tailing buffer	3.5
End-Repair & A-Tailing Enzyme mix	1.5
Total volume	30

Thermocycler

(30mins at 20°C, 30mins at 65°C, Hold at 4°C)

2) Adapter Ligation-Kapa (page 25 protocol)

Components	Volume µl (per sample)
DNA	15
HPLC grade H_2O	1.25
Ligation buffer	7.5
DNA ligase	2.5
Stubby adapter	1.25
Total volume	27.5

Thermocycler

(2hours at 20°C, Hold at 4°C)

3) Post ligation clean-up-Kapa (Bead clean)

4) Library amplification-Kapa (page 28 protocol)

Components	Volume µl (per sample)
DNA	10
2X KAPA HiFi HotStart Ready Mix	12.5
10X Library Amplification Primer Mix	2.5
Total volume	25

Thermocycler

45 secs at 98°C, (15 secs at 98°C, 30 secs at 60°C, 30 secs at 72°C) for X cycles, 1 min at 72°C, Hold at 4°C.
