# Supplementary material for: Genomic DNA extraction optimization and validation for genome sequencing using the marine gastropod Kellet’s whelk
Source: PeerJ. 2023 Dec 6;11:e16510. doi: 10.7717/peerj.16510 (PMC10710129; doi:10.7717/peerj.16510)
Supplement: Supplemental Information 7 [file peerj-11-16510-s007.zip › ToBo lab ezRAD Protocol_v3.2.pdf]

(Revised December 2016)

ToBo lab EzRAD Protocol-v3.2
Illumina & Kapa kits
Half reaction volumes

Introduction

One of the hardest things with the RADSeq protocols is knowing how much you "can get away with" and still get good data! This protocol will go through every step to try and help you get genomic data from non-model organisms. Determining from gels what to do/how to salvage your samples and determining how much DNA you really have to have to get it through the Illumina or Kapa kits and still pass the quality control steps at the end and get good data. Our test species were cnidarians, which are notoriously difficult to work with, we've also worked with sponges, echinoderms, crustaceans, molluscs, fish etc. So we hope this protocol is useful to many people who work on a range of organisms.

What's possible?

Each sequencing system is different, but the library output is the same. For sequencing we currently use the MiSeq, which can run 1 flow cell with 2 lanes, one of which is a control lane. On the remaining lane we run **8 libraries** to maximize coverage and reduce costs. Primarily we choose to use the MiSeq because with the current reagent kits it can sequence 300bp paired-end reads (i.e. 600bp total), which is considerably longer than the HiSeq, which only sequences 150bp paired end reads. However, the HiSeq does have its benefits in that you can load more samples per lane and get considerably more reads per library, you pick what you need/want based on your question.

One of the main differences between ezRAD and other RADSeq techniques is that you can end up not only with stacks (vertical coverage), which can be used to call SNPs, but also considerable horizontal coverage. This horizontal coverage means that we can get exceptionally long contigs, resulting often in the resolution of a large percentage, if not all, of the mitochondrial genome amongst other regions (e.g. histones, ribosomes). These long contigs also mean we can identify them and their location in the genome, making ezRAD a very versatile technique.

Almost anything can be successfully made into a library. With ezRAD each library can either consist of one individual or a pool of individuals. The latter requires more work, but the amount of DNA required is less per extraction. There are many kits on the market designed to generate libraries. Illumina's two main DNA kits include a **PCR-free** one which requires **1µg of input DNA** and the **Nano kit**, which only need **100-200ng.** Both kits come in low-throughput (24 individual indices) and high-throughput (96 dual indices). Alternatively, there is also the **Kapa Hyper Prep kit**, which only needs **1ng input DNA**, is cheaper and quicker than the Illumina kits; however, it does require that you buy the adapters and beads separately; more on this later.

I've tried to write this protocol so that almost anyone can make libraries, but I also didn't want it to be a tome, so this protocol is aimed at people with at least some molecular bench work experience.

Table of Contents

Step 1: Extractions ... 4

Step 2: Are your extractions ok? ... 5

Step 3: DNA quantitation ... 7

Step 4: Pooling samples (not necessary if doing single libraries) ... 10

Step 5: Choose your library preparation kit ... 11

Step 6: Concentrating your DNA ... 12

Step 7: Bead clean-up ... 13

Step 8: Digest ... 16

Step 9: Post-digest clean-up and checks ... 17

Step 10: Make libraries! ... 18

Illumina TruSeq DNA library protocol ... 18

1) End-repair-Illumina ... 19

2) Size selection-Illumina ... 19

3) Adenylate 3' Ends (A-tailing) -Illumina ... 20

4) Adapter ligation-Illumina ... 21

5) Post ligation clean-up-Illumina ... 22

6) Enrich DNA fragments- Nano kit only-Illumina ... 23

7) Post-amplification clean-up-Illumina ... 23

KAPA Hyper Prep DNA library protocol ... 24

1) End-repair and A-tailing-Kapa ... 24

2) Adapter ligation-Kapa ... 25

3) Post ligation clean-up-Kapa ... 26

4) Size selection (for 350-700bp insert) -Kapa ... 26

5) Library amplification-Kapa ... 27

6) Post-amplification clean-up-Kapa *29*

Step 11: Validate your libraries 30

Appendix 1: Setting up a lab to do ezRAD 31

Appendix 2: Removed from this protocol, but still in old versions 33

Appendix 3: Illumina adapter sequences 34

Appendix 4: Illumina experienced user version 35

Appendix 5: KAPA experienced user version 36

Step 1: Extractions

The collection and storage of samples is very important in determining how easy it is to get your samples ready to put through the Illumina/Kapa kit. For most species you should preferentially store samples in DMSO or RNAlater (see homebrew recipe here). Ethanol and even dried samples can work but the DNA is often degraded and involves extra clean-up steps. The goal of the extractions is to get as much high quality DNA as you can in 25-44 µl of HPLC grade water. These volumes relate to the input volumes for the next digestion step. If you want to avoid any PCR steps in the kits then you need to get to get at least 1ug of good quality DNA. However, unlike regular PCR's the primers are designed for the adapters you attach so there is no sequence bias at this amplification step. So having low final quantities of DNA is fine, as long as it's not all low quality (i.e. its quality over quantity). The Illumina Nano kit, only requires 100ng and the Kapa Hyper Prep kit can seemingly make libraries with as little as 1ng, so low DNA isn't an issue like it used to be with the old kits.

The Qiagen or Omega tissue and genomic DNA extraction kits work well, but any extraction method that yields high quality clean extractions will work (i.e. not the Hotshot method).

Notes:

1. For corals, to increase the yield, consider doubling the amount of tissue and reagents up until the sample is put through the filter. These larger fragments are around 0.5-1cm^3 and if necessary the skeleton should be crushed.
2. Fresh coral samples can yield degraded DNA because of their mucous layer so an optional step is to put them in DMSO/ETOH at least overnight before extracting.
3. If your samples are in ethanol select the piece you wish to extract and leave them on a piece of tissue for 10-15 minutes to allow the ethanol to evaporate.
4. Do the optional RNase A step in the protocol. The RNase A final concentration is at 100 mg/ml and is aliquoted into separate Microcentrifuges and stored in the large freezer on the right on the top shelf.
5. **Elute the DNA in water** not the elution buffer so that you can concentrate the DNA using the SpeedVac instead of having to do a precipitation, which can result in the loss of a lot of your DNA.
6. Warm this elution water to 55-70°C as you would for the buffer (this is in the Omega kit instructions, but heat the water even if you are using the Qiagen kit). Leave the water on the spin column for 2 mins before spinning.
7. For all extractions **do 3 or 4 elutions** (stored in separate tubes):

1st elution of 50 µl. This is the most important elution. It often holds a lot of low quality DNA and additional contaminants, which are now eliminated from you subsequent extractions. Alternatively if you're lucky it could contain all of your high quality DNA, which is then highly concentrated enough that it can go straight to the digestion step without the need for using the Speed-vac. This elution is specifically 50µl because the digestion requires 25-44µl and if the sample concentration is high enough the additional µl gives you enough volume to run a gel and quantify the sample and still go straight to the digestion. Also if this elution needs to be discarded (because it has all of the low quality DNA) you are not throwing out much of your sample.

2nd elution of 50 µl. The volume of this elution is also low because it can also have enough good quality DNA to go straight into the digestion without using the SpeedVac and may also have the added benefit of having some of the low quality DNA removed in the first elution.

3rd and 4th elutions of 50µl each, stored together to give you 100 µl total.

Once you know what works for your species you can reduce the number of elutions if necessary. Preferably you will get enough DNA to make a backup, just in case the first library prep doesn't work.

Step 2: Are your extractions ok?

First things first there are 2 exceptionally important things to do throughout the protocol:

1. **Make sure that before any step, where your DNA has been sitting in a fridge or freezer that you thoroughly mix and briefly spin down the sample. I can't stress how important this is! It will affect your gels, quantitation, pooling etc.**
2. **Watch your pipetting, literally! This is especially important when using the multiwall pipettes as some tips can loosen. There are often markers on pipette tips, use them. Get to know what 1 or 2µl looks like, it will improve your lab work.**

Run a gel on your extractions to see the quality of your DNA (here I will assume that most people know how to run a gel, if not someone in the lab will).

From your gel you determine what to do next. Successful libraries were produced from the vast majority of samples in the gel below (Figure 1), including number 1-6, 8-10 and 12, which clearly have variable quantities and qualities. Sample 7 and 11, once digested and cleaned only had ≤ 200ng, so these would be more suitable for the Illumina Nano kit or Kapa kit. So you can see that almost every sample is usable. However, it is still best to aim for high quality extractions as this may affect the quality of data you get from the sequencer.

If you have high molecular weight bands (e.g. samples: 2, 9 and 10) that's perfect and if you have a smear (e.g. samples: 1, 3, 4, 8, 11, 12) then that is workable, you should now quantify your DNA (see below). If you have a bright low band i.e. a large amount of low quality DNA and a smear (e.g. samples: 5 and 6) then the samples can be cleaned with the AMPure beads before quantifying. If you only have a bright low band on the gel (e.g. sample 7 or worse) then if you clean the samples first (and quantify it) then this may work at least with the Kapa kit, which only needs 1ng DNA, alternatively try re-extracting.

Note: We have noticed that with more degraded samples (a smear, rather than a high band) that you get more horizontal coverage, as the DNA is already at the target size before digestion. So if you're aim is to get the mitochondrial genome, rather than stacks, then you might want to aim to use slightly degraded DNA.

For cleaning low quality samples try a 1:0.6 ratio of DNA:beads.

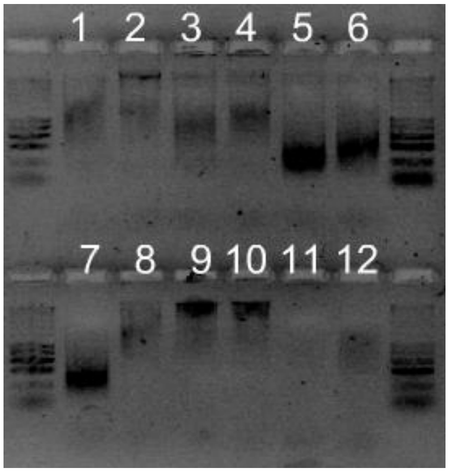

Figure 1. 1% agarose gel ran on 12 scleractinian coral extractions using HyperLadder 1 and GelStar nucleic acid stain.

Below is a table of the final DNA concentrations for each sample put through the Illumina TruSeq kit. All the samples worked and passed the final quality controls, so again it's clear that the kit is somewhat flexible to the quantity of input DNA as well as the quality.

Table 1. The total amount of DNA per sample put into the Illumina protocol after extraction, digest and clean-up. All of these samples made successful libraries passing both quality control (QC) steps (qPCR and bioanalyzer).

Sample	Total DNA (ng)
**1**	500
**2**	700
**3**	700
**4**	600
**5**	430
**6**	750
**8**	800
**9**	800
**10**	800
**12**	1000

Step 3: DNA quantitation

For the greatest accuracy we use AccuClear Ultra high sensitivity dsDNA quantitation kit with 1 or 7 DNA standards (plus one blank (0ng/µl) standard making 8 standards in total).

Note: the 1 standard kit is cheaper but you have to prepare the other 7 standards through serial dilutions (instructions are provided with the kit) but this takes time as "the two lowest concentration DNA dilutions (H, I) should be prepared fresh on the day of assay. The other DNA dilutions (B-G) can be stored at 4ºC for at least 6 months with the addition of 2 mM final concentration sodium azide."-Taken from the AccuClear protocol.

The NanoDrop is not good enough to quantify extractions. It may provide sufficient accuracy when samples have been cleaned, but the microplate AccuClear assay can be used throughout the protocol because the detection of dsDNA is not affected by single stranded RNA or DNA or common contaminants such as proteins, salts and organic solvents. Also this kit is designed to detect DNA concentration accurately from 0.03 to 250ng per assay (well), which should cover most samples.

How to setup the AccuClear quantitation:

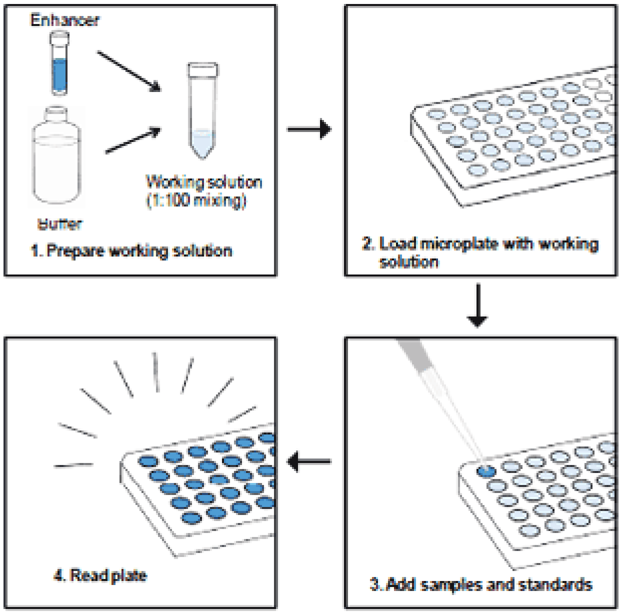

Figure 2. AccuClear can quantify a large number of dsDNA samples in four easy steps. Image taken from the Biotium website, see link here.

Equipment/reagents required:

Note: **Take all reagents out of the fridge 30 minutes before using!**

- AccuClear dye- because it is stored in DMSO it can form crystals, if so shake it till they're gone.
- AccuClear buffer- make sure this is at room temperature before beginning
- DNA Standards (if there is only 1 you need to serially dilute it to make 8, see below)
- Multiwell pipette
- If available a 10ml or 20ml pipette (not essential, but makes it easier)
- Black flat bottom 96-well plate
- Reagent trough/reservoir
- Timer
- Multiplate reader
- USB Flash drive
- Excel

Method for DNA quantitation:

1. Let the reagents warm up to room temperature for at least 30 minutes! This takes more/less time depending on the volume in the bottle.
2. Turn on the microplate reader (in the Core lab), open the software **SoftMax Pro 4.8**.
3. Prepare a working solution immediately before use in a reagent trough/reservoir (**See calculations below or Table 2**). A trough is used because the mixed solution will be transferred to a 96-well plate using a multiwall pipette. Remember that you have to run 2 rows of standards (16 in total) in addition to your samples! But once the standards appear consistent for a given kit, you can think about reducing it to 1 row of standards per plate if necessary.
4. **Add 10 µl of DNA** into the wells. If you need to be conservative with the amount of DNA you use, you can also put in **9 µl of H_2O** to each well and add your **1 µl DNA to make a total of 10 µl in each well**.
5. **Add 10 µl of each DNA standard** (0, 0.003, 0.01, 0.03, 0.1, 0.3, 1, 3, 10 and 25 ng/µl) to the last 2 rows (or last row if you only want 1 row of standards). The 0ng/µl should be in well A (the top of the plate). Mix (flick) the standards for about 1 minute before putting them in the plate (be careful that the lid doesn't pop open) and briefly spinning them down.
6. **Add 200 µl of the working solution** (dye and buffer in the reagent reservoir) to each well of a BLACK 96-well plate using the multiwall pipette (See **Table 2** below for volumes of the dye and buffer to mix)
7. Incubate the plate at room temperature for 3-5 minutes in the dark (in a drawer is fine). Grab a USB Flash drive.
8. After 3-5 minutes get you're samples and put them in the open drawer of the microplate reader.
9. In SoftMax Pro 4.8 **go to Setup** and make sure the fluorescence measurements are set to **468 nm excitation and 507 nm emission** (these are not the default settings).
10. Set the precision up to the highest possibly number of reads per well (which is 30 for our microplate reader)
11. If you're not running a whole plate select the number of columns you're using.
12. Click OK
13. **Press 'read'**.
14. **Save** the file to a folder in 'users' on the desktop.
15. Also **Export** the file from the microplate-reader program as a txt file onto your pen drive.
16. Close the drawer of the microplate reader (a button on the machine), switch it off and put the cover back.

Note: **You don't need to add any samples to your calculations to account for pipetting errors**; this is already accounted for in the calculations.

Table 2 The amount of buffer (ml) and dye (µl) to mix together to make a working solution for n number of wells for the Biotium dsDNA quantitation kit (AccuClear or AccuBlue).

# of wells	Buffer (ml)	Dye (µl)	# of wells	Buffer (ml)	Dye (µl)	# of wells	Buffer (ml)	Dye (µl)	# of wells	Buffer (ml)	Dye (µl)
**1**	0.208	2.1	**25**	5.20	52.1	**49**	10.19	102.1	**73**	15.18	152.1
**2**	0.416	4.2	**26**	5.41	54.2	**50**	10.40	104.2	**74**	15.39	154.1
**3**	0.624	6.3	**27**	5.62	56.2	**51**	10.61	106.2	**75**	15.60	156.2
**4**	0.832	8.3	**28**	5.82	58.3	**52**	10.82	108.3	**76**	15.81	158.3
**5**	1.04	10.4	**29**	6.03	60.4	**53**	11.02	110.4	**77**	16.02	160.4
**6**	1.25	12.50	**30**	6.24	62.5	**54**	11.23	112.5	**78**	16.22	162.5
**7**	1.46	14.6	**31**	6.45	64.6	**55**	11.44	114.6	**79**	16.43	164.6
**8**	1.66	16.7	**32**	6.66	66.7	**56**	11.65	116.7	**80**	16.64	166.6
**9**	1.87	18.8	**33**	6.86	68.7	**57**	11.86	118.7	**81**	16.85	168.7
**10**	2.08	20.8	**34**	7.07	70.8	**58**	12.06	120.8	**82**	17.06	170.8
**11**	2.29	22.9	**35**	7.28	72.9	**59**	12.27	122.9	**83**	17.26	172.9
**12**	2.50	25.0	**36**	7.49	75.0	**60**	12.48	125.0	**84**	17.47	175.0
**13**	2.70	27.1	**37**	7.70	77.1	**61**	12.69	127.1	**85**	17.68	177.1
**14**	2.91	29.2	**38**	7.90	79.2	**62**	12.90	129.2	**86**	17.89	179.1
**15**	3.12	31.3	**39**	8.11	81.2	**63**	13.10	131.2	**87**	18.10	181.2
**16**	3.33	33.3	**40**	8.32	83.3	**64**	13.31	133.3	**88**	18.30	183.3
**17**	3.54	35.4	**41**	8.53	85.4	**65**	13.52	135.4	**89**	18.51	185.4
**18**	3.74	37.5	**42**	8.74	87.5	**66**	13.73	137.5	**90**	18.72	187.5
**19**	3.95	39.6	**43**	8.94	89.6	**67**	13.94	139.6	**91**	18.93	189.6
**20**	4.16	41.7	**44**	9.15	91.7	**68**	14.14	141.6	**92**	19.14	191.6
**21**	4.37	43.7	**45**	9.36	93.7	**69**	14.35	143.7	**93**	19.34	193.7
**22**	4.58	45.8	**46**	9.57	95.8	**70**	14.56	145.8	**94**	19.55	195.8
**23**	4.78	47.9	**47**	9.78	97.9	**71**	14.77	147.9	**95**	19.76	197.9
**24**	4.99	50.0	**48**	9.98	100.0	**72**	14.98	150.0	**96**	20.00	200.0

Analyzing the quantitation output in Excel

- Copy the txt file fluorescence reads into Excel. Note that the top left number is the temperature. Also once you copy the numbers into excel the first row will be adjusted 2 rows to the left, so correct this before going on.
- Use the excel template/protocol if you're not sure how to do the calculations, but essentially you:
- Calculate the average for the standards and minus the blank from the standards
- Make a scatterplot from the average standards (plotting the fluorescence versus the DNA concentration of the DNA standards)
- Add a trend line with the intercept going through 0 and display the equation on the chart
- Minus the blank from all of your fluorescence values

- Divide your values by the linear equation given in the scatterplot. This is your DNA concentration. If you used 10 µl of the DNA template then divide this value by 10 to get ng/µl. If you only used 1 µl diluted in 9 µl of water you already have ng/µl!
- For single sample libraries multiply by the total volume in your sample.

Step 4: Pooling samples (not necessary if doing single libraries)

Although pooling is currently controversial and computationally more difficult to handle it may be your only option when dealing with population questions. Also with pooling you can't currently pull out the individual's information once they're pooled, but we are looking into adding an additional index before pooling to allow us to do this.

You'll find each extraction will have wildly variable quantities of DNA, which will affect the total amount of DNA you can pool, which will in turn affect which kit you'll use, see below.

How to Pool

First you need to know:

- which elution you want to use for each sample (look at your gels to pick)
- how many samples you want to pool
- the ng/µl for each sample
- the total volume for each sample
- whether you want a backup-if you do, make sure you have enough for 2 pools

For example: if you want to pool 30 samples, divide the target total ng of DNA you want (1300ng) by the number of (30).
1300ng/30samples=43.3ng/sample
So you will need 43.3ng from each sample to give you an equally pooled library totalling 1300ng.
Once you know this you use the microplate results (ng/µl) to tell you how many µl you need to add from each sample.

There are 2 ways to pool your samples, you can either

- Calculate the µl needed per sample individually i.e. sample 1a has 4.7ng/µl so you need to add 9.21µl of this sample (43.3ng/4.7=9.21µl) and sample 1b has 18.2ng/µl so you need to add 2.38µl of this sample (43.3ng/18.2=2.38µl). I do this in excel and have each part of the equation in different columns so I can change things easily.
- Dilute all of you samples to the same concentration i.e. 4ng/µl and add the same volume from each sample to make your pools (i.e. 43.3ng/4 = 10.83µl from each sample). This is initially time consuming, but if things don't work out it's easy to go back and re-pool. It can also be a little less stressful as adding water to a sample to change its concentration is easier to correct than adding the wrong volume of a sample into the final pool. However, you are diluting to the lowest common denominator, so your end volume may be huge.

Deciding on which way to pool can depend on the variability in your sample concentrations, how many samples you need to pool and personal preference. You also have to bear in mind that you'll need to get this pool down

to 25-44µl for the digestion, so you'll have to spin down your samples in the Speed-Vac if you have a high volume pool, which can result in your DNA forming a pellet, so you'll have to be aware of this fact.

If your samples in a pool have vastly varying concentrations it can become difficult to find the pooling sweet spot. It's difficult to pipette <1µl so it's unlikely to be accurate, so avoid this if possible. On the other hand all extractions only have a limited volume and spinning large volumes takes time. So you'll need to play around with the total amount of DNA in the pool (I've gone down to 30ng), this means you need less DNA from each individual. Also try to avoid removing samples as this increases the total ng needed from the rest of the samples. If you quantified multiple elutions and your first choice of elutions doesn't have enough DNA move on to your 2nd choice.

Step 5: Choose your library preparation kit

You now know how much DNA you have for each library (pooled or single), including a back-up, you now need to pick a kit.

Library prep kits and their required DNA input (after digestion and bead clean)

Illumina TruSeq PCR-free- 1ug
(I aim for 1.3ug before the digest and will process samples down to 700ng with this kit)

- This kit is good if you want to avoid PCR-ing your libraries

Illumina TruSeq Nano- 100-200ng

- This kit is good if you have less DNA

Kapa Hyper Prep Kit-1ng-1ug

- This kit is cheaper than Illumina, quicker to prep, can deal with a variety of input DNA, has less bead clean steps where you lose DNA, tailors the number of PCR cycles depending on how much DNA you put in each library. But you need to have your own adapters and beads (AMPure for cleaning and SPRI for size selecting)

Buying adapters separately:

This can be a cheaper way of making libraries, especially if you plan on using the ezRAD protocol a lot. However, there are some potential pitfalls to not using recommended adapters. Making your own adapters can take more time and be costly if they don't work first time, but can potentially save you a lot of money in the long-run. If you have Y's on the end of these adapters they can make size selection with e.g. the pippin prep machine inaccurate. Also outright don't buy **Nexterra adapters** to use with the Kapa or Illumina kits, they require PCR reactions to attach the adapters, whereas these kits use ligation so they won't work.

TO SAVE MONEY WE USE ½ REACTIONS FOR ALL KITS

Step 6: Concentrating your DNA

Option 1: SpeedVac (if eluted in water)

You have to reduce the volume of liquid to between 25-44 μl to proceed to the next step. Depending on the initial volume of liquid it can take a few hours to do this. Until you're comfortable using it only do 30 – 45 min at a time and keep an eye on how fast it evaporates. Avoid completely drying the sample! Do not use heat.

Note: if you're evaporating your volume down a lot the DNA may form a pellet. To resuspend it just flick the tube several times and possibly leave it overnight in the fridge for the DNA to resuspend fully.

Note: you can get the volume down to anything between 25-44 μl because the digestion in the next step includes adding 19 μl water, so if your sample is in water you can just aim for 44 μl instead.

Option 2: Bead clean (if eluted in water or buffer solution)

You probably only want to do this if you have a small volume or really don't like the other 2 options below. The magnetic plates used in the rest of the protocol only fit 250 μl tubes (strip tube size) so this is around your maximum total volume (one of the bead cleans in the protocol goes up to 280 μl but this is dangerously near the top). The total volume depends on the ratio of DNA to beads, for a clean before digestion, I would do a 1:0.6 ratio of DNA:beads. See below for instructions on this protocol. Note also that the AMPure XP beads are expensive (≈ $1000 a bottle), so this may not be an option. Aline PCRClean DX beads are a cheaper option for both SPRI and AMPure XP beads.

Option 3: Spin columns (if eluted in a buffer solution)

Spin columns are a fast way to concentrate you're samples, but there is a fairly high loss so you need a lot more DNA to start with (at least double). Example products include:

Amicon: http://www.millipore.com/catalogue/module/c84684

Microcon: http://www.millipore.com/catalogue/module/c113861

Option 4: Precipitation protocol (if eluted in a buffer solution) - See Appendix 2

Step 7: Bead clean-up

Once you have your extractions ready you can choose to do a bead clean on them before doing the digest. It removes contaminants, which may result in the digestion working better. These clean-up beads are designed to remove smaller fragments of DNA from your sample as well as remove any other reagents from the previous step. The ratio of sample to beads will determine what size fragments are removed. It does not matter on the concentration of DNA in your samples, but rather the volume.

Which beads to use?

There are several brands of magnetic beads on the market. The Illumina kits use the Beckman Coulter SPRIselect beads as their sample purification beads (SPBs). SPRI beads can be used for cleaning and size selection. They are more expensive than the AMPure XP beads, as they are calibrated and therefore better for size-selecting. Aline Biosciences also offers a product called PCRClean DX beads, which are a good and cheaper alternative the AMPure XP beads and even the SPRI beads, but I would check each batch for its calibration just to make sure. There are many more beads out there, but I haven't used them and so can't offer an opinion on them.

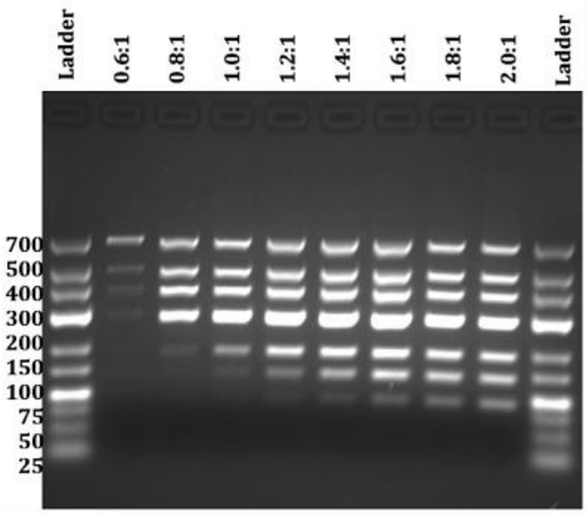

Figure 3. AMPure bead results at different DNA: bead ratios. (Taken from this website)

How the beads work

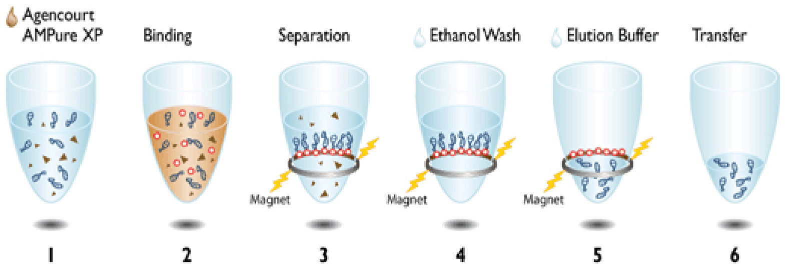

Figure 4. How the beads work: 1. DNA template without beads 2. Binding of DNA to magnetic beads 3. Separation of DNA bound to magnetic beads from contaminants 4. Washing of DNA with Ethanol 5. Elution of DNA from the magnetic particles 6. Transfer away from the beads into a new tube. Image taken from the Beckman Coulter website.

Bead cleaning method

Reagents/Equipment required:

- AMPure beads (stored in the fridge) - Let the bottle warm up to room temperature for at least 30 minutes!
- Resuspension buffer/low TE buffer/HPLC grade water
- Magnetic plate (see Appendix 1)
- 80% Ethanol (freshly made)
- Multiwell pipettes (optional)
- Reagent reservoir/trough (for use with the multiwall pipettes)
- Waste container
- Strip tubes
- Thermocycler
- Timer

Method:

To avoid contamination aliquot a subset of the beads into a separate tube before starting.

Shake the AMPure bead bottle until beads appear well mixed. This is very important. It is suggested to vortex them for 1 minute before starting.

The volumes will vary depending on what step in the protocol you are, see below (Table 3) for a quick reference table.

1. Shake the beads. This needs to be done every 3-4 samples to ensure the beads stay well mixed!
2. Pipette the beads into the sample (see each step for specific volumes) and mix gently by pipetting up and down (use wide bore pipettes if possible).
3. **Incubate** at room temperature for **5 minutes**.
4. Place samples onto the **magnetic stand for 5 minutes** or until the liquid appears clear.
5. With the samples remaining on the magnetic stand, gently **remove the supernatant**, leaving 5 µl in the tube (this reduces the likelihood of sucking up any beads). If you want to check whether you have accidentally sucked up any beads hold the pipette over a white surface, up to the light or put the supernatant in a separate tube and put it back on the magnetic stand.
6. With the samples remaining on the magnetic stand, add **200 µl of fresh 80% ethanol** without disturbing the beads. You can use a multiwall pipette and a reagent reservoir/trough for this if you want.
7. **Incubate at room temperature for 30 seconds**, then remove and discard the supernatant. You can also use the multiwell pipette for this as the DNA sticks to the side of the tube once the ethanol is added.
8. **Repeat** step 6 for a total of 2 washes.
9. Remove all the remaining ethanol from the bottom or sides, use a 20µl pipette if necessary (multiwell optional).
10. With the samples remaining on the magnetic stand, **let the samples air dry at room temperature for 5 minutes**. It is important that the beads don't dry out as it effects the DNA release from the beads.

11. Remove the samples from the magnetic stand and **resuspend** the dried pellet in the desired volume of resuspension buffer. If you use less than 10 µl you may not get all DNA back into solution. Gently pipette the entire volume up and down 10 times to mix the beads back into solution (use wide bore pipettes if possible).
12. **Incubate the samples at room temperature for 5 minutes** (no more than 10 mins).
13. Place the samples back onto the **magnetic stand for 5 minutes** or until the liquid is clear.
14. **Transfer the supernatant** to a new labelled collection tube.

Optional steps:

- If you want **to quantify** your sample after a bead-clean step just elute your sample in an additional 3 µl of resuspension buffer.
- After the bead clean you can also add 5 µl to the leftover beads and **keep them for later** just in case, as sometimes not all of the DNA is eluted from the beads the first time.

Note: whatever final volume you always want leave 2.5-5.0 µl of buffer on the beads so you can cleanly get your DNA back without pipetting up any beads.

Step 8: Digest

This digestion step with this frequent cutter restriction enzyme is the distinguishing feature in ezRAD protocol and is the equivalent of shearing the DNA or using other less frequent cutters. This digest reaction uses the enzymes DpnII, which cleaves sequences at GATC cut sites, for more information see Appendix 1. The protocol assumes the digestion of one unit per reaction, which is defined as the amount of enzyme required to **digest 1 µg of λ DNA** in 1 hour at 37°C in a total reaction volume of 50 µl.

DpnII comes in different concentrations, you may need to dilute it. To dilute it use 1x CutSmart buffer (1 CutSmart buffer: 9 water).

Note: We normally digest 1.2-1.3 µg per library because the DNA quantification is not 100% reliable and the potential for loss at each step is to be expected so going in with a little more gives a higher chance of the library working.

Note: If the digestion isn't working too well or you're concerned it might not work well then do a bead clean before doing the digest.

Note: You can run your back-up in the same digestion backup, elute like a full reaction and then just take ½.

Reagents required:

Table 3. Master mix for the DpnII restriction enzyme digestion per reaction (50 µl) for approximately 1ug of DNA. Add the reagents to the DNA in this order.

Digest Reagents	Volume µl (per sample)
DNA	**25**
Buffer (3.1)	**5**
HPLC grade H_2O	**19**
DpnII	**1**
**Total volume**	**50**

Note: If your sample is in water, rather than spinning it down to 25µl, you can save time by reducing the amount of water to 44µl instead. Then all you need to do is add 5µl of the 3.1 Buffer and 1µl of the DpnII restriction enzyme.

Digest Method:

1) Add the reagents to give a **total of 50 µl per reaction.**
2) Mix gently by pipetting up and down or flick and spin down.
3) Run the **DpnII** program in your thermocycler (if you don't have one make one) incubating the samples for:

   3 hours at 37°C
   20mins at 65°C
   Hold at 15°C

Step 9: Post-digest clean-up and checks

Post-digest Bead Clean

- Perform a 1:1.8 DNA:Beads clean (see step 7)
- Add **90 µl Ampure XP beads** to **50 µl DNA**
- Follow the standard bead clean procedure
- At the end of the bead clean protocol **re-suspend:**

For Illumina kit 1/2 reaction

- **38 µl** and **transfer 33 µl** to a new tube-
- Leaving **30 µl for the Illumina kit** after you run a gel

For Kapa kit 1/2 reaction

- **33 µl** and **transfer 28 µl** to a new tube-
- Leaving **25 µl for the Kapa kit** after you run a gel

Run a gel on your digested samples

Run a gel using 3µl, it should look like a smear now that the DNA has been digested. Compare it to your extractions (if possible on the same gel) to see if it's worked. If you still have a high band it hasn't worked, if your extraction was already a smear it will have shifted down. Check the restriction enzyme hasn't expired. If you didn't bead clean your sample before the digest then maybe just try running it again. It's unlikely that running the digest for longer will help. Maybe try other enzymes, we were previously using both MboI and Sau3AI and these worked for almost all samples. When working with sponges we found MboI and Sau3AI worked better than DpnII.

Quantify DNA (optional)

Use the AccuClear method to quantify your samples again to make sure you didn't lose too much DNA during the bead clean (refer to step 2). Aliquot 1 µl of your sample into 9 µl of H_2O for DNA at this step quantification.

Important! It's at this point that you want to have a **standardized labelling system** that you will call each library throughout the process. You may have decided this before your extraction, but often after multiple extractions this can get confusing. The labels/tube IDs will be the ones you now label every corresponding tube and will give the sequencing lab so that when you get your data back this is how they will be known when you're analyzing them. So make them distinct, but not too long because you're going to be writing them out a lot, and not too simple that other people might use the same code and it gives you little information e.g. P1 (*Porites* 1). At most you want 4-5 letters/numbers. A good example is PlSc1 (*Porites lobata*, Sharks Cove 1), this label gives you a lot of information to the user just by looking at it and is easier when analyzing your data.

Step 10: Make libraries!

Well done for getting this far! Getting your DNA to this point it actually the hardest aspect of library preparation.

If this is the first time you're making libraries, only make 1 library the first time (and leave a back-up). The reagents are not cheap and 9 out of 10 times it doesn't work 1st time.

Illumina library prep (pg. 1/6)
(For the experienced user version see Appendix 4)

Illumina TruSeq DNA library protocol

- As described in step 5 there are a couple of Illumina kits to choose from, a TruSeq PCR-free kit (requiring 1ug input DNA) and a TruSeq Nano kit (requiring 1-200ng input DNA). The steps for both are the same but with the addition of a DNA enrichment (PCR) step for the Nano kit, which is not necessary with the PCR-free kit.

Notes before starting the library prep:

- There are several steps that require the thermocycler, to save time program them in before you run through the protocol the first time (use the program names given by Illumina).
- All the reagents are in the freezer except the magnetic beads and possibly resuspension buffer.
- Get out everything needed for bead cleaning. Have the beads at room temperature. Make fresh 80% Ethanol; get out the magnetic plate and disposable troughs etc.
- Defrost all reagents on the bench slowly, keep them on ice when not in use and return to the freezer directly after using them. Mix them thoroughly by flicking them and spin down briefly before using them.
- Keep to your succinct labelling system! Label all tubes with the step you're at (or about to do). I use labels like "To ERP" which means I know this tube went into the thermocycler to be end-repaired.
- ALL VOLUMES BELOW ARE ½ REAGENTS

Illumina library prep (pg. 2/6)

1) End-repair-Illumina

Add all the end-repair components together into each sample. Mix thoroughly by flicking, briefly spin-down.

End-repair components	Volume µl
DNA	**30**
End Repair Mix 2	**20**
**Total volume**	**50**

Mix gently by flicking or pipetting up and down and briefly spin down.

Run the **ERP** program in you thermocycler:

Preheat the thermocycler **lid to 100°C**
Incubate the samples for:
30mins at 30°C
Hold at 4°C

Immediately remove the samples from the thermocycler and proceed to next step (size selection)

2) Size selection-Illumina

1) Vortex SPBs- make sure they are mixed well
2) In a 2, 5 or 15ml tube (depending on the number of libraries) make a master mix of diluted beads by adding **per library**:

   350bp insert:

   - 54.6 µl of SPBs (SPRI beads)
   - 37.4 µl of HPLC grade water
   - E.g. for 12 libraries mix 655 µl SPBs and 449 µl water

3) Mix and spin down the master mix.
4) Slowly aspirate and dispense **80 µl of the diluted beads** to each well **with** your **50 µl of end-repaired sample** (mix the beads every 3 samples). This removes the large DNA fragments.
5) Gently pipette the mixture up and down until well mixed (use wide-bore pipettes if available).
6) **Incubate** at room temperature for **5 minutes**.
7) Place samples onto the **magnetic stand for 5 minutes** or until the liquid appears clear.

Illumina library prep (pg. 3/6)

8) Label a new strip tube
9) The supernatant is the DNA you want, so **transfer 125 µl to the new strip tube**. Be careful not to disturb the beads
10) Keep the beads just in case.
11) Do a bead clean (see step 7), adding **15 µl of undiluted beads** to each sample containing the **125 µl of supernatant**.
12) At the end of the bead clean protocol **re-suspend in 12 µl** and **transfer 9 µl** to a new tube.

Safe Stopping point. If you don't proceed to the next step immediately you can store the samples in the freezer (-15 to -25) for up to 7 days.

3) Adenylate 3' Ends (A-tailing) -Illumina

Add all the A-tailing components together into each sample. Mix thoroughly by flicking, briefly spin-down.

A-tailing components	Volume µl
DNA	**9**
A-Tailing Mix	**6**
**Total volume**	**15**

Mix gently by flicking or pipetting up and down and briefly spin down.

Run the **ATAIL70** program in your thermocycler:

Preheat the thermo cycler **lid to 100°C**
Incubate the samples for:
30 mins at 37°C
5 mins at 70°C
5mins at 4°C
Hold at 4°C

Immediately remove the samples from the thermocycler and proceed to next step (ligate Adapters).

Illumina library prep (pg. 4/6)

4) Adapter ligation-Illumina

Before you start this step work out which adapters you're going to use and make a note of them in your lab book. You must know this to get your data. Don't duplicate adapters on the same sequencing run!

Add all the adapter ligation components together into each sample. Mix thoroughly by flicking, briefly spin-down.

Adapter ligation components	Volume µl
DNA	**15**
Resuspension Buffer	**1.4**
Ligation Mix 2	**1.3**
DNA Adapter Index	**1.3**
**Total volume**	**19**

Mix gently by flicking or pipetting up and down and briefly spin down.

Run the **LIG** program in your thermocycler:

Preheat the thermo cycler **lid to 100°C**
Incubate the samples for:
10 mins at 30°C
Hold at 4°C

- Add **2.5 µl of Stop Ligation Buffer** and mix gently by pipetting up and down, now giving you **21.5 µl DNA.**

Immediately remove the samples from the thermocycler and proceed to next step (Post ligation clean-up).

Illumina library prep (pg. 5/6)

5) Post ligation clean-up-Illumina

Perform a 1:1 DNA:Beads clean

Add **21.5 µl AMPure XP beads**

To **21.5 µl DNA**

Follow the standard bead clean procedure (see step 7)

At the end of the clean-up for:

- **TruSeq PCR-free kit** (1µg input DNA)

Elute in **30 µl Resuspension Buffer,** transfer **25 µl** into a 1.5ml tube with your name, individual sample ID code, and index adapter number written on the side and just the ID on the lid. Take**5 µl** to run a gel and NanoDrop to submit with finished libraries.
Go to step 11 (Validate your library)

- **TruSeq Nano** (200 ng input DNA)

Elute in **15 µl Resuspension Buffer** and transfer **12.5 µl** into a new strip tube ready for PCR**. Proceed to the next step (Enrich DNA fragments)**

Safe Stopping point. If you don't proceed to the next step immediately you can store the samples in the freezer (-15 to -25) for up to 7 days.

Illumina library prep (pg. 6/6)

6) Enrich DNA fragments- Nano kit only-Illumina

Add all PCR components together into each sample. Mix thoroughly by flicking, briefly spin-down.

PCR components	Volume µl
DNA	**12.5**
PCR Primer Cocktail	**2.5**
Enhanced PCR Master Mix	**10**
**Total volume**	**25**

Mix gently by flicking or pipetting up and down and briefly spin down.

Run the **PCRNano** program in your thermocycler:

Preheat the thermo cycler **lid to 100°C**

Incubate the samples at:

3 mins at 95°C
Then for **8 cycles**:
20 secs at 98°C
15 secs at 60°C
30 secs at 72°C
Then:
5 mins at 72°C
Hold at 4°C

7) Post-amplification clean-up-Illumina

Perform a 1:1 DNA:Beads clean

Add **25 µl AMPure XP beads**

To **25 µl DNA**

Follow the standard bead clean procedure (see step 7)
At the end of the clean-up elute the sample in **30 µl Resuspension Buffer** and **transfer 25 µl** of the clear supernatant to a new tube.
Transfer 20 µl of the clear supernatant to a new tube 1.5ml tube with your name, individual sample ID code, and index adapter number written on the side and just the ID on the lid.
Transfer 5 µl to a new strip tube-use this to validate your libraries

Go to Step 11 (Validate your library)

Kapa library prep (pg. 1/6)

(For experienced user version see Appendix 5)

KAPA Hyper Prep DNA library protocol

Notes before starting the library prep:

- There are several steps that require the thermocycler, to save time program them in before you run through the protocol the first time (use the program names given in this protocol).
- All the reagents are in the freezer except the magnetic beads and possibly resuspension buffer
- Get out everything needed for bead cleaning. Have the beads at room temperature. Make fresh 80% Ethanol; get out the magnetic plate and disposable troughs etc.
- Defrost all reagents on the bench slowly, keep them on ice when not in use and return to the freezer directly after using them. Mix them thoroughly by flicking them and spin down briefly before using them.
- Keep to your succinct labelling system! Label all tubes with the step you're at (or about to do). I use labels like 'To ERP' which means I know this tube went into the thermocycler to be end-repaired.
- ALL VOLUMES BELOW ARE ½ REAGENTS

1) End-repair and A-tailing-Kapa

Add all the end-repair and a-tailing components together into each sample. Mix thoroughly by flicking, briefly spin-down.

End-repair and A-tailing components	Volume µl
DNA	**25**
End-Repair & A-Tailing Buffer	**3.5**
End-Repair & A-Tailing Enzyme Mix	**1.5**
**Total volume**	**30**

Mix gently by flicking or pipetting up and down and briefly spin down.

Run the ERP&ATL program in your thermocycler:

Incubate the samples for:
30 mins at 20°C
30 mins at 65°C
Hold at 4°C

Proceed immediately to the next step!

Kapa library prep (pg. 2/6)

2) Adapter ligation-Kapa

Before you start this step work out which adapters you're going to use and make a note of them in your lab book. You must know this to get your data.

Depending on how much DNA you input to the Kapa library prep, it may be necessary to dilute the adapter stock, see table below to check. If using the Illumina adapters they're at 15 μM.

Input DNA	Adapter stock concentration μM
1000μg- 50ng	**15 μM**
25 ng	**7.5 μM**
10 ng	**3 μM**
5 ng	**1.5 μM**
2.5 ng	**750 nM**
1 ng	**300 nM**

Add all the adapter ligation components together into each sample. Mix thoroughly by flicking, briefly spin-down.

Adapter ligation components	Volume μl
DNA	**30**
HPLC-grade water	**2.5**
Ligation Buffer	**15**
DNA ligase	**5**
Adapter	**2.5**
**Total volume**	**55**

Mix gently by flicking or pipetting up and down and briefly spin down.

Run the LIG-ADAP program in your thermocycler:

Incubate the samples for:
15 mins at 20°C
Hold at 4°C

Proceed immediately to the next step!

Kapa library prep (pg. 3/6)

3) Post ligation clean-up-Kapa

Perform a 1:0.8 DNA:Beads clean

Add **44 µl AMPure XP beads**

To **55 µl DNA**

Follow the standard bead clean procedure (see step 7)

At the end of the clean-up elute the sample in **28 µl Resuspension Buffer** and **transfer 25 µl** of the clear supernatant to a new tube.

Safe Stopping point. If you don't proceed to the next step immediately you can store the samples in the freezer (-15 to -25) for up to 7 days.

4) Size selection (for 350-700bp insert) -Kapa

1) Add **12.5 µl SPRI beads**

   To **25 µl DNA** (removes large fragments)

2) Gently pipette the mixture up and down until well mixed (use wide-bore pipettes if available).
3) **Incubate** at room temperature for **5 minutes**.
4) Place samples onto the **magnetic stand for 5 minutes** or until the liquid appears clear.
5) Label a new strip tube
6) The supernatant is the DNA you want, so **transfer 35 µl to the new strip tube**. Be careful not to disturb the beads
7) Keep the beads just in case.
8) Add **5 µl SPRI beads** (Not AMPure XP beads!)

   To **35 µl DNA supernatant** (removes small fragments)

9) Do a regular bead clean (see step 7) i.e. add beads, leave for 5 mins, put on plate, leave for 5 mins, ethanol wash etc.). Make sure the beads don't dry out! The small volume of beads in this bead clean makes them more susceptible to drying out and cracking so keep an eye on them.

10) At the end of the bead clean protocol **re-suspend in 15 µl** and **transfer 10 µl** to a new tube. Or transfer 11 µl if you wish to nanodrop them before running the PCR (see next step).

Safe Stopping point. If you don't proceed to the next step immediately you can store the samples in the freezer (-15 to -25) for up to 7 days.

Kapa library prep (pg. 4/6)

5) Library amplification-Kapa

The KAPA kit differs from the Illumina protocol in that you decide how many PCR cycles you want to run your samples for, which is dependent on the amount of DNA you have in each sample. The options are:
Option 1: PCR cycles based on input DNA
Determine how many PCR cycles you need based on how much DNA you had before starting the library preparation. This method works almost all of the time.

Input DNA	# cycles required to generate 1 µM
1µg	**2 - 3**
500 ng	**3 – 4**
250 ng	**4 – 6**
100 ng	**6 – 7**
50 ng	**7 – 8**
25 ng	**8 – 10**
10 ng	**11 – 14**
5 ng	**13 – 16**
2.5 ng	**15 – 17**
1 ng	**17 - 19**

Option 2: PCR cycles based on quantifications after adapter ligation and size selection
If you want to reduce the number of PCR cycles or are having difficulties with a sample then quantify your libraries with the NanoDrop just before the PCR and use this table instead:

Adapter ligated DNA	# cycles required to generate 1 µM
**500 ng**	**1 – 2**
**100 ng**	**3 – 4**
**50 ng**	**5 – 6**
**10 ng**	**7 – 8**
**5 ng**	**8 – 9**
**1 ng**	**11 – 12**
**500 pg.**	**12 – 13**

Kapa library prep (pg. 5/6)

What to do with multiple samples with different numbers of PCR cycles:

You may notice that some samples need a similar number of cycles, in which case I'd try and work out a compromise and put them in the thermocycler for the same number of cycles (I'd probably go with more than less cycles). Others are wildly different and may result in you splitting up strip tubes and running multiple thermocyclers. If you have to split up samples keep the samples in the same order and just remove the samples that need more/less cycles into new tubes and return them to the original strip tube for the bead clean.

Note: I've also noticed that if you have low quality DNA going in (a low band at the bottom) even after the size selection some of the small fragments still remain making you think you have more DNA if you quantify at this step. So go for more PCR cycles if this is the case. We've not had a subsequent problem with these remaining small fragments in the quality control steps and sequencing, as there is a further bead clean after the PCR which helps to remove them.

Running the PCR

Keep everything on ice. Add all the library amplification components together into each sample. Mix reagents and the samples thoroughly by flicking, briefly spin-down. Make sure there are no bubbles, if there are flick them again till they're gone and spin-down again. Put them in the thermocyclers and push the caps down again.

Library amplification components	Volume µl
DNA	**10**
2X KAPA HiFi HotStart Ready Mix	**12.5**
10X Library Amplification Primer Mix	**2.5**
**Total volume**	**25**

Run the PCR program in your thermocycler:

45 secs at 98°C
Then for X **cycles**:
15 secs at 98°C
30 secs at 60°C
30 secs at 72°C
Then:
1 min at 72°C
Hold at 4°C

Kapa library prep (pg. 6/6)

6) Post-amplification clean-up-Kapa

Perform a 1:1 DNA:Beads clean

Add **25 µl AMPure XP beads**

To **25 µl DNA**

Follow the standard bead clean procedure (see step 7)

At the end of the clean-up elute the sample in **30 µl Resuspension Buffer** and **transfer 25 µl** of the clear supernatant to a new tube.

Transfer 20 µl of the clear supernatant to a new tube 1.5ml tube with your name, individual sample ID code, and index adapter number written on the side and just the ID on the lid.

Transfer 5 µl to a new strip tube-use this to validate your libraries

Add 10 µl of the resuspension buffer to your beads and mix them.

Store the beads as a backup in the freezer. The first elution from the beads does not necessarily give you all of your DNA.

Go to Step 11 (Validate your library)

Step 11: Validate your libraries

1) NanoDrop **1 µl** of your completed libraries. Aim for at least 10ng/µl. For 300bp reads you need the concentration at 4nM (a quick google search will give you an excel sheet which calculates ng/µl to nM for you, but the qPCR will give you the definitive concentration).
2) Run a gel using **3 µl** DNA and hyperladder 4 to check the results before freezing. ***Note***: The NanoDrop may not be reliable, so the gel might give you a better idea of how your library looks.
3) Fill out a MiSeq submission form providing the NanoDrop results and gel with the samples.
4) You can then hand your **completed libraries** (**20 µl)** to Amy Eggers at the Evolutionary Genetics Core Facility (EGCF) at the Hawaii Institute of Marine (HIMB), Coconut Island for QC/library validation (Bioanalyzer and qPCR) and sequencing on the MiSeq. It's also advisable that you email Amy (aeggers@hawaii.edu) letting her know that you're going to give her samples. You can also email Amy beforehand for a quote if you need one.

Note: Samples are not in the queue at the core lab until they are in their freezer and the paperwork has been submitted.

Final Note: All figures are taken from the websites/documents provided by the supplier.

See mistakes in the protocol? Make notes on your copy and let me know and I'll change it.

This a not for profit protocol and is designed to be used for educational purposes only. Any opinions given in this protocol are not those of the University of Hawaii or The Hawaii Institute of Marine Biology.

Appendix 1: Setting up a lab to do ezRAD

Below is a list of the lab equipment, kits and other reagents you'll need to have in order to do the ezRAD process in house, not including sample collection, gels, quality control checks (bioanalyzer and qPCR) or sequencing. I also assume here that you have a range of standard pipettes and tip sizes. I've added in hyperlinks to websites for the more unusual items.

Equipment:

- Multiwell pipettes (at least an 8 well 1-20µl and 20-200µl)
- 10ml or 20ml pipette (not essential, but makes quantitation easier for larger sample sizes)
- Vortex
- Centrifuge-for 1.5ml tubes and strip tubes
- Incubator/water bath (preferably one that shakes)
- 1.5ml tubes
- 250µl 8 well strip tubes
- 96-well plates and aluminium sealing films (if dealing with large numbers of samples)
- SpeedVac
- Black 96-well plates-solid bottom (not clear) from either Corning Costar plate (Cat# 3916) or Greiner Bio-One CELLSTAR plate (Cat. # 655079)
- Microplate reader: Molecular devises- SpectraMax M2 (or other models, the AccuClear assay is designed for use with fluorescence 96-well plate readers equipped with excitation and emission filters for detecting green fluorescence and is especially well-suited for use with instruments with blue LED excitation sources-see the AccuClear literature).
- Magnetic plate (for magnetic bead cleans)-DynaMag™-96 Side Magnet-we've tried 2 other styles of plates, this is definitely the best (even than the one recommended by Illumina).
- Reagent trough/reservoir-doesn't have to be from this site
- Freezable tube rack
- Timer

Kits:

- The Qiagen or Omega tissue and genomic DNA extraction kits.
- AccuClear Ultra high sensitivity dsDNA quantitation kit
- Illumina TruSeq DNA preparation kits
  - PCR-free kits (1000ng input DNA)
  - Nano kit (100ng input DNA)
- Kapa Hyper Prep Kit- If you want the option to amplify your libraries get the one with the Amplification Primer Mix (10X) (KK8500, KK8502 or KK8504). Don't get the Hyper Prep Plus kit, it includes fragmentation reagents that you don't need for this protocol.
- Both kits come in 2 options either:
  - (LT) low throughput (makes 24 libraries) or
  - (HT) High throughput (makes 96 dual indexed libraries)

Separate reagents (not included in kits):

- HPLC or PCR grade water
- RNase A- We currently use Amresco 0625_500MG, which comes in a powder and has to be made up by adding HPLC grade water to make a final concentration of 100 mg/ml. Another option is to buy it pre-made up like this one from Life Technologies.
- Agencourt AMPure XP magnetic beads-see here for the Beckman Colter protocol.
- SPRIselect beads- These are more expensive than the Agencourt AMPure AX beads, but are calibrated, so are better for size selecting.
- Aline PCRClean DX- magnetic beads-can be used as SPRI bead and AMPure XP alternative.
- DpnII restriction enzyme- If DpnII doesn't work with your samples there are other isoschizomers of this GATC cut site. We were initially using MboI and Sau3A1 in the same reaction, which worked well for us. We moved over to DpnII because, unlike MboI and Sau3A1, it is not sensitive to CpG methylation, which is found in higher eukaryotes. MboI and Sau3A1 are better at dealing with prokaryotic methylation, so if this is what you are targeting then use these 2 restriction enzymes. Methylation is an issue to consider when digesting DNA with restriction enzymes because they can be blocked or impaired when a particular base in the recognition site is methylated. To read about the different types of methylation see here. For differences between these 3 restriction endonucleases see here.

Appendix 2: Removed from this protocol, but still in old versions

Technologies move so fast that a number of steps could be removed from this 2.0 version of the ezRAD protocol, but in the case that you would still like to use them I will list what was removed here:

In ezRAD protocol version 1.5 but not in version 2.0 onwards:

- **AccuBlue high sensitivity kit procedure**- the main difference between the AccuBlue and AccuClear kits is that AccuClear is better suited to accurately measuring 0.2-100ng and the excitation and emission values are different. Note that there is also an AccuBlue broad range kit, which measures between 2 and 2000ng, which is good when you want to detect high concentrations of DNA, but we often end up on the lower end so use the AccuClear ultra high sensitivity kit.
- **Serial dilution of standards for quantitation**- there are instructions provided with the AccuClear kit if you order the one with 1 standard.
- **Information on previously used digestion enzymes (MboI and Sau3AI)**
- **Tips when using the ring magnetic plate**
- **Gel excision**
- **Flashgel method**
- **DNA concentration using the Precipitation protocol** (if eluted in a buffer solution) - This protocol has long been used to concentrate DNA, but personally I don't like how long it takes to do and often the outcome is disappointing. We found Isopropanol (isopropyl alcohol) worked better than ETOH because of the high total volumes, but ethanol (ethyl alcohol) is most commonly used alcohol for DNA precipitation. This protocol can easily be found on the internet.

Appendix 3: Illumina adapter sequences

Sorted by adapter number or adapter set

NOTE (taken from the Illumina TruSeq adapter preparation Guide)

- The index numbering is not contiguous. Index 17, 24, and 26 are skipped.
- The base in parentheses () indicates the base for the seventh cycle and is not considered as part of the index sequence. The index should be recorded in the sample sheet as only six bases. For indexes 13 and above, the seventh base (in parentheses) might not be A, and this will be seen in the seventh cycle of the index read.

Sorted by adapter number

Adapter	Sequence	Set
**AD001**	ATCACG(A)	B
**AD002**	CGATGT(A)	A
**AD003**	TTAGGC(A)	B
**AD004**	TGACCA(A)	A
**AD005**	ACAGTG(A)	A
**AD006**	GCCAAT(A)	A
**AD007**	CAGATC(A)	A
**AD008**	ACTTGA(A)	B
**AD009**	GATCAG(A)	B
**AD010**	TAGCTT(A)	B
**AD011**	GGCTAC(A)	B
**AD012**	CTTGTA(A)	A
**AD013**	AGTCAA(C)	A
**AD014**	AGTTCC(G)	A
**AD015**	ATGTCA(G)	A
**AD016**	CCGTCC(C)	A
**AD018**	GTCCGC(A)	A
**AD019**	GTGAAA(C)	A
**AD020**	GTGGCC(T)	B
**AD021**	GTTTCG(G)	B
**AD022**	CGTACG(T)	B
**AD023**	GAGTGG(A)	B
**AD025**	ACTGAT(A)	B
**AD027**	ATTCCT(T)	B

Sorted by adapter set (A or B)

Adapter	Sequence	Set
**AD002**	CGATGT(A)	A
**AD004**	TGACCA(A)	A
**AD005**	ACAGTG(A)	A
**AD006**	GCCAAT(A)	A
**AD007**	CAGATC(A)	A
**AD012**	CTTGTA(A)	A
**AD013**	AGTCAA(C)	A
**AD014**	AGTTCC(G)	A
**AD015**	ATGTCA(G)	A
**AD016**	CCGTCC(C)	A
**AD018**	GTCCGC(A)	A
**AD019**	GTGAAA(C)	A
**AD001**	ATCACG(A)	B
**AD003**	TTAGGC(A)	B
**AD008**	ACTTGA(A)	B
**AD009**	GATCAG(A)	B
**AD010**	TAGCTT(A)	B
**AD011**	GGCTAC(A)	B
**AD020**	GTGGCC(T)	B
**AD021**	GTTTCG(G)	B
**AD022**	CGTACG(T)	B
**AD023**	GAGTGG(A)	B
**AD025**	ACTGAT(A)	B
**AD027**	ATTCCT(T)	B

Appendix 4: Illumina experienced user version

1) End-repair

End-repair components	Volume µl
**DNA**	**30**
**End Repair Mix 2**	**20**
Total volume	= 50

Run the **ERP** program in you thermocycler:

30mins at 30°C
Hold at 4°C

2) Size Selection (350bp insert)

- Dilute SPRI bead master mix per sample:
- 54.6 µl of SPBs (SPRI beads)
- 37.4 µl of HPLC grade water

- Add **80 µl** of the **Diluted beads** with **50 µl of DNA.** Take supernatant (**125 µl**), put in new tube.
- Add **15 µl of Undiluted beads** to each sample containing **125 µl of supernatant**, finish like a normal bead clean

3) Adenylate 3' Ends (A-tailing)

A-tailing components	Volume µl
**DNA**	**9**
**A-Tailing Mix**	**6**
Total volume	= 15

Run the **ATAIL70** program in your thermocycler:

30 mins at 37°C
5 mins at 70°C
5mins at 4°C
Hold at 4°C

4) Adapter ligation (Note adapters in your lab book)

Adapter ligation components	Volume µl
**DNA**	**15**
**Resuspension Buffer**	**1.4**
**Ligation Mix 2**	**1.3**
**DNA Adapter Index**	**1.3**
Total volume	= 19

Run the **LIG** program in your thermocycler:

10 mins at 30°C
Hold at 4°C

- Add **2.5 µl** of **Stop Ligation Buffer** giving you **21.5 µl DN**

5) Post Ligation Clean-up

- Perform a 1:1 DNA:Beads clean (**21.5 µl AMPure XP beads : 21.5 µl DNA)**
- **TruSeq PCR-free**-Elute in **30 µl,** transfer **25 µl** and take **5 µl** to run gel and NanoDrop. Submit 20 µl for library validation. Done!
- **TruSeq Nano-** Elute in **15 µl** and transfer **12.5 µl** for PCR

6) Enrich DNA fragments- Nano kit only

PCR components	Volume µl
**DNA**	**12.5**
**PCR Primer Cocktail**	**2.5**
**Enhanced PCR Master Mix**	**10**
Total volume	= 25

Run the **PCRNano** program in your thermocycler:

3 mins at 95°C, x1
20 secs at 98°C, x8 cycles
15 secs at 60°C
30 secs at 72°C
5 mins at 72°C, x1
Hold at 4°C

7) Post-amplification Clean-up-Nano kit only

- Perform a 1:1 DNA:Beads clean (**25 µl AMPure XP beads : 25 µl DNA)**
- Elute in **30 µl Resuspension Buffer**, **transfer 25 µl**, and take **5 µl** to run gel and NanoDrop. Submit 20 µl for library validation. Done!

Appendix 5: KAPA experienced user version

1) End-repair and A-tailing

End-repair & A-tailing	Volume µl
**DNA**	**25**
**End-Rep. & A-Tail. Buffer**	**3.5**
**End-Rep. & A-Tail. Enz. Mix**	**1.5**
Total volume	= 30

Run the **ERP&ATL program** in your thermocycler:

30 mins at 20°C
30 mins at 65°C
Hold at 4°C

2) Adapter Ligation (Note adapters in your lab book)

Adapter ligation components	Volume µl
**DNA**	**30**
**HPLC-grade water**	**2.5**
**Ligation Buffer**	**15**
**DNA ligase**	**5**
**Adapter**	**2.5**
Total volume	= 55

Run the **LIG-ADAP** program in your thermocycler:

15 mins at 20°C
Hold at 4°C

3) Post Ligation Clean-up

- Perform a 1:0.8 DNA:Beads clean (**44 µl AMPure XP beads** : **55 µl DNA)**
- Elute the sample in **28 µl Resuspension Buffer** and **transfer 25 µl**

4) Size Selection (for 350-700bp insert)

- Add **12.5 µl SPRI beads** to **25 µl DNA**
- Take supernatant (**35 µl**)
- Add **5 µl SPRI beads** to **35 µl DNA**, finish like a normal bead clean
- Elute the sample in **15 µl Resuspension** Buffer and **transfer 10 µl**

5) Library Amplification

- Work out # PCR cycles from tables below:

Input DNA	# cycles required to generate 1 µM
1µg	2 - 3
500 ng	3 – 4
250 ng	4 – 6
100 ng	6 – 7
50 ng	7 – 8
25 ng	8 – 10
10 ng	11 – 14
5 ng	13 – 16
2.5 ng	15 – 17
1 ng	17 - 19

Adpt. ligated DNA	# cycles required to generate 1 µM
500 ng	1 – 2
100 ng	3 – 4
50 ng	5 – 6
10 ng	7 – 8
5 ng	8 – 9
1 ng	11 – 12
500 pg	12 – 13

Library amplification components	Volume µl
**DNA**	**10**
**2X KAPA HiFi HotStart Ready Mix**	12.5
**10X Library Amplification Primer Mix**	2.5
Total volume	= 25

Run the **PCR** program in your thermocycler:

45 secs at 98°C, x1
15 secs at 98°C, x ? Cycles
30 secs at 60°C
30 secs at 72°C
1 min at 72°C, x1
Hold at 4°C

6) Post-amplification Clean-up

- Perform a 1:1 DNA:Beads clean (**25 µl AMPure XP beads** : **25 µl DNA)**
- Elute in **30 µl Resuspension Buffer**, **transfer 25 µl**, and take **5 µl** to run gel and NanoDrop. Submit 20 µl for library validation. Done!
