# Supplementary material for: Genomic DNA extraction optimization and validation for genome sequencing using the marine gastropod Kellet’s whelk
Source: PeerJ. 2023 Dec 6;11:e16510. doi: 10.7717/peerj.16510 (PMC10710129; doi:10.7717/peerj.16510)
Supplement: Supplemental Information 7 [file peerj-11-16510-s007.zip › Nanopore MinION Ligation Sequencing Kit.pdf]

Overview of the protocol

Ligation Sequencing Kit V14 features

This kit is recommended for users who:

- Want to achieve median raw read accuracy of Q20+ (99%) and above.
- Want to optimise their sequencing experiment for output.
- Require control over read length.
- Would like to utilise upstream processes such as size selection, whole genome amplification, or enrichment for long reads.
- Want to achieve duplex basecalling. For more information, please see the Kit 14 sequencing and duplex basecalling info sheet.

Introduction to the Ligation Sequencing Kit V14 (SQK-LSK114) protocol

This protocol describes how to carry out sequencing of a DNA sample using the Ligation Sequencing Kit V14 (SQK-LSK114). It is recommended that a Lambda control experiment is completed first to become familiar with the technology.

Steps in the sequencing workflow:

Prepare for your experiment

You will need to:

- Extract your DNA, and check its length, quantity and purity. **The quality checks performed during the protocol are essential in ensuring experimental success.**
- Ensure you have your sequencing kit, the correct equipment and third-party reagents
- Download the software for acquiring and analysing your data
- Check your flow cell to ensure it has enough pores for a good sequencing run

Library preparation

You will need to:

- Repair the DNA, and prepare the DNA ends for adapter attachment
- Attach sequencing adapters supplied in the kit to the DNA ends
- Prime the flow cell, and load your DNA library into the flow cell

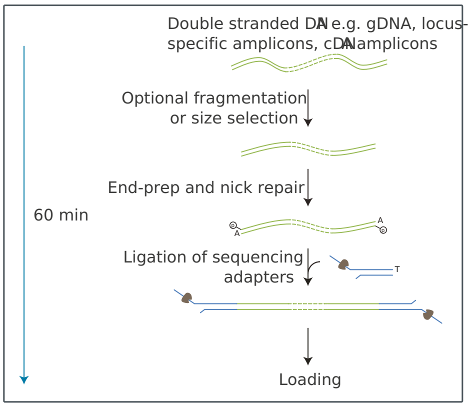

Sequencing and analysis

You will need to:

- Start a sequencing run using the MinKNOW software which will collect raw data from the device and convert it into basecalled reads

IMPORTANT

Compatibility of this protocol

This protocol should only be used in combination with:

- Ligation Sequencing Kit V14 (SQK-LSK114)
- Control Expansion (EXP-CTL001)
- R10.4.1 flow cells (FLO-MIN114)
- Flow Cell Wash Kit (EXP-WSH004)

Equipment and consumables

Materials

- 1 µg high molecular weight genomic DNA / 100-200 fmol amplicon DNA
- OR 100+ ng high molecular weight genomic DNA if performing DNA fragmentation
- Ligation Sequencing Kit V14 (SQK-LSK114)

Consumables

- NEBNext® Companion Module for Oxford Nanopore Technologies® Ligation Sequencing (NEB, E7180S or E7180L). Alternatively, you can use the NEBNext® products below:
- NEBNext FFPE Repair Mix (NEB, M6630)
- NEBNext Ultra II End repair/dA-tailing Module (NEB, cat # E7546)
- NEBNext Quick Ligation Module (NEB, cat # E6056)
- 1.5 ml Eppendorf DNA LoBind tubes
- 0.2 ml thin-walled PCR tubes
- Nuclease-free water (e.g. ThermoFisher, AM9937)
- Freshly prepared 80% ethanol in nuclease-free water
- Qubit™ Assay Tubes (Invitrogen, Q32856)

- Qubit dsDNA HS Assay Kit (Invitrogen, Q32851)
- (Optional) Bovine Serum Albumin (BSA) (50 mg/ml) (e.g Invitrogen™ UltraPure™ BSA 50 mg/ml, AM2616)

**Equipment**	• Hula mixer (gentle rotator mixer)<br>• Magnetic separator, suitable for 1.5 ml Eppendorf tubes<br>• Microfuge<br>• Vortex mixer<br>• Thermal cycler<br>• P1000 pipette and tips<br>• P200 pipette and tips<br>• P100 pipette and tips<br>• P20 pipette and tips<br>• P10 pipette and tips<br>• P2 pipette and tips<br>• Ice bucket with ice<br>• Timer<br>• Qubit fluorometer (or equivalent for QC check)
**Optional Equipment**	• Agilent Bioanalyzer (or equivalent)<br>• Eppendorf 5424 centrifuge (or equivalent)

For this protocol, you will need 1 µg (or 100–200 fmol) of DNA.

For high molecular weight gDNA (>48 kb), we recommend using 1 µg as input.

If using amplicon DNA, we recommend using an input of 100-200 fmol.

Please ensure you are using the recommended input for your sample for optimal DNA yield from your library preparation.

Input DNA

How to QC your input DNA

It is important that the input DNA meets the quantity and quality requirements. Using too little or too much DNA, or DNA of poor quality (e.g. highly fragmented or containing RNA or chemical contaminants) can affect your library preparation.

For instructions on how to perform quality control of your DNA sample, please read the Input DNA/RNA QC protocol.

Chemical contaminants

Depending on how the DNA is extracted from the raw sample, certain chemical contaminants may remain in the purified DNA, which can affect library preparation efficiency and sequencing quality. Read more about contaminants on the Contaminants page of the Community.

NEBNext® Companion Module for Oxford Nanopore Technologies® Ligation Sequencing

For customers new to nanopore sequencing, we recommend buying the NEBNext® Companion Module for Oxford Nanopore Technologies® Ligation Sequencing (catalogue number E7180S or E7180L), which contains all the NEB reagents needed for use with

the Ligation Sequencing Kit.

Please note, for our amplicon protocols, NEBNext FFPE DNA Repair Mix and NEBNext FFPE DNA Repair Buffer are not required.

Third-party reagents

We have validated and recommend the use of all the third-party reagents used in this protocol. Alternatives have not been tested by Oxford Nanopore Technologies.

For all third-party reagents, we recommend following the manufacturer's instructions to prepare the reagents for use.

> **IMPORTANT**
>
> **We strongly recommend using the Ligation Buffer (LNB) supplied in the Ligation Sequencing Kit V14 rather than the third-party ligase buffer supplied in the NEBNext Quick Ligation Module to ensure high ligation efficiency of the Ligation Adapter (LA).**

> **IMPORTANT**
>
> **Ligation Adapter (LA) used in this kit and protocol is not interchangeable with other sequencing adapters.**

Ligation Sequencing Kit V14 (SQK-LSK114) contents

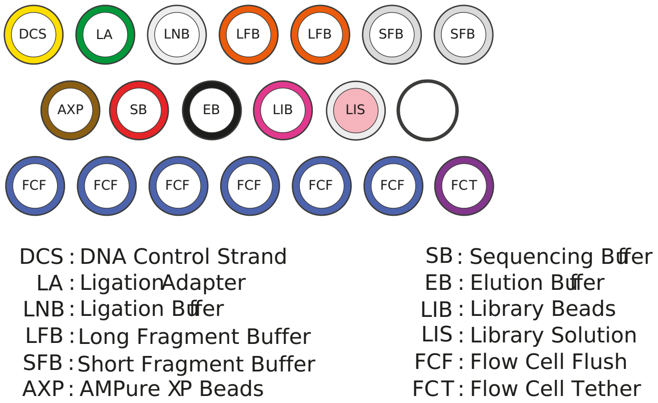

Name	Acronym	Cap colour	No. of vials	Fill volume per vial (µl)
DNA Control Strand	DCS	Yellow	1	35
Ligation Adapter	LA	Green	2	40
AMPure XP Beads	AXP	Amber	1	1,200
Ligation Buffer	LNB	White	1	200
Long Fragment Buffer	LFB	Orange	2	1,800
Short Fragment Buffer	SFB	Clear	2	1,800
Sequencing Buffer	SB	Red	1	700
Elution Buffer	EB	Black	1	1,200
Library Beads	LIB	Pink	1	600

Name	Acronym	Cap colour	No. of vials	Fill volume per vial (µl)
Library Solution	LIS	White cap, pink sticker on label	1	600
Flow Cell Flush	FCF	Blue	6	1,170
Flow Cell Tether	FCT	Purple	1	200

Note: This product contains AMPure XP Reagent manufactured by Beckman Coulter, Inc. and can be stored at -20°C with the kit without detriment to reagent stability.

Computer requirements and software

MinION Mk1C IT requirements

The MinION Mk1C contains fully-integrated compute and screen, removing the need for any accessories to generate and analyse nanopore data. Read more in the MinION Mk1C IT requirements document.

MinION Mk1B IT requirements

Sequencing on a MinION Mk1B requires a high-spec computer or laptop to keep up with the rate of data acquisition. Read more in the MinION IT Requirements document.

Software for nanopore sequencing

MinKNOW

The MinKNOW software controls the nanopore sequencing device, collects sequencing data and basecalls in real time. You will be using MinKNOW for every sequencing experiment to sequence and demultiplex if samples are barcoded. Please note that live demultiplexing in MinKNOW is not enabled for the Amplicon Barcoding Kit 24. Post-run demultiplexing using the standalone Guppy software is required.

MinKNOW use

For instructions on how to run the MinKNOW software, please refer to the relevant section in the MinKNOW protocol.

Guppy

The Guppy command-line software can be used for basecalling and demultiplexing reads by barcode instead of MinKNOW. You can use it if you would like to re-analyse old data, or integrate basecalling into your analysis pipeline.

Guppy installation and use

If you would like to use the Guppy software, please refer to the Guppy protocol.

EPI2ME (optional)

The EPI2ME cloud-based platform performs further analysis of basecalled data, for example alignment to the Lambda genome, barcoding, or taxonomic classification. You will use the EPI2ME platform *only* if you would like further analysis of your data post-basecalling.

EPI2ME installation and use

For instructions on how to create an EPI2ME account and install the EPI2ME Desktop Agent, please refer to the EPI2ME Platform protocol.

Check your flow cell

We highly recommend that you check the number of pores in your flow cell prior to starting a sequencing experiment. This should be done within three months of purchasing for MinION/GridION/PromethION flow cells, or within four weeks of purchasing for Flongle flow cells. Oxford Nanopore Technologies will replace any flow cell with fewer than the number of pores in the table below, when the result is reported within two days of performing the flow cell check, and when the storage recommendations have been followed. To do the flow cell check, please follow the instructions in the Flow Cell Check document.

Flow cell	Minimum number of active pores covered by warranty
Flongle Flow Cell	50
MinION/GridION Flow Cell	800
PromethION Flow Cell	5000

DNA repair and end-prep

~35 minutes

Materials

- 1 µg high molecular weight genomic DNA
- DNA Control Sample (DCS)
- AMPure XP Beads (AXP)

Consumables

- 0.2 ml thin-walled PCR tubes
- 1.5 ml Eppendorf DNA LoBind tubes
- Nuclease-free water (e.g. ThermoFisher, cat # AM9937)
- NEBNext FFPE DNA Repair Mix (NEB, cat # M6630)
- NEBNext Ultra II End Repair / dA-tailing Module (NEB, cat # E7546)
- Freshly prepared 80% ethanol in nuclease-free water
- Qubit™ Assay Tubes (Invitrogen, Q32856)
- Qubit dsDNA HS Assay Kit (Invitrogen, Q32851)

Equipment

- P1000 pipette and tips
- P100 pipette and tips
- P10 pipette and tips
- Thermal cycler at 20°C and 65°C
- Microfuge
- Hula mixer (gentle rotator mixer)
- Magnetic rack
- Ice bucket with ice

Optional Equipment

- Qubit fluorometer (or equivalent for QC check)

1 **Thaw DNA Control Sample (DCS) at room temperature, spin down, mix by pipetting, and place on ice.**

> TIP
>
> **We recommend using the DNA Control Sample (DCS) in your library prep for troubleshooting purposes. However, you can omit this step and make up the extra 1 µl with your sample DNA.**

2 **Prepare the NEBNext FFPE DNA Repair Mix and NEBNext Ultra II End Repair / dA-tailing Module reagents in accordance with manufacturer's instructions, and place on ice.**

For optimal performance, NEB recommend the following:

1. Thaw all reagents on ice.
2. Flick and/or invert the reagent tubes to ensure they are well mixed.
   Note: Do not vortex the FFPE DNA Repair Mix or Ultra II End Prep Enzyme Mix.
3. Always spin down tubes before opening for the first time each day.
4. The Ultra II End Prep Buffer and FFPE DNA Repair Buffer may have a little precipitate. Allow the mixture to come to room temperature and pipette the buffer up and down several times to break up the precipitate, followed by vortexing the tube for 30 seconds to solubilise any precipitate.
   Note: It is important the buffers are mixed well by vortexing.
5. The FFPE DNA Repair Buffer may have a yellow tinge and is fine to use if yellow.

3 **Prepare the DNA in nuclease-free water:**

1. Transfer 1 µg (or 100-200 fmol) input DNA into a 1.5 ml Eppendorf DNA LoBind tube.
2. Adjust the volume to 47 µl with nuclease-free water.
3. Mix thoroughly by pipetting up and down, or by flicking the tube.
4. Spin down briefly in a microfuge

4 **In a 0.2 ml thin-walled PCR tube, mix the following:**

Between each addition, pipette mix 10-20 times.

Reagent	Volume
DNA from the previous step	47 µl
DNA CS (optional)	1 µl
NEBNext FFPE DNA Repair Buffer	3.5 µl
NEBNext FFPE DNA Repair Mix	2 µl
Ultra II End-prep Reaction Buffer	3.5 µl
Ultra II End-prep Enzyme Mix	3 µl
**Total**	**60 µl**

5 **Ensure the reaction is thoroughly mixed by gently pipetting and spin down briefly.**

6 **Using a thermal cycler, incubate at 20°C for 5 minutes and 65°C for 5 minutes.**

7 **Resuspend the AMPure XP Beads (AXP) by vortexing.**

8 **Transfer the DNA sample to a clean 1.5 ml Eppendorf DNA LoBind tube.**

9 **Add 60 µl of resuspended the AMPure XP Beads (AXP) to the end-prep reaction and mix by flicking the tube.**

10 **Incubate on a Hula mixer (rotator mixer) for 5 minutes at room temperature.**

11 **Prepare 500 µl of fresh 80% ethanol in nuclease-free water.**

12 **Spin down the sample and pellet on a magnet until supernatant is clear and colourless. Keep the tube on the magnet, and pipette off the supernatant.**

13 **Keep the tube on the magnet and wash the beads with 200 µl of freshly prepared 80% ethanol without disturbing the pellet. Remove the ethanol using a pipette and discard.**

14 **Repeat the previous step.**

15 **Spin down and place the tube back on the magnet. Pipette off any residual ethanol. Allow to dry for ~30 seconds, but do not dry the pellet to the point of cracking.**

16 **Remove the tube from the magnetic rack and resuspend the pellet in 61 µl nuclease-free water. Incubate for 2 minutes at room temperature.**

17 **Pellet the beads on a magnet until the eluate is clear and colourless, for at least 1 minute.**

18 **Remove and retain 61 µl of eluate into a clean 1.5 ml Eppendorf DNA LoBind tube.**

Quantify 1 µl of eluted sample using a Qubit fluorometer.

> **END OF STEP**
>
> **Take forward the repaired and end-prepped DNA into the adapter ligation step. However, at this point it is also possible to store the sample at 4°C overnight.**

Adapter ligation and clean-up

~20 minutes

**Materials**	• Ligation Adapter (LA)<br>• Ligation Buffer (LNB) from the Ligation Sequencing Kit<br>• Long Fragment Buffer (LFB)<br>• Short Fragment Buffer (SFB)<br>• AMPure XP Beads (AXP)<br>• Elution Buffer (EB) from the Oxford Nanopore sequencing kit
**Consumables**	• NEBNext Quick Ligation Module (NEB, cat # E6056)<br>• 1.5 ml Eppendorf DNA LoBind tubes<br>• Qubit™ Assay Tubes (Invitrogen, Q32856)<br>• Qubit dsDNA HS Assay Kit (Invitrogen, Q32851)
**Equipment**	• Magnetic rack<br>• Microfuge<br>• Vortex mixer<br>• P1000 pipette and tips<br>• P100 pipette and tips<br>• P20 pipette and tips<br>• P10 pipette and tips<br>• Qubit fluorometer (or equivalent for QC check)

IMPORTANT

Although the recommended third-party ligase is supplied with its own buffer, the ligation efficiency of the Ligation Adapter (LA) is higher when using the Ligation Buffer (LNB) supplied in the Ligation Sequencing Kit.

1 **Spin down the Ligation Adapter (LA) and Quick T4 Ligase, and place on ice.**

2 **Thaw Ligation Buffer (LNB) at room temperature, spin down and mix by pipetting. Due to viscosity, vortexing this buffer is ineffective. Place on ice immediately after thawing and mixing.**

3 **Thaw the Elution Buffer (EB) at room temperature, mix by vortexing, spin down and place on ice.**

IMPORTANT

Depending on the wash buffer (LFB or SFB) used, the clean-up step after adapter ligation is designed to either enrich for DNA fragments of >3 kb, or purify all fragments equally.

- To enrich for DNA fragments of 3 kb or longer, use Long Fragment Buffer (LFB)
- To retain DNA fragments of all sizes, use Short Fragment Buffer (SFB)

4 **Thaw either Long Fragment Buffer (LFB) or Short Fragment Buffer (SFB) at room temperature, mix by vortexing, spin down and place on ice.**

5 **In a 1.5 ml Eppendorf DNA LoBind tube, mix in the following order:**

Between each addition, pipette mix 10-20 times.

Reagent	Volume
DNA sample from the previous step	60 µl
Ligation Buffer (LNB)	25 µl
NEBNext Quick T4 DNA Ligase	10 µl
Ligation Adapter (LA)	5 µl
**Total**	**100 µl**

6 **Ensure the reaction is thoroughly mixed by gently pipetting and spin down briefly.**

7 **Incubate the reaction for 10 minutes at room temperature.**

8 **Resuspend the AMPure XP Beads (AXP) by vortexing.**

9 **Add 40 µl of resuspended AMPure XP Beads (AXP) to the reaction and mix by flicking the tube.**

10 **Incubate on a Hula mixer (rotator mixer) for 5 minutes at room temperature.**

11 **Spin down the sample and pellet on a magnet. Keep the tube on the magnet, and pipette off the supernatant.**

12 **Wash the beads by adding either 250 µl Long Fragment Buffer (LFB) or 250 µl Short Fragment Buffer (SFB). Flick the beads to resuspend, spin down, then return the tube to the magnetic rack and allow the beads to pellet. Remove the supernatant using a pipette and discard.**

13 **Repeat the previous step.**

14 **Spin down and place the tube back on the magnet. Pipette off any residual supernatant. Allow to dry for ~30 seconds, but do not dry the pellet to the point of cracking.**

15 **Remove the tube from the magnetic rack and resuspend the pellet in 15 µl Elution Buffer (EB). Spin down and incubate for 10 minutes at room temperature. For high molecular weight DNA, incubating at 37°C can improve the recovery of long fragments.**

16 **Pellet the beads on a magnet until the eluate is clear and colourless, for at least 1 minute.**

17 **Remove and retain 15 µl of eluate containing the DNA library into a clean 1.5 ml Eppendorf DNA LoBind tube.**

Dispose of the pelleted beads

Quantify 1 µl of eluted sample using a Qubit fluorometer.

18 **Make up your library to 12 µl at 10-20 fmol.**

IMPORTANT

We recommend loading 10-20 fmol of this final prepared library onto the R10.4.1 flow cell.

Loading more than 20 fmol of DNA can reduce the rate of duplex read capture. Dilute the library in Elution Buffer if required.

END OF STEP

The prepared library is used for loading into the flow cell. Store the library on ice until ready to load.

TIP

Library storage recommendations

We recommend storing libraries in Eppendorf DNA LoBind tubes at**4°C for short-term** storage or repeated use, for example, re-loading flow cells between washes.
For single use and **long-term storage** of more than 3 months, we recommend storing libraries at**-80°C** in Eppendorf DNA LoBind tubes.

Optional Action
If quantities allow, the library may be diluted in Elution Buffer (EB) for splitting across multiple flow cells.

Depending on how many flow cells the library will be split across, more Elution Buffer (EB) than what is supplied in the kit will be required.

Priming and loading the SpotON flow cell

~10 minutes

**Materials**	• Flow Cell Flush (FCF)<br>• Flow Cell Tether (FCT)<br>• Library Solution (LIS)<br>• Library Beads (LIB)<br>• Sequencing Buffer (SB)
**Consumables**	• 1.5 ml Eppendorf DNA LoBind tubes

- SpotON Flow Cell
- Nuclease-free water (e.g. ThermoFisher, cat # AM9937)
- (Optional) Bovine Serum Albumin (BSA) (50 mg/ml) (e.g Invitrogen™ UltraPure™ BSA 50 mg/ml, AM2616)

Equipment

- MinION or GridION device
- P1000 pipette and tips
- P100 pipette and tips
- P20 pipette and tips
- P10 pipette and tips

IMPORTANT

Please note, this kit is only compatible with R10.4.1 flow cells (FLO-MIN114).

TIP

Priming and loading a flow cell

We recommend all new users watch the Priming and loading your flow cell video before your first run.

Using the Library Solution

We recommend using the Library Beads (LIB) for loading your library onto the flow cell for most sequencing experiments. However, if you have previously used water to load your library, you must use Library Solution (LIS) instead of water.
Note: Some customers have noticed that viscous libraries can be loaded more easily when not using Library Beads (LIB).

1 **Thaw the Sequencing Buffer (SB), Library Beads (LIB) or Library Solution (LIS, if using), Flow Cell Tether (FCT) and one tube of Flow Cell Flush (FCF) at room temperature. Mix by vortexing and spin down.**

IMPORTANT

For optimal sequencing performance and improved output on MinION R10.4.1 flow cells (FLO-MIN114), we recommend adding Bovine Serum Albumin (BSA) to the flow cell priming mix at a final concentration of 0.2 mg/ml.

Note: We do not recommend using any other albumin type (e.g. recombinant human serum albumin).

2 **To prepare the flow cell priming mix with BSA, add the following reagents directly to the tube of Flow Cell Flush (FCF), and mix by inverting the tube and pipette mix at room temperature:**

Reagent	Volume per flow cell
Flow Cell Flush (FCF)	1,170 µl
Bovine Serum Albumin (BSA) at 50 mg/ml	5 µl
Flow Cell Tether (FCT)	30 µl
**Final total volume in Flow Cell Flush (FCF) tube**	**1,205 µl**

3 **Open the MinION or GridION device lid and slide the flow cell under the clip. Press down firmly on the flow cell to ensure correct thermal and electrical contact.**

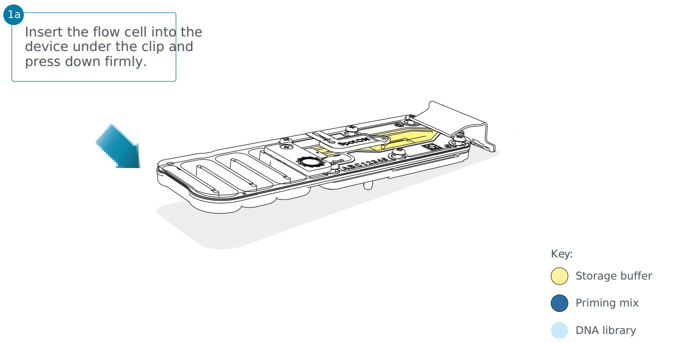

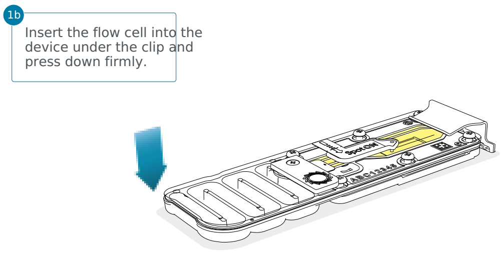

Optional Action
Complete a flow cell check to assess the number of pores available before loading the library.

This step can be omitted if the flow cell has been checked previously.

See the flow cell check instructions in the MinKNOW protocol for more information.

4 **Slide the flow cell priming port cover clockwise to open the priming port.**

IMPORTANT

Take care when drawing back buffer from the flow cell. Do not remove more than 20-30 µl, and make sure that the array of pores are covered by buffer at all times. Introducing air bubbles into the array can irreversibly damage pores.

5 **After opening the priming port, check for a small air bubble under the cover. Draw back a small volume to remove any bubbles:**

1. Set a P1000 pipette to 200 µl
2. Insert the tip into the priming port
3. Turn the wheel until the dial shows 220-230 ul, to draw back 20-30 ul, or until you can see a small volume of buffer entering the pipette tip
   Note: Visually check that there is continuous buffer from the priming port across the sensor array.

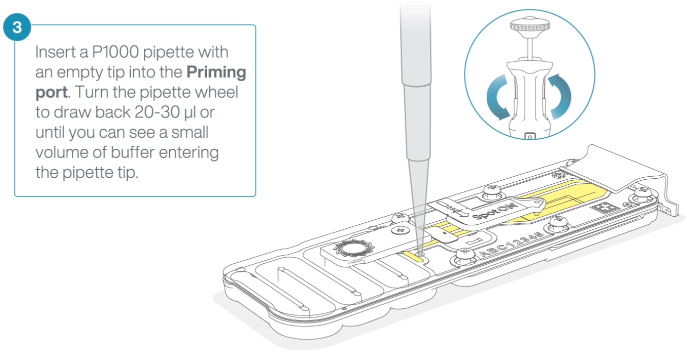

6 **Load 800 µl of the priming mix into the flow cell via the priming port, avoiding the introduction of air bubbles. Wait for 5 minutes. During this time, prepare the library for loading by following the steps below.**

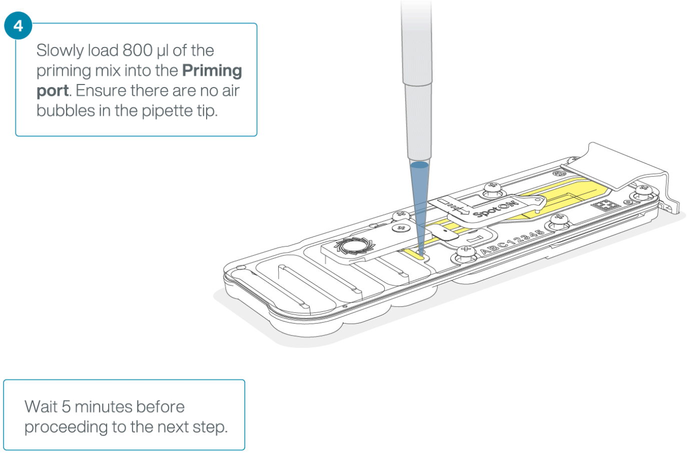

7 **Thoroughly mix the contents of the Library Beads (LIB) by pipetting.**

IMPORTANT

The Library Beads (LIB) tube contains a suspension of beads. These beads settle very quickly. It is vital that they are mixed immediately before use.

8 **In a new tube, prepare the library for loading as follows:**

Reagent	Volume per flow cell
Sequencing Buffer (SB)	37.5 µl
Library Beads (LIB) mixed immediately before use, or Library Solution (LIS), if using	25.5 µl
DNA library	12 µl
**Total**	**75 µl**

9 **Complete the flow cell priming:**

1. Gently lift the SpotON sample port cover to make the SpotON sample port accessible.
2. Load **200 µl** of the priming mix into the flow cell priming port (**not** the SpotON sample port), avoiding the introduction of air bubbles.

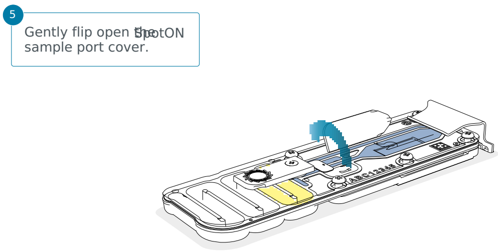

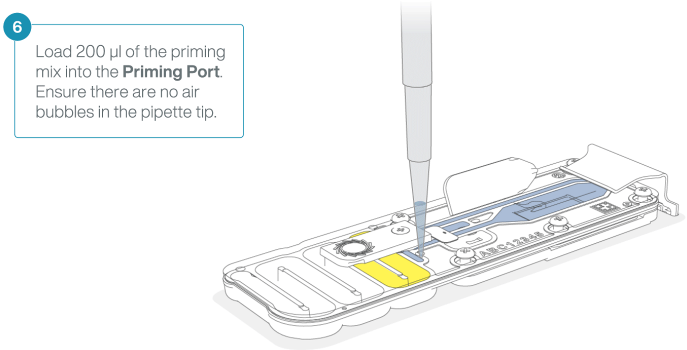

10 **Mix the prepared library gently by pipetting up and down just prior to loading.**

11 **Add 75 µl of the prepared library to the flow cell via the SpotON sample port in a dropwise fashion. Ensure each drop flows into the port before adding the next.**

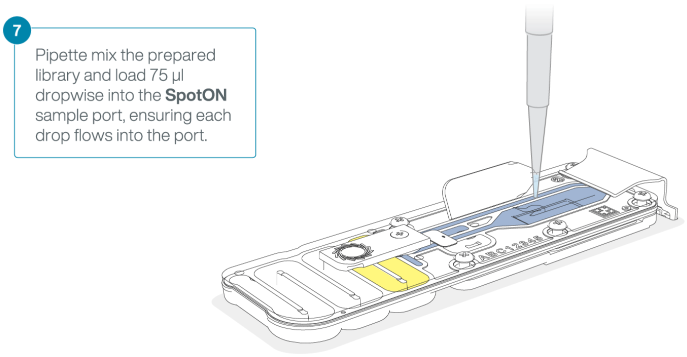

12 **Gently replace the SpotON sample port cover, making sure the bung enters the SpotON port, close the priming port and replace the MinION or GridION device lid.**

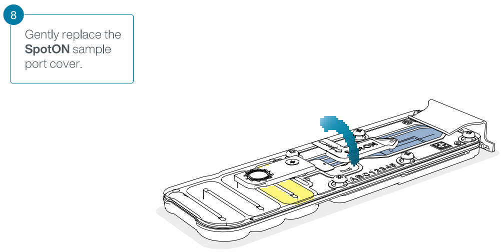

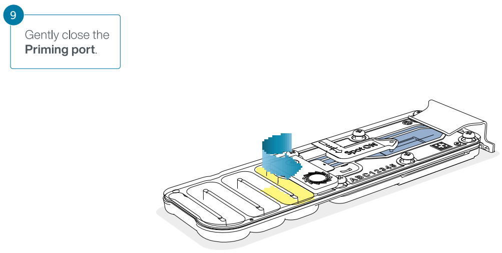

Data acquisition and duplex basecalling

Overview of nanopore data analysis

For a full overview of nanopore data analysis, which includes options for basecalling and post-basecalling analysis, please refer to the Data Analysis document.

Duplex basecalling

Kit 14 chemistry has improved duplex basecalling which requires basecalling on Dorado, using duplex tools after simplex basecalling has been performed on MinKNOW.

For detailed information on setting up your sequencing run, for both simplex and duplex basecalling, please see the Kit 14 sequencing and duplex basecalling info sheet.

Below explains the sequencing options available on MinKNOW and how to initially set up a simplex basecalling run.

How to start sequencing

The sequencing device control, data acquisition and real-time basecalling are carried out by the MinKNOW software. It is assumed you have already installed MinKNOW on your computer. There are multiple options for how to carry out sequencing:

1. Data acquisition and basecalling in real-time using MinKNOW on a computer

Follow the instructions in the MinKNOW protocol beginning from the "Starting a sequencing run" section until the end of the "Completing a MinKNOW run" section.

2. Data acquisition and basecalling in real-time using the GridION device

Follow the instructions in the GridION user manual.

3. Data acquisition and basecalling in real-time using the MinION Mk1C device

Follow the instructions in the MinION Mk1C user manual.

4. Data acquisition and basecalling in real-time using the PromethION device

Follow the instructions in the PromethION user manual or the PromethION 2 Solo user manual.

5. Data acquisition using MinKNOW on a computer and basecalling at a later time using MinKNOW or Guppy

Follow the instructions in the MinKNOW protocol beginning from the "Starting a sequencing run" section until the end of the "Completing a MinKNOW run" section. **When setting your experiment parameters, set the *Basecalling* tab to OFF.** After the sequencing experiment has completed, follow the instructions in the Post-run analysis section of the MinKNOW protocol or the Guppy protocol starting from the "Quick Start Guide for Guppy" section.

Downstream analysis

Post-basecalling analysis

There are several options for further analysing your basecalled data:

1. EPI2ME platform

The EPI2ME platform is a cloud-based data analysis service developed by Metrichor Ltd., a subsidiary of Oxford Nanopore Technologies. The EPI2ME platform offers a range of analysis workflows, e.g. for metagenomic identification, barcoding, alignment, and structural variant calling. The analysis requires no additional equipment or compute power, and provides an easy-to-interpret report with the results. For instructions on how to run an analysis workflow in EPI2ME, please follow the instructions in the EPI2ME protocol, beginning at the "Starting data analysis" step.

2. EPI2ME Labs tutorials and workflows

For more in-depth data analysis, Oxford Nanopore Technologies offers a range of bioinformatics tutorials and workflows available in EPI2ME Labs, which are available in the EPI2ME Labs section of the Community. The platform provides a vehicle where workflows deposited in GitHub by our Research and Applications teams can be showcased with descriptive texts, functional bioinformatics code and example data.

3. Research analysis tools

Oxford Nanopore Technologies' Research division has created a number of analysis tools, which are available in the Oxford Nanopore GitHub repository. The tools are aimed at advanced users, and contain instructions for how to install and run the software. They are provided as-is, with minimal support.

4. Community-developed analysis tools

If a data analysis method for your research question is not provided in any of the resources above, please refer to theBioinformatics section of the Resource centre. Numerous members of the Nanopore Community have developed their own tools and pipelines for analysing nanopore sequencing data, most of which are available on GitHub. Please be aware that these tools are not supported by Oxford Nanopore Technologies, and are not guaranteed to be compatible with the latest chemistry/software configuration.

Ending the experiment

Materials

- Flow Cell Wash Kit (EXP-WSH004)

1 **After your sequencing experiment is complete, if you would like to reuse the flow cell, please follow the Wash Kit instructions and store the washed flow cell at 2-8°C, OR**

The Flow Cell Wash Kit protocol is available on the Nanopore Community.

> **TIP**
>
> **We recommend you to wash the flow cell as soon as possible after you stop the run. However, if this is not possible, leave the flow cell on the device and wash it the next day.**

2 **Follow the returns procedure to flush out the flow cell ready to send back to Oxford Nanopore.**

Instructions for returning flow cells can be foundhere.

All flow cells must be flushed with deionised water before returning the product.

IMPORTANT

If you encounter issues or have questions about your sequencing experiment, please refer to the Troubleshooting Guide that can be found in the online version of this protocol.

Issues during DNA/RNA extraction and library preparation

Below is a list of the most commonly encountered issues, with some suggested causes and solutions.

We also have an FAQ section available on theNanopore Community Support section.

If you have tried our suggested solutions and the issue still persists, please contact Technical Support via email (support@nanoporetech.com) or via LiveChat in the Nanopore Community.

Low sample quality

Observation	Possible cause	Comments and actions
**Low DNA purity (Nanodrop reading for DNA OD 260/280 is <1.8 and OD 260/230 is <2.0–2.2)**	The DNA extraction method does not provide the required purity	The effects of contaminants are shown in the Contaminants Know-how piece. Please try an alternative extraction method that does not result in contaminant carryover.<br><br>Consider performing an additional SPRI clean-up step.
**Low RNA integrity (RNA integrity number <9.5 RIN, or the rRNA band is shown as a smear on the gel)**	The RNA degraded during extraction	Try a different RNA extraction method. For more info on RIN, please see the RNA Integrity Number Know-how piece.
**RNA has a shorter than expected fragment length**	The RNA degraded during extraction	Try a different RNA extraction method. For more info on RIN, please see the RNA Integrity Number Know-how piece.<br><br>We recommend working in an RNase-free environment, and to keep your lab equipment RNase-free when working with RNA.

Low DNA recovery after AMPure bead clean-up

Observation	Possible cause	Comments and actions

Observation	Possible cause	Comments and actions
**Low recovery**	DNA loss due to a lower than intended AMPure beads-to-sample ratio	1. AMPure beads settle quickly, so ensure they are well resuspended before adding them to the sample.<br><br>2. When the AMPure beads-to-sample ratio is lower than 0.4:1, DNA fragments of any size will be lost during the clean-up.
**Low recovery**	DNA fragments are shorter than expected	The lower the AMPure beads-to-sample ratio, the more stringent the selection against short fragments. Please always determine the input DNA length on an agarose gel (or other gel electrophoresis methods) and then calculate the appropriate amount of AMPure beads to use.<br>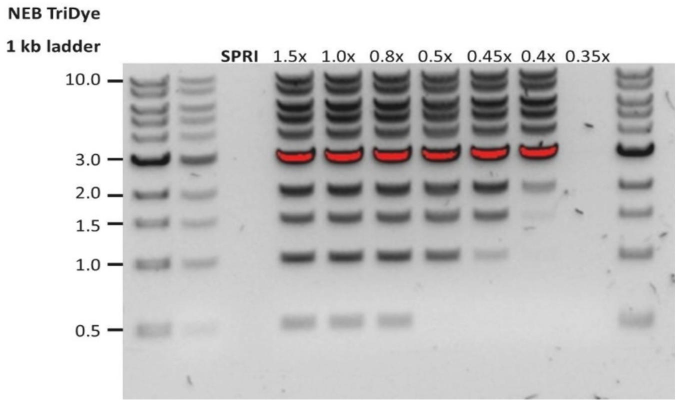
**Low recovery after end-prep**	The wash step used ethanol <70%	DNA will be eluted from the beads when using ethanol <70%. Make sure to use the correct percentage.

Issues during the sequencing run

Below is a list of the most commonly encountered issues, with some suggested causes and solutions.

We also have an FAQ section available on the Nanopore Community Support section.

If you have tried our suggested solutions and the issue still persists, please contact Technical Support via email (support@nanoporetech.com) or via LiveChat in the Nanopore Community.

Fewer pores at the start of sequencing than after Flow Cell Check

Observation	Possible cause	Comments and actions
**MinKNOW reported a lower number of pores at the start of sequencing than the number reported by the Flow Cell Check**	An air bubble was introduced into the nanopore array	After the Flow Cell Check it is essential to remove any air bubbles near the priming port before priming the flow cell. If not removed, the air bubble can travel to the nanopore array and irreversibly damage the nanopores that have been exposed to air. The best practice to prevent this from happening is demonstrated in this video.
**MinKNOW reported a lower number of pores at the start of sequencing than the number reported by the Flow Cell Check**	The flow cell is not correctly inserted into the device	Stop the sequencing run, remove the flow cell from the sequencing device and insert it again, checking that the flow cell is firmly seated in the device and that it has reached the target temperature. If applicable, try a different position on the device (GridION/PromethION).
**MinKNOW reported a lower number of pores at the start of sequencing than the number reported by the Flow Cell Check**	Contaminations in the library damaged or blocked the pores	The pore count during the Flow Cell Check is performed using the QC DNA molecules present in the flow cell storage buffer. At the start of sequencing, the library itself is used to estimate the number of active pores. Because of this, variability of about 10% in the number of pores is expected. A significantly lower pore count reported at the start of sequencing can be due to contaminants in the library that have damaged the membranes or blocked the pores. Alternative DNA/RNA extraction or purification methods may be needed to improve the purity of the input material. The effects of contaminants are shown in the Contaminants Know-how piece. Please try an alternative extraction method that does not result in contaminant carryover.

MinKNOW script failed

Observation	Possible cause	Comments and actions
**MinKNOW shows "Script failed"**		Restart the computer and then restart MinKNOW. If the issue persists, please collect the MinKNOW log files and contact Technical Support.

Pore occupancy below 40%

Observation	Possible cause	Comments and actions
**Pore occupancy <40%**	Not enough library was loaded on the flow cell	Ensure the correct volume and concentration as stated on the appropriate protocol for your sequencing library is loaded onto the flow cell. Please quantify the library before loading and calculate fmols using tools like the Promega Biomath Calculator, choosing "dsDNA: µg to fmol"

Observation	Possible cause	Comments and actions
**Pore occupancy close to 0**	The Ligation Sequencing Kit was used, and sequencing adapters did not ligate to the DNA	Make sure to use the NEBNext Quick Ligation Module (E6056) and Oxford Nanopore Technologies Ligation Buffer (LNB, provided in the SQK-LSK110 kit) at the sequencing adapter ligation step, and use the correct amount of each reagent. A Lambda control library can be prepared to test the integrity of the third-party reagents.
**Pore occupancy close to 0**	The Ligation Sequencing Kit was used, and ethanol was used instead of LFB or SFB at the wash step after sequencing adapter ligation	Ethanol can denature the motor protein on the sequencing adapters. Make sure the LFB or SFB buffer was used after ligation of sequencing adapters.
**Pore occupancy close to 0**	No tether on the flow cell	Tethers are adding during flow cell priming (FLT tube for Kit 9, 10, 11, and FCT for Kit 14). Make sure FLT/FCT was added to the buffer (FB for Kit 9, 10, 11, and FCF for Kit 14) before priming.

Shorter than expected read length

Observation	Possible cause	Comments and actions
**Shorter than expected read length**	Unwanted fragmentation of DNA sample	Read length reflects input DNA fragment length. Input DNA can be fragmented during extraction and library prep.<br><br>1. Please review the Extraction Methods in the Nanopore Community for best practice for extraction.<br>2. Visualise the input DNA fragment length distribution on an agarose gel before proceeding to the library prep.<br><br>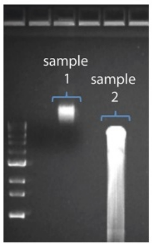<br><br><br>In the image above, Sample 1 is of high molecular weight, whereas Sample 2 has been fragmented.<br><br>3. During library prep, avoid pipetting and vortexing when mixing reagents. Flicking or inverting the tube is sufficient.

Large proportion of recovering pores

Observation	Possible cause	Comments and actions

Observation	Possible cause	Comments and actions
**Large proportion of recovering pores (shown as dark blue in the channels panel and duty time plot)**	Contaminants are present in the sample	Some contaminants can be cleared from the pores by the unblocking function built into MinKNOW. If this is successful, the pore status will change to "single pores". If the portion of recovering pores (unavailable pores in the extended view) stays large or increases:<br><br>1. A nuclease flush using the Flow Cell Wash Kit (EXP-WSH004) can be performed, or<br>2. Run several cycles of PCR to try and dilute any contaminants that may be causing problems.<br><br>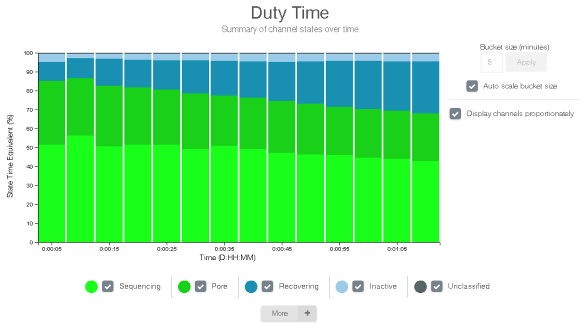<br><br>The duty time plot above shows an increasing proportion of "recovering" pores over the course of a sequencing experiment

Large proportion of inactive pores

Observation	Possible cause	Comments and actions
**Large proportion of inactive pores (shown as light blue in the channels panel and duty time plot. Pores or membranes are irreversibly damaged)**	Air bubbles have been introduced into the flow cell	Air bubbles introduced through flow cell priming and library loading can irreversibly damage the pores. Watch the Priming and loading your flow cell video for best practice
**Large proportion of inactive pores**	Certain compounds co-purified with DNA	Known compounds, include polysaccharides, typically associate with plant genomic DNA.<br><br>1. Please refer to the Plant leaf DNA extraction method.<br>2. Clean-up using the QIAGEN PowerClean Pro kit.<br>3. Perform a whole genome amplification with the original gDNA sample using the QIAGEN REPLI-g kit.
**Large proportion of inactive pores**	Contaminants are present in the sample	The effects of contaminants are shown in the Contaminants Know-how piece. Please try an alternative extraction method that does not result in contaminant carryover.

Reduction in sequencing speed and q-score later into the run

Observation	Possible cause	Comments and actions
**Reduction in sequencing speed and q-score later into the run**	Fast fuel consumption is typically seen when the flow cell is overloaded with library (please see the appropriate protocol for your DNA library to see the recommendation).	Add more fuel to the flow cell by following the instructions in the MinKNOW protocol. In future experiments, load lower amounts of library to the flow cell.

Temperature fluctuation

Observation	Possible cause	Comments and actions
**Temperature fluctuation**	The flow cell has lost contact with the device	Check that there is a heat pad covering the metal plate on the back of the flow cell. Re-insert the flow cell and press it down to make sure the connector pins are firmly in contact with the device. If the problem persists, please contact Technical Services.

Failed to reach target temperature

Observation	Possible cause	Comments and actions
**MinKNOW shows "Failed to reach target temperature"**	The instrument was placed in a location that is colder than normal room temperature, or a location with poor ventilation (which leads to the flow cells overheating)	MinKNOW has a default timeframe for the flow cell to reach the target temperature. Once the timeframe is exceeded, an error message will appear and the sequencing experiment will continue. However, sequencing at an incorrect temperature may lead to a decrease in throughput and lower q-scores. Please adjust the location of the sequencing device to ensure that it is placed at room temperature with good ventilation, then re-start the process in MinKNOW. Please refer to this FAQ for more information on MinION Mk 1B temperature control.

Guppy – no input .fast5 was found or basecalled

Observation	Possible cause	Comments and actions
**No input .fast5 was found or basecalled**	*input_path* did not point to the .fast5 file location	The *--input_path* has to be followed by the full file path to the .fast5 files to be basecalled, and the location has to be accessible either locally or remotely through SSH.
**No input .fast5 was found or basecalled**	The .fast5 files were in a subfolder at the *input_path* location	To allow Guppy to look into subfolders, add the*--recursive* flag to the command

Guppy – no Pass or Fail folders were generated after basecalling

Observation	Possible cause	Comments and actions
**No Pass or Fail folders were generated after basecalling**	The *--qscore_filtering* flag was not included in the command	The *--qscore_filtering* flag enables filtering of reads into Pass and Fail folders inside the output folder, based on their strand q-score. When performing live basecalling in MinKNOW, a q-score of 7 (corresponding to a basecall accuracy of ~80%) is used to separate reads into Pass and Fail folders.

Guppy – unusually slow processing on a GPU computer

Observation	Possible cause	Comments and actions
**Unusually slow processing on a GPU computer**	The *--device* flag wasn't included in the command	The *--device* flag specifies a GPU device to use for accelerate basecalling. If not included in the command, GPU will not be used. GPUs are counted from zero. An example is *--device cuda:0 cuda:1*, when 2 GPUs are specified to use by the Guppy command.
