# Supplementary material for: Genomic DNA extraction optimization and validation for genome sequencing using the marine gastropod Kellet’s whelk
Source: PeerJ. 2023 Dec 6;11:e16510. doi: 10.7717/peerj.16510 (PMC10710129; doi:10.7717/peerj.16510)
Supplement: Supplemental Information 7 [file peerj-11-16510-s007.zip › Nanopore MinION Rapid Sequencing Kit.pdf]

Overview of the protocol

IMPORTANT

This is a Legacy product

This kit is soon to be discontinued and we recommend all customers to upgrade to the latest chemistry for their relevant kit which is available on the Store. If customers require further support for any ongoing critical experiments using a Legacy product, please contact Customer Support via email: support@nanoporetech.com.

Rapid Sequencing Kit features

This kit is recommended for users who:

- require a short preparation time
- have limited access to laboratory equipment

Introduction to Rapid Sequencing protocol (SQK-RAD004)

This protocol describes the step-by-step instructions to complete a rapid sequencing of genomic DNA using the Rapid Sequencing Kit (SQK-RAD004). It is highly recommended that a Lambda control experiment is completed first to become familiar with the technology.

Steps in the sequencing workflow:

Prepare for your experiment

You will need to:
- Extract your DNA, and check its length, quantity and purity.
The quality checks performed during the protocol are essential in ensuring experimental success.
- Ensure you have your sequencing kit, the correct equipment and third-party reagents
- If not already installed, download the software for acquiring and analysing your data
- Check your flow cell(s) to ensure it has enough pores for a good sequencing run

Library preparation

You will need to:
- Tagment your DNA using the Fragmentation Mix in the kit
- Attach sequencing adapters supplied in the kit to the DNA ends
- Prime the flow cell, and load your DNA library into the flow cell

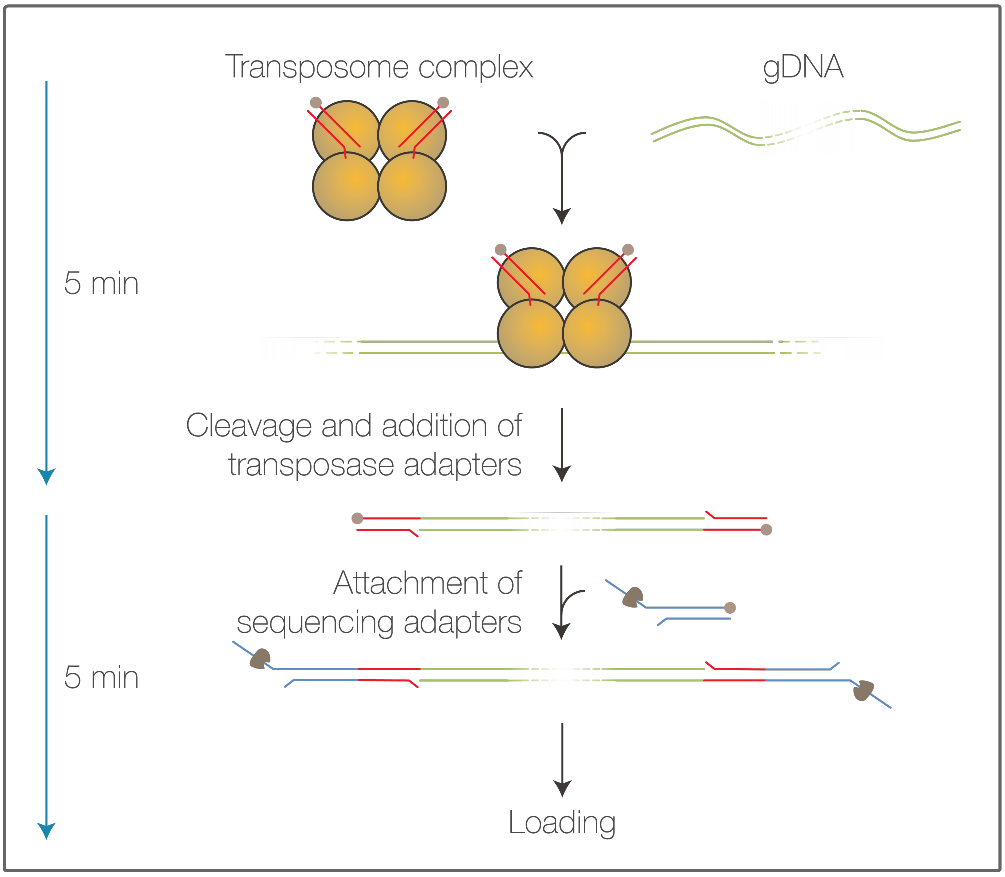

Sequencing and analysis

You will need to:
- Start a sequencing run using the MinKNOW software, which will collect raw data from the device and convert it into basecalled reads
- ***optional*** Start the EPI2ME software and select a workflow for further analysis, e.g. metagenomic analysis or drug resistance mapping

IMPORTANT

Compatibility of this protocol

This protocol should only be used in combination with:

- Rapid Sequencing Kit (SQK-RAD004)
- R9.4.1 (FLO-MIN106) flow cells
- Flow Cell Wash Kit (EXP-WSH004)

Equipment and consumables

Materials

- ~400 ng high molecular weight genomic DNA

	• Rapid Sequencing Kit (SQK-RAD004)<br>• Flow Cell Priming Kit (EXP-FLP002)
**Consumables**	• 1.5 ml Eppendorf DNA LoBind tubes<br>• 0.2 ml thin-walled PCR tubes<br>• Nuclease-free water (e.g. ThermoFisher, AM9937)
**Equipment**	• Microfuge<br>• P1000 pipette and tips<br>• P100 pipette and tips<br>• P20 pipette and tips<br>• P10 pipette and tips<br>• P2 pipette and tips<br>• Timer<br>• Thermal cycler or heat block at 30°C and 80°C
**Optional Equipment**	• Qubit fluorometer (or equivalent for QC check)

For this protocol, you will need ~400 ng high molecular weight genomic DNA

Input DNA

How to QC your input DNA

It is important that the input DNA meets the quantity and quality requirements. Using too little or too much DNA, or DNA of poor quality (e.g. highly fragmented or containing RNA or chemical contaminants) can affect your library preparation.

For instructions on how to perform quality control of your DNA sample, please read the Input DNA/RNA QC protocol.

Chemical contaminants

Depending on how the DNA is extracted from the raw sample, certain chemical contaminants may remain in the purified DNA, which can affect library preparation efficiency and sequencing quality. Read more about contaminants on the Contaminants page of the Community.

Rapid Sequencing Kit contents

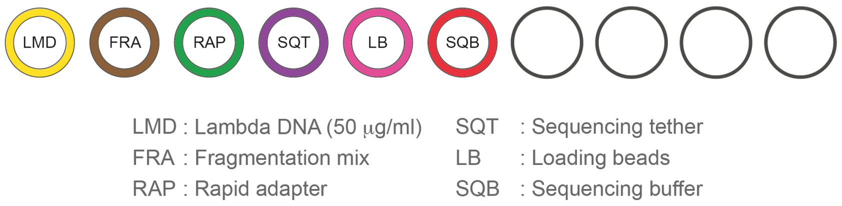

Name	Acronym	Cap colour	No. of vials	Fill volume per vial (µl)
Lambda DNA	LMD	Yellow	1	88

Name	Acronym	Cap colour	No. of vials	Fill volume per vial (µl)
Fragmentation Mix	FRA	Brown	1	30
Rapid Adapter	RAP	Green	1	10
Sequencing Tether	SQT	Purple	1	10
Loading Beads	LB	Pink	1	360
Sequencing Buffer	SQB	Red	1	300

IMPORTANT

Please note that the Sequencing Tether (SQT) tube will NOT be used in this protocol.

Flow Cell Priming Kit contents (EXP-FLP002)

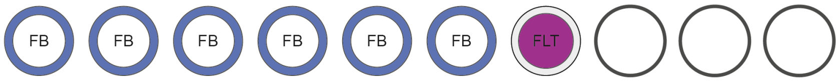

FLB : Flush buffer
FLT : Flush tether

Name	Acronym	Cap colour	No. of vials	Fill volume per vial (µl)
Flush Buffer	FB	Blue	6	1,170
Flush Tether	FLT	Purple	1	200

Computer requirements and software

MinION Mk1B IT requirements

Sequencing on a MinION Mk1B requires a high-spec computer or laptop to keep up with the rate of data acquisition. Read more in the MinION IT Requirements document.

MinION Mk1C IT requirements

The MinION Mk1C contains fully-integrated compute and screen, removing the need for any accessories to generate and analyse nanopore data. Read more in the MinION Mk1C IT requirements document.

Software for nanopore sequencing

MinKNOW

The MinKNOW software controls the nanopore sequencing device, collects sequencing data in real time and processes it into basecalls. You will be using MinKNOW for every sequencing experiment. MinKNOW can also demultiplex reads by barcode, and

basecall/demultiplex data after a sequencing run has completed.

MinKNOW use

For instructions on how to run the MinKNOW software, please refer to the relevant section in the MinKNOW protocol.

EPI2ME (optional)

The EPI2ME cloud-based platform performs further analysis of basecalled data, for example alignment to the Lambda genome, barcoding, or taxonomic classification. You will use the EPI2ME platform *only* if you would like further analysis of your data post-basecalling.

EPI2ME installation and use

For instructions on how to create an EPI2ME account and install the EPI2ME Desktop Agent, please refer to the EPI2ME Platform protocol.

Guppy (optional)

The Guppy command-line software can be used for basecalling and demultiplexing reads by barcode instead of MinKNOW. You can use it if you would like to re-analyse old data, or integrate basecalling into your analysis pipeline.

Guppy installation and use

If you would like to use the Guppy software, please refer to the Guppy protocol.

Check your flow cell

We highly recommend that you check the number of pores in your flow cell prior to starting a sequencing experiment. This should be done within three months of purchasing for MinION/GridION/PromethION flow cells, or within four weeks of purchasing for Flongle flow cells. Oxford Nanopore Technologies will replace any flow cell with fewer than the number of pores in the table below, when the result is reported within two days of performing the flow cell check, and when the storage recommendations have been followed. To do the flow cell check, please follow the instructions in the Flow Cell Check document.

Flow cell	Minimum number of active pores covered by warranty
Flongle Flow Cell	50
MinION/GridION Flow Cell	800
PromethION Flow Cell	5000

Library preparation

~10 minutes

**Materials**	• ~400 ng high molecular weight genomic DNA<br>• Fragmentation Mix (FRA)<br>• Rapid Adapter (RAP)
**Consumables**	• 0.2 ml thin-walled PCR tubes<br>• Nuclease-free water (e.g. ThermoFisher, AM9937)

**Equipment**	

- Thermal cycler or heat block at 30°C and 80°C
- P2 pipette and tips
- P10 pipette and tips

DNA tagmentation

1 **Thaw kit components at room temperature, spin down briefly using a microfuge and mix by pipetting as indicated by the table below:**

Reagent	1. Thaw at room temperature	2. Briefly spin down	3. Mix well by pipetting
Fragmentation Mix (FRA)	Not frozen	✓	✓
Rapid Adapter (RAP)	Not frozen	✓	✓
Sequencing Buffer (SQB)	✓	✓	✓*
Loading Beads (LB)	✓	✓	Mix by pipetting or vortexing immediately before use
Flush Buffer (FLB) - 1 tube	✓	✓	✓*
Flush Tether (FLT)	✓	✓	✓

*Vortexing, followed by a brief spin in a microfuge, is recommended for Sequencing Buffer (SQB) and Flush Buffer (FLB).

Please note that the Sequencing Tether (SQT) tube will NOT be used in this protocol. It is provided in the kit for potential future product compatibility.

2 **Once thawed, keep all the kit components on ice.**

3 **Prepare the DNA in nuclease-free water.**

- Transfer ~400 ng genomic DNA into a DNA LoBind tube
- Adjust the volume to 7.5 µl with nuclease-free water
- Mix by flicking the tube to avoid unwanted shearing
- Spin down briefly in a microfuge

4 **In a 0.2 ml thin-walled PCR tube, mix the following:**

Reagent	Volume
400 ng template DNA	7.5 µl
FRA	2.5 µl
**Total**	**10 µl**

5 **Mix gently by flicking the tube, and spin down.**

6 **Incubate the tube at 30°C for 1 minute and then at 80°C for 1 minute. Briefly put the tube on ice to cool it down.**

> **TIP**
>
> **If heat blocks are used instead of a thermal cycler, incubation at both temperatures should be extended to 2 minutes.**

400 ng tagmented DNA in 10 µl is taken into the next step.

Adapter attachment

7 **Add 1 µl of RAP to the tube.**

8 **Mix gently by flicking the tube, and spin down.**

9 **Incubate the reaction for 5 minutes at room temperature.**

> **END OF STEP**
>
> **The prepared DNA library is used for loading into the flow cell. Store the library on ice until ready to load.**

Priming and loading the SpotON Flow Cell

~10 minutes

**Materials**	• Flow Cell Priming Kit (EXP-FLP002)<br>• Sequencing Buffer (SQB)<br>• Loading Beads (LB)
**Consumables**	• 1.5 ml Eppendorf DNA LoBind tubes<br>• Nuclease-free water (e.g. ThermoFisher, cat # AM9937)
**Equipment**	• MinION Mk1B<br>• SpotON Flow Cell<br>• P1000 pipette and tips<br>• P100 pipette and tips<br>• P20 pipette and tips<br>• P10 pipette and tips

IMPORTANT

Please note that the Sequencing Tether (SQT) tube will NOT be used in this protocol.

TIP

Priming and loading a flow cell

We recommend all new users watch the Priming and loading your flow cell video before your first run.

1 **Thaw the Sequencing Buffer (SQB), Loading Beads (LB), Flush Tether (FLT) and one tube of Flush Buffer (FB) at room temperature before mixing the reagents by vortexing, and spin down at room temperature.**

2 **To prepare the flow cell priming mix, add 30 µl of thawed and mixed Flush Tether (FLT) directly to the tube of thawed and mixed Flush Buffer (FB), and mix by vortexing at room temperature.**

3 **Open the MinION device lid and slide the flow cell under the clip.**

Press down firmly on the flow cell to ensure correct thermal and electrical contact.

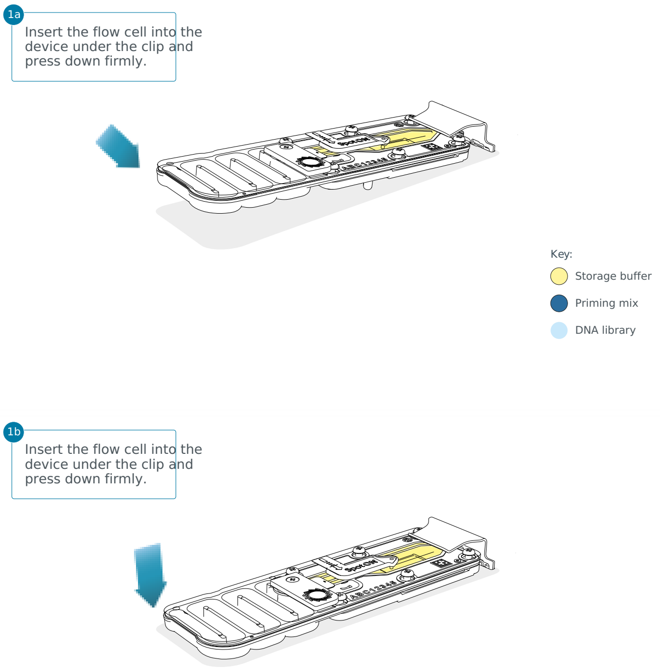

Optional Action
Complete a flow cell check to assess the number of pores available before loading the library.

This step can be omitted if the flow cell has been checked previously.

See the flow cell check instructions in the MinKNOW protocol for more information.

4 Slide the priming port cover clockwise to open the priming port.

IMPORTANT

Take care when drawing back buffer from the flow cell. Do not remove more than 20-30 µl, and make sure that the array of pores are covered by buffer at all times. Introducing air bubbles into the array can irreversibly damage pores.

5 After opening the priming port, check for a small air bubble under the cover. Draw back a small volume to remove any bubbles:

1. Set a P1000 pipette to 200 µl
2. Insert the tip into the priming port
3. Turn the wheel until the dial shows 220-230 ul, to draw back 20-30 ul, or until you can see a small volume of buffer entering the pipette tip
   Note: Visually check that there is continuous buffer from the priming port across the sensor array.

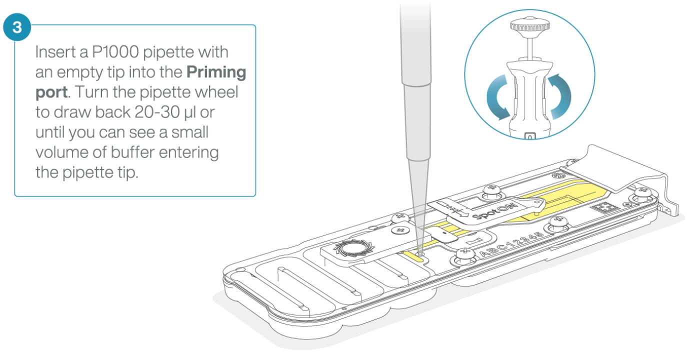

6 **Load 800 µl of the priming mix into the flow cell via the priming port, avoiding the introduction of air bubbles. Wait for 5 minutes. During this time, prepare the library for loading by following the steps below.**

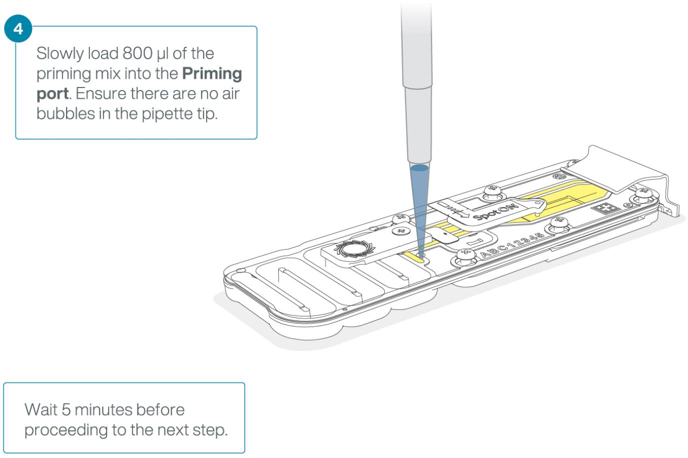

7 **Thoroughly mix the contents of the Loading Beads (LB) tubes by vortexing.**

8 **In a new tube, prepare the library for loading as follows:**

Reagent	Volume per flow cell
Sequencing Buffer (SQB)	34 µl
Loading Beads (LB), mixed immediately before use	25.5 µl
Nuclease-free water	4.5 µl
DNA library	11 µl
**Total**	**75 µl**

Note: Load the library onto the flow cell immediately after adding the Sequencing Buffer (SQB) and Loading Beads (LB) because the fuel in the buffer will start to be consumed by the adapter.

IMPORTANT

The Loading Beads (LB) tube contains a suspension of beads. These beads settle very quickly. It is vital that they are mixed immediately before use.

9 **Complete the flow cell priming:**

1. Gently lift the SpotON sample port cover to make the SpotON sample port accessible.
2. Load **200 µl** of the priming mix into the flow cell priming port (**not** the SpotON sample port), avoiding the introduction of air bubbles.

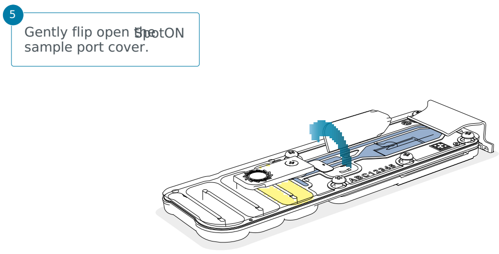

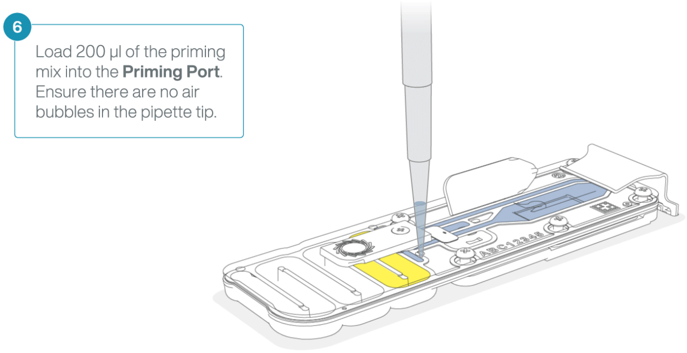

10 **Mix the prepared library gently by pipetting up and down just prior to loading.**

11 **Add 75 µl of the prepared library to the flow cell via the SpotON sample port in a dropwise fashion. Ensure each drop flows into the port before adding the next.**

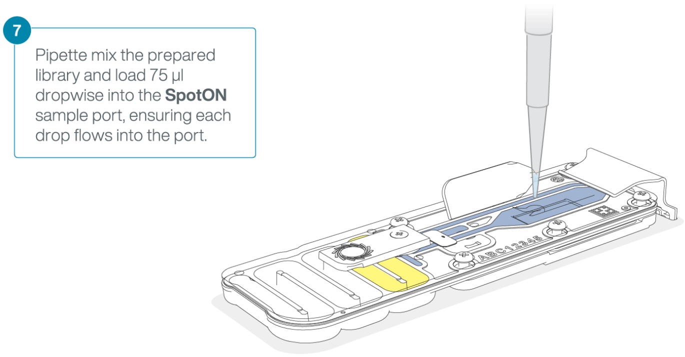

12 **Gently replace the SpotON sample port cover, making sure the bung enters the SpotON port, close the priming port and replace the MinION device lid.**

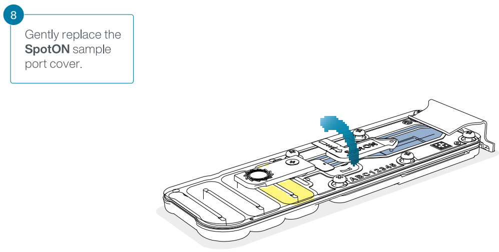

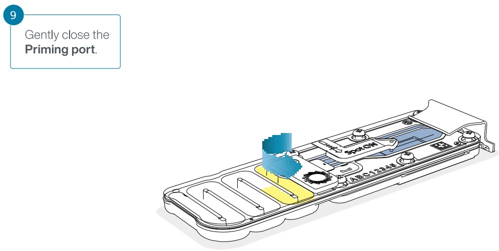

Data acquisition and basecalling

Overview of nanopore data analysis

For a full overview of nanopore data analysis, which includes options for basecalling and post-basecalling analysis, please refer to the Data Analysis document.

How to start sequencing

The sequencing device control, data acquisition and real-time basecalling are carried out by the MinKNOW software. It is assumed you have already installed MinKNOW on your computer. There are multiple options for how to carry out sequencing:

1. Data acquisition and basecalling in real-time using MinKNOW on a computer

Follow the instructions in the MinKNOW protocol beginning from the "Starting a sequencing run" section until the end of the "Completing a MinKNOW run" section.

2. Data acquisition and basecalling in real-time using the GridION device

Follow the instructions in the GridION user manual.

3. Data acquisition and basecalling in real-time using the MinION Mk1C device

Follow the instructions in the MinION Mk1C user manual.

4. Data acquisition and basecalling in real-time using the PromethION device

Follow the instructions in the PromethION user manual or the PromethION 2 Solo user manual.

5. Data acquisition using MinKNOW on a computer and basecalling at a later time using MinKNOW or Guppy

Follow the instructions in the MinKNOW protocol beginning from the "Starting a sequencing run" section until the end of the "Completing a MinKNOW run" section. **When setting your experiment parameters, set the *Basecalling* tab to OFF.** After the sequencing experiment has completed, follow the instructions in the Post-run analysis section of the MinKNOW protocol or the Guppy protocol starting from the "Quick Start Guide for Guppy" section.

Downstream analysis

Post-basecalling analysis

There are several options for further analysing your basecalled data:

1. EPI2ME platform

The EPI2ME platform is a cloud-based data analysis service developed by Metrichor Ltd., a subsidiary of Oxford Nanopore Technologies. The EPI2ME platform offers a range of analysis workflows, e.g. for metagenomic identification, barcoding, alignment, and structural variant calling. The analysis requires no additional equipment or compute power, and provides an easy-to-interpret

report with the results. For instructions on how to run an analysis workflow in EPI2ME, please follow the instructions in the EPI2ME protocol, beginning at the "Starting data analysis" step.

2. EPI2ME Labs tutorials and workflows

For more in-depth data analysis, Oxford Nanopore Technologies offers a range of bioinformatics tutorials and workflows available in EPI2ME Labs, which are available in the EPI2ME Labs section of the Community. The platform provides a vehicle where workflows deposited in GitHub by our Research and Applications teams can be showcased with descriptive texts, functional bioinformatics code and example data.

3. Research analysis tools

Oxford Nanopore Technologies' Research division has created a number of analysis tools, which are available in the Oxford Nanopore GitHub repository. The tools are aimed at advanced users, and contain instructions for how to install and run the software. They are provided as-is, with minimal support.

4. Community-developed analysis tools

If a data analysis method for your research question is not provided in any of the resources above, please refer to the Bioinformatics section of the Resource centre. Numerous members of the Nanopore Community have developed their own tools and pipelines for analysing nanopore sequencing data, most of which are available on GitHub. Please be aware that these tools are not supported by Oxford Nanopore Technologies, and are not guaranteed to be compatible with the latest chemistry/software configuration.

Ending the experiment

Materials

- Flow Cell Wash Kit (EXP-WSH004)

1 **After your sequencing experiment is complete, if you would like to reuse the flow cell, please follow the Wash Kit instructions and store the washed flow cell at 2-8°C, OR**

The Flow Cell Wash Kit protocol is available on the Nanopore Community.

> **TIP**
>
> **We recommend you to wash the flow cell as soon as possible after you stop the run. However, if this is not possible, leave the flow cell on the device and wash it the next day.**

2 **Follow the returns procedure to flush out the flow cell ready to send back to Oxford Nanopore.**

Instructions for returning flow cells can be foundhere.

All flow cells must be flushed with deionised water before returning the product.

IMPORTANT

If you encounter issues or have questions about your sequencing experiment, please refer to the Troubleshooting Guide that can be found in the online version of this protocol.

Issues during DNA/RNA extraction and library preparation

Below is a list of the most commonly encountered issues, with some suggested causes and solutions.

We also have an FAQ section available on the Nanopore Community Support section.

If you have tried our suggested solutions and the issue still persists, please contact Technical Support via email (support@nanoporetech.com) or via LiveChat in the Nanopore Community.

Low sample quality

Observation	Possible cause	Comments and actions
**Low DNA purity (Nanodrop reading for DNA OD 260/280 is <1.8 and OD 260/230 is <2.0–2.2)**	The DNA extraction method does not provide the required purity	The effects of contaminants are shown in the Contaminants Know-how piece. Please try an alternative extraction method that does not result in contaminant carryover.<br><br>Consider performing an additional SPRI clean-up step.
**Low RNA integrity (RNA integrity number <9.5 RIN, or the rRNA band is shown as a smear on the gel)**	The RNA degraded during extraction	Try a different RNA extraction method. For more info on RIN, please see the RNA Integrity Number Know-how piece.
**RNA has a shorter than expected fragment length**	The RNA degraded during extraction	Try a different RNA extraction method. For more info on RIN, please see the RNA Integrity Number Know-how piece.<br><br>We recommend working in an RNase-free environment, and to keep your lab equipment RNase-free when working with RNA.

Low DNA recovery after AMPure bead clean-up

Observation	Possible cause	Comments and actions
**Low recovery**	DNA loss due to a lower than intended AMPure beads-to-sample ratio	1. AMPure beads settle quickly, so ensure they are well resuspended before adding them to the sample.<br><br>2. When the AMPure beads-to-sample ratio is lower than 0.4:1, DNA fragments of any size will be lost during the clean-up.

Observation	Possible cause	Comments and actions
**Low recovery**	DNA fragments are shorter than expected	The lower the AMPure beads-to-sample ratio, the more stringent the selection against short fragments. Please always determine the input DNA length on an agarose gel (or other gel electrophoresis methods) and then calculate the appropriate amount of AMPure beads to use.<br>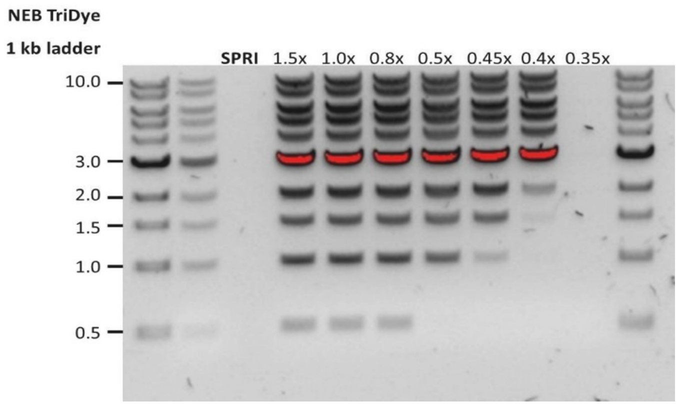
**Low recovery after end-prep**	The wash step used ethanol <70%	DNA will be eluted from the beads when using ethanol <70%. Make sure to use the correct percentage.

Issues during the sequencing run

Below is a list of the most commonly encountered issues, with some suggested causes and solutions.

We also have an FAQ section available on the Nanopore Community Support section.

If you have tried our suggested solutions and the issue still persists, please contact Technical Support via email (support@nanoporetech.com) or via LiveChat in the Nanopore Community.

Fewer pores at the start of sequencing than after Flow Cell Check

Observation	Possible cause	Comments and actions
**MinKNOW reported a lower number of pores at the start of sequencing than the number reported by the Flow Cell Check**	An air bubble was introduced into the nanopore array	After the Flow Cell Check it is essential to remove any air bubbles near the priming port before priming the flow cell. If not removed, the air bubble can travel to the nanopore array and irreversibly damage the nanopores that have been exposed to air. The best practice to prevent this from happening is demonstrated in this video.

Observation	Possible cause	Comments and actions
**MinKNOW reported a lower number of pores at the start of sequencing than the number reported by the Flow Cell Check**	The flow cell is not correctly inserted into the device	Stop the sequencing run, remove the flow cell from the sequencing device and insert it again, checking that the flow cell is firmly seated in the device and that it has reached the target temperature. If applicable, try a different position on the device (GridION/PromethION).
**MinKNOW reported a lower number of pores at the start of sequencing than the number reported by the Flow Cell Check**	Contaminations in the library damaged or blocked the pores	The pore count during the Flow Cell Check is performed using the QC DNA molecules present in the flow cell storage buffer. At the start of sequencing, the library itself is used to estimate the number of active pores. Because of this, variability of about 10% in the number of pores is expected. A significantly lower pore count reported at the start of sequencing can be due to contaminants in the library that have damaged the membranes or blocked the pores. Alternative DNA/RNA extraction or purification methods may be needed to improve the purity of the input material. The effects of contaminants are shown in the Contaminants Know-how piece. Please try an alternative extraction method that does not result in contaminant carryover.

MinKNOW script failed

Observation	Possible cause	Comments and actions
**MinKNOW shows "Script failed"**		Restart the computer and then restart MinKNOW. If the issue persists, please collect the MinKNOW log files and contact Technical Support.

Pore occupancy below 40%

Observation	Possible cause	Comments and actions
**Pore occupancy <40%**	Not enough library was loaded on the flow cell	5–50 fmol of good quality library can be loaded on to a MinION Mk1B/GridION flow cell. Please quantify the library before loading and calculate mols using tools like the Promega Biomath Calculator, choosing "dsDNA: µg to pmol"
**Pore occupancy close to 0**	The Ligation Sequencing Kit was used, and sequencing adapters did not ligate to the DNA	Make sure to use the NEBNext Quick Ligation Module (E6056) and Oxford Nanopore Technologies Ligation Buffer (LNB, provided in the SQK-LSK109 kit) at the sequencing adapter ligation step, and use the correct amount of each reagent. A Lambda control library can be prepared to test the integrity of the third-party reagents.
**Pore occupancy close to 0**	The Ligation Sequencing Kit was used, and ethanol was used instead of LFB or SFB at the wash step after sequencing adapter ligation	Ethanol can denature the motor protein on the sequencing adapters. Make sure the LFB or SFB buffer was used after ligation of sequencing adapters.
**Pore occupancy close to 0**	No tether on the flow cell	Tethers are adding during flow cell priming (FLT tube). Make sure FLT was added to FB before priming.

Observation	Possible cause	Comments and actions

Shorter than expected read length

Observation	Possible cause	Comments and actions
**Shorter than expected read length**	Unwanted fragmentation of DNA sample	Read length reflects input DNA fragment length. Input DNA can be fragmented during extraction and library prep.<br><br>1. Please review the Extraction Methods in the Nanopore Community for best practice for extraction.<br>2. Visualise the input DNA fragment length distribution on an agarose gel before proceeding to the library prep.<br><br>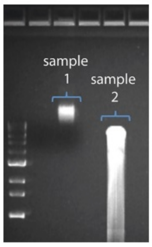<br><br>In the image above, Sample 1 is of high molecular weight, whereas Sample 2 has been fragmented.<br><br>3. During library prep, avoid pipetting and vortexing when mixing reagents. Flicking or inverting the tube is sufficient.

Large proportion of recovering pores

Observation	Possible cause	Comments and actions

Observation	Possible cause	Comments and actions
**Large proportion of recovering pores (shown as dark blue in the channels panel and duty time plot)**	Contaminants are present in the sample	Some contaminants can be cleared from the pores by the unblocking function built into MinKNOW. If this is successful, the pore status will change to "single pores". If the portion of recovering pores (unavailable pores in the extended view) stays large or increases:<br><br>1. A nuclease flush using the Flow Cell Wash Kit (EXP-WSH004) can be performed, or<br>2. Run several cycles of PCR to try and dilute any contaminants that may be causing problems.<br><br>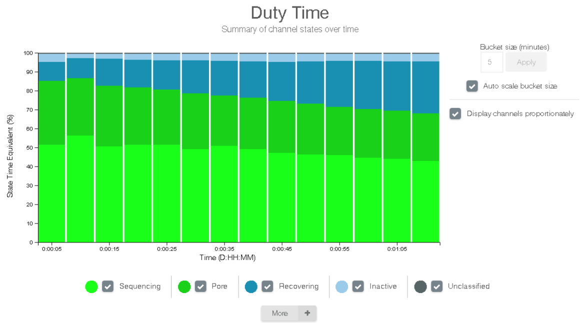<br>The duty time plot above shows an increasing proportion of "recovering" pores over the course of a sequencing experiment

Large proportion of inactive pores

Observation	Possible cause	Comments and actions
**Large proportion of inactive pores (shown as light blue in the channels panel and duty time plot. Pores or membranes are irreversibly damaged)**	Air bubbles have been introduced into the flow cell	Air bubbles introduced through flow cell priming and library loading can irreversibly damage the pores. Watch the Priming and loading your flow cell video for best practice
**Large proportion of inactive pores**	Certain compounds co-purified with DNA	Known compounds, include polysaccharides, typically associate with plant genomic DNA.<br><br>1. Please refer to the Plant leaf DNA extraction method.<br>2. Clean-up using the QIAGEN PowerClean Pro kit.<br>3. Perform a whole genome amplification with the original gDNA sample using the QIAGEN REPLI-g kit.
**Large proportion of inactive pores**	Contaminants are present in the sample	The effects of contaminants are shown in the Contaminants Know-how piece. Please try an alternative extraction method that does not result in contaminant carryover.

Reduction in sequencing speed and q-score later into the run

Observation	Possible cause	Comments and actions
**Reduction in sequencing speed and q-score later into the run**	Fast fuel consumption is typically seen when the flow cell is overloaded with library (please see the appropriate protocol for your DNA library to see the recommendation).	Add more fuel to the flow cell by following the instructions in the MinKNOW protocol. In future experiments, load lower amounts of library to the flow cell.

Temperature fluctuation

Observation	Possible cause	Comments and actions
**Temperature fluctuation**	The flow cell has lost contact with the device	Check that there is a heat pad covering the metal plate on the back of the flow cell. Re-insert the flow cell and press it down to make sure the connector pins are firmly in contact with the device. If the problem persists, please contact Technical Services.

Failed to reach target temperature

Observation	Possible cause	Comments and actions
**MinKNOW shows "Failed to reach target temperature"**	The instrument was placed in a location that is colder than normal room temperature, or a location with poor ventilation (which leads to the flow cells overheating)	MinKNOW has a default timeframe for the flow cell to reach the target temperature. Once the timeframe is exceeded, an error message will appear and the sequencing experiment will continue. However, sequencing at an incorrect temperature may lead to a decrease in throughput and lower q-scores. Please adjust the location of the sequencing device to ensure that it is placed at room temperature with good ventilation, then re-start the process in MinKNOW. Please refer to this FAQ for more information on MinION Mk 1B temperature control.

Guppy – no input .fast5 was found or basecalled

Observation	Possible cause	Comments and actions
**No input .fast5 was found or basecalled**	*input_path* did not point to the .fast5 file location	The *--input_path* has to be followed by the full file path to the .fast5 files to be basecalled, and the location has to be accessible either locally or remotely through SSH.
**No input .fast5 was found or basecalled**	The .fast5 files were in a subfolder at the *input_path* location	To allow Guppy to look into subfolders, add the*--recursive* flag to the command

Guppy – no Pass or Fail folders were generated after basecalling

Observation	Possible cause	Comments and actions
**No Pass or Fail folders were generated after basecalling**	The *--qscore_filtering* flag was not included in the command	The *--qscore_filtering* flag enables filtering of reads into Pass and Fail folders inside the output folder, based on their strand q-score. When performing live basecalling in MinKNOW, a q-score of 7 (corresponding to a basecall accuracy of ~80%) is used to separate reads into Pass and Fail folders.

Guppy – unusually slow processing on a GPU computer

Observation	Possible cause	Comments and actions
**Unusually slow processing on a GPU computer**	The *--device* flag wasn't included in the command	The *--device* flag specifies a GPU device to use for accelerate basecalling. If not included in the command, GPU will not be used. GPUs are counted from zero. An example is *--device cuda:0 cuda:1*, when 2 GPUs are specified to use by the Guppy command.

MinIT – the MinKNOW interface is not shown in the web browser

Observation	Possible cause	Comments and actions
**The MinKNOW interface is not shown in the web browser**	Browser compatibility issue	Always use Google Chrome as the browser to view MinKNOW. Alternatively, instead of typing //mt-xxxxxx (x is a number) in the address bar, type in in the generic IP address, 10.42.0.1, which identifies the MinIT Wi-Fi router.
**The MinKNOW interface is not shown in the web browser**	The MinIT Wi-Fi was not used for connecting to the computer or mobile device	Make sure the computer or mobile device is using the MinIT Wi-Fi. It should be shown as MT-xxxxxx (x is a number) on the underside label on the MinIT:<br>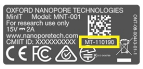<br><br>Disable the Ethernet connection from the computer or mobile device as needed. If necessary, contact your IT department to determine if the MinIT Wi-Fi is blocked (MinIT generic IP: 10.42.0.1). Please white-list MinIT as needed.
**The MinKNOW interface is not shown in the web browser**	The MinIT was not on the same network that the computer was connected to.	Make sure that the wall sockets used by the Ethernet cables from the MinIT and computer belong to the same local network.

MinIT – the MinIT software cannot be updated

Observation	Possible cause	Comments and actions
**The MinIT software cannot be updated**	The firewall is blocking IPs for update	Please consult your IT department, as the MinIT software requires access to thefollowing AWS IP ranges. Access to the following IP addresses is also needed:<br>178.79.175.200<br>96.126.99.215
**The MinIT software cannot be updated**	The device already has the latest version of the software	Occassionaly, the MinIT software admin page displays "updates available" even when the software is already up-to-date. Please compare the version listed on the admin page with the one on the Software Downloads page. Alternatively, SSH into the MinIT through a SSH Client (e.g. Bitvise or Putty, as described in the MinIT protocol) on a Windows computer or the terminal window on a Mac, run the command, *dpkg -l | grep minit*, to find out the version of the MinIT software and *sudo apt update* if an update is needed. If the issue still persists, please contact Technical Services with details of the error.
